# Harnessing highly efficient coherent polariton parametric emission in quantum confined perovskite microcavities

**DOI:** 10.1038/s41467-026-71322-1

**Published:** 2026-04-02

**Authors:** Xinyi Deng, Sanjib Ghosh, Jiepeng Song, Changhai Zhu, Chengyong Yu, Qinglin Jia, Kangshu Li, Chun Li, Xiaoxu Zhao, Xinfeng Liu, Qing Zhang

**Affiliations:** 1 School of Materials Science and Engineering, Peking University, Beijing, 100871 PR China; 2 School of Science and Engineering, The Chinese University of Hong Kong, Shenzhen, Shenzhen, 518172 PR China; 3 CAS Key Laboratory of Standardization and Measurement for Nanotechnology, National Center for Nanoscience and Technology, Beijing, 100190 PR China

**Keywords:** Polaritons, Polaritons

## Abstract

Microcavity exciton polaritons emerge as a versatile platform for nonlinear optical effects thanks to the unique dispersion which enables a manifold of energy and wavevector conserved processes. However, efficient generation of parametric emission while preserving coherence remains challenging mainly due to lack of strong parametric interactions compared to simultaneous interaction with relaxation channels. Herein, we report highly efficient parametric emission with sub-1-meV linewidth from quantum confined microcavities, enabling the first observation of strong phase coherence between the two parametric species. Quantum size effect enabled by surface disorder in confined systems acts as the microscopic mechanism for the observed anomalous enhancement of parametric emission, playing important role in triggering exotic parametric interactions for microcavity polaritons. Finally, we demonstrate the emergence of polariton supersolid phase at room temperature in a coherent parametric oscillator, characterized by periodic density modulation in real space that indicates the breaking of translational symmetry.

## Abstract (web summary)

Microcavity exciton polaritons are a promising platform for optical parametric processes. Here, authors achieve efficient parametric emission with strong phase coherence in quantum confined microcavities, demonstrating a polariton supersolid phase at room temperature.

## Introduction

Exciton-polaritons, hybrid light-matter quasiparticles formed through strong photon-exciton coupling, have established themselves as a versatile platform for investigating nonlinear quantum optics and collective coherent phenomena in driven-dissipative systems^1–4^. The inherent nonlinearity of these bosonic quasi-particles, originating from the excitonic component, enables efficient polariton-polariton scattering processes. Further enriched by the distinctive dispersion of multiple polariton bands, microcavity polariton supports a manifold of parametric interactions that circumvent conventional phase matching constraints, enabling efficient parametric emission at reduced power consumption^5–9^. Among the various types of parametric interactions, energy-degenerate optical parametric oscillation (OPO, or parametric scattering)^9–16^ has garnered significant interest for its potential to generate phase-correlated or quantum-entangled photon pairs^17–19^, given that the signal and idler states emerge from source polaritons sharing identical quantum statistics. More recently, such an OPO configuration has attracted huge interest in the study of supersolid phase of matter^20,21^, characterized by the formation of periodic density modulations in real space^22–26^, thanks to the symmetric momentum-space distribution and the interacting nature of signal and idler polaritons. Nevertheless, most experimental implementations of such configurations exhibit suboptimal parametric emission efficiencies-typically characterized by low signal-/idler-to-source polariton intensity ratios-with reported enhancements often requiring pump powers few times above threshold^11^. These improvements, however, are inevitably accompanied by spectral broadening, which degrades the coherence and interaction properties of the signal and idler polaritons. This inefficiency may arise from either insufficient nonlinear interaction strengths or rapid depletion of parametric states^12,13^, limiting the intricate application of parametric interactions and highlighting the need for strategies that simultaneously enhance emission efficiency while preserving spectral purity.

In this work, we report highly efficient and spectrally pure interbranch energy-degenerate polariton parametric emission in quantum confined CsPbBr_3_ microcavities. Dispersion characteristics enabled by surface disorder (refer to morphological roughness, in this context) and geometric confinement allow enhanced parametric interactions. Such configuration further endows high-purity polariton states on different branches to have phase-matched energy levels and increased density of states, thereby enabling efficient parametric emission while preserving sub-1-meV linewidth. Benefiting from the efficient emission, phase coherence between signal and idler polaritons in momentum space with clear visibility were observed, showing the potential for coherent interactions into the quantum regime. With respect to the mechanism that leads to strong parametric emission, corresponding experimental and theoretical studies reveal a quantum size effect and highlight the crucial role of disorder in strongly confined regime. Finally, we observed the room temperature supersolid phase utilizing the efficient coherent parametric oscillator. Our findings reveal the crucial role of quantum confinement and disorder potential landscape in facilitating exotic parametric interactions.

## Results and discussion

**Fig. 1:**
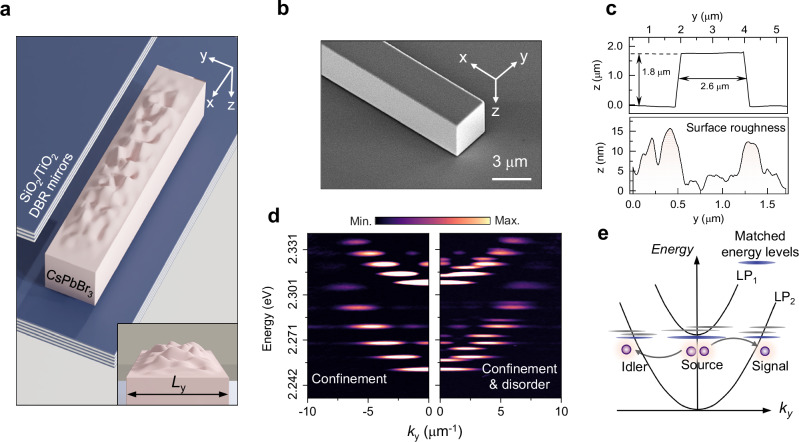
Structure schematic and exciton polariton dispersion features of quantum confined perovskite microcavities. **a** 3D schematic sketch of the CsPbBr_3_ microplatelet (MP) embedded in a planar cavity. Inset shows cross-sectional view of its typical anisotropic dimension with *y* direction dimension (*L*_*y*_) approximately 2–4 μm, and the thickness typically ranging from 1 to 3 μm. Surface disorders are formed by a stamp transfer process. **b** Scanning electron microscope (SEM) image of an anti-solvent grown CsPbBr_3_ MP with clear and smooth facets, and strong *L*_*y*_ confinement. **c** Morphology characterization of the MP. Upper panel shows laser confocal microscope characterization of the sample thickness profile. Lower panel shows a typical profile of transfer induced surface roughness. **d** Transverse magnetic (TM) polarized angle-resolved photoluminescence (PL) spectra from a microcavity with *L*_*y*_ = 3.7 μm (excitation laser: CW, 405 nm). Left panel: weak disorder, predominated by *L*_y_ confinement; right panel: moderate disorder, affected by both *L*_y_ confinement and disorder potential (Supplementary Fig. 8). **e** Schematic of the dispersion for energy-degenerate parametric scattering, where spectral-pure discretized states exist on two lower polariton branches, and efficient process happens when the polariton states (highlighted steel-blue energy levels) match with each other.

Figure 1a depicts the geometrical features of the CsPbBr_3_ microcavity, where an orthorhombic phase single-crystalline CsPbBr_3_ microplatelet (MP) was embedded in a distributed Bragg reflector (DBR) cavity. The CsPbBr_3_ was selected due to its large exciton binding energy, high photoluminescence quantum yield and good structure stability, enabling robust room-temperature polaritonics^27–30^. The MPs were fabricated by an anti-solvent route (Fig. 1b)^31^, which exhibit an anisotropic lateral dimension with *L*_*x*_ (length of *x*-axis) > *L*_y_ (length of *y*-axis). The *L*_*x*_ is generally no less than 10 μm, while *L*_y_ can be as short as around 2 μm (Supplementary Fig. 2). Nevertheless, the MPs feature smooth facets and exhibit consistent optical properties across the relevant range of lateral dimensions (Supplementary Fig. 2). Considering the polariton thermal de Broglie wavelength (1.1–1.3 μm, Methods)^32^, the influence of *L*_x_ could be neglected, and we therefore consider the spatial confinement and energy quantization according to *L*_y_ dimension^33^. Since the high-efficiency parametric emission (with a parametric ratio larger than 1 as shown in later discussions) emerges when *L*_y_ decreases to 4.5 μm, we define the regime where *L*_y_ < 4.5 μm as strongly confined and *L*_y_ > 4.5 μm as weakly confined, respectively. Before evaporation of the top DBR, the MPs were transferred to the bottom DBR with a viscous polydimethylsiloxane stamp to induce disordered surface potential as we reported before (Fig. 1c)^34^. The surface disorder with 5–15 nanometers of depth and few-hundred nanometers of lateral dimension poses trivial degradation on the cavity quality after top mirror encapsulation.

Inset on the lower right part of Fig. 1a depicts the two types of potential that affect the polariton interaction schemes: geometric confinement and surface disorder, which are reflected correspondingly in the dispersion of such microcavity (Fig. 1d, *L*_y_: 3.7 μm, excitation: CW, 405 nm). The micron-scale thickness of MPs enables multiple sets of lower polariton branches (LPBs) which further split into transverse electric (TE) and transverse magnetic (TM) branches due to material birefringence^35^. The coupled oscillator model fitting in both polarizations are depicted in Supplementary Fig. 7, showing strong coupling characteristics. To illustrate the respective roles of confinement and disorder on polariton dispersion, TM polarized PL spectra was collected from this microcavity on sites (Supplementary Fig. 8) with weak disorder (Fig. 1d, left panel) and moderate disorder (Fig. 1d, right panel). The spacing for lowest two energy levels are 6.0 meV and 7.2 meV respectively for the two LPBs depicted on left panel, consistent with the model considering *L*_y_ dimension and polariton effective mass in the small wavevector limit^2^ (Supplementary Fig. 7). For the moderate disorder regime, additional energy levels arise with an overall narrowed linewidth, making the dispersion further enriched to fulfill phase matching condition and to enhance polariton parametric interactions through lifted degeneracy^34,36–39^. Here the lowest energy level associated with disorder lies 5.46 meV above the one associated with confinement, reflecting an effective disorder height of ~8 nm (Supplementary Fig. 8)^33^. In such strongly confined cavity with dressed surface disorder, polaritons are herein considered in the ‘quantum confinement’ regime where polariton interactions are modified on de Broglie wavelength scale and give rise to anomalously strong and coherent parametric emission (Fig. 1e). When condensation occurs on ground state, acting as ‘source’ polariton, efficient parametric process initiates and scatters to neighboring branch with symmetric wavevector (**k**) position, which are referred to as ‘signal’ and ‘idler’ polaritons, respectively.

**Fig. 2:**
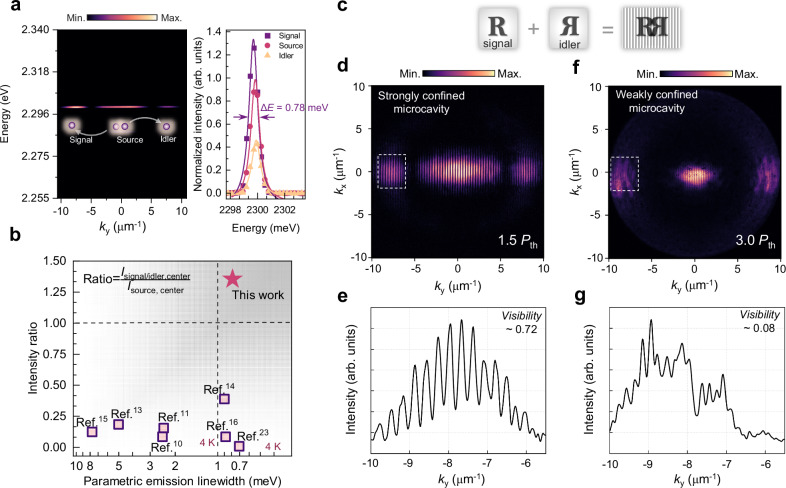
Highly efficient and coherent polariton parametric emission from quantum confined microcavities. **a** A representative parametric emission spectra where source polariton condensate from the bottom of LPB1 branch were scattered to the LPB2 branch. Right panel shows emission peak profiles centered (Supplementary Fig. 9) around source, signal, and idler polaritons, showing efficient signal and idler emission with linewidth down to ~0.78 meV. **b** A comparison of parametric emission ratio and linewidth of our work with reported literature values (based on given data^10,11,23^ or estimated from color-scale maximum^13–16^). **c** Schematic of the Michelson interference measurement where two images are superimposed mirror-symmetrically, herein the signal and idler emission on two sides in *k*-space are superimposed. **d**, **e** *k*-space interferometry patterns of parametric polaritons in a strongly confined microcavity (9.0 μJ cm^−2^, 1.5 *P*_th_), where clear interference fringes appear for superimposed signal and idler polaritons. Line profiles in dashed rectangle area are plotted in **e** and a visibility of ~0.72 could be extracted. **f**, **g** Respective interferometry and line profiles in a weakly confined microcavity (20.0 μJ cm^−2^, 3.0 *P*_th_), when parametric emission is of comparable intensity to that in **d**. Poor visibility is observed for superimposed signal and idler polaritons.

As illustrated in Fig. 2a for a MP with *L*_*y*_ = 1.9 μm, when pumped non-resonantly (1 kHz, 100 fs, 400 nm) above condensation threshold, in addition to the condensate spanning the central region in angle-resolved PL spectra, two sharp emissions corresponding to signal and idler states emerge, with intensity rivaling that of the condensate. In the energy-degenerate interbranch parametric scattering process, components near *k*_y_ = 0 would serve as the most effective source to create parametric polariton pairs at opposite in-plane wavevectors, due to the highly satisfied phase matching condition^5,7,40,41^. Therefore, we extracted the spectra from central region of the respective states (Supplementary Fig. 9), considering that the source emission is broadened due to spatial confinement^33^. As shown in Fig. 2a right panel, strong signal and idler emission exhibited linewidth down to ~0.78 meV, with signal emission reaching ~1.35 times the intensity of remaining source emission. The above-unity signal-to-source ratio suggests depletion of the source state in the stimulated regime and indicates highly efficient nonlinear parametric process, a characteristic that has likewise been reported in pure-photonic OPOs^42,43^. The depletion of source state has also been observed in intrabranch polariton OPOs in the stimulated regime^44,45^. In our theoretical study, an above-unity ratio has also been observed as presented in the following discussion on quantum size effect. An imbalance in the intensity of signal and idler emission could also be observed, which is expected in these microcavities dressed with disorder and confinement^37,46,47^, considering the asymmetry in both population and depletion pathways in the driven-dissipative system.

Furthermore, to recognize the population mechanism for nonlinear parametric emission, resonant and non-resonant pump experiments were performed in an identical microcavity (Supplementary Figs. 10–12). The comparable parametric ratio and intensity growth behavior suggested the dictating role of nonlinear parametric scattering, rather than direct condensation fed by the exciton reservoir. Meanwhile, we observed that blue shifting the source condensate via increasing pumping fluence simultaneously blueshifts the signal and idler emissions, with their wave vectors shifting towards larger values, governed by the parabolic dispersion of the LPB^22,23^ (Supplementary Fig. 13). In addition, to exclude the edge scattering effect previously observed for the laterally confined cavities, we performed real-space-resolved spectroscopy of a strongly confined microcavity and identified that the parametric emission solely comes from center of the microcavity (Supplementary Fig. 14).

To estimate the parametric emission intensity and coherence, a comparison with reported values^10,11,13–16,23^ was summarized and plotted in Fig. 2b, in respect of linewidth and parametric emission ratio. Our parametric emission with intensity rivaling that of the source condensate while maintaining sub-1-meV linewidth shows superiority as an efficient coherent polariton parametric oscillator. Such pure emission holds promise to study coherent interactions for parametric polaritons. We use *k*-space interferometry as a proof-of-concept demonstration to show its capability. Figure 2c shows the experimental configuration: the Michelson interference with images superimposing mirror-symmetrically were carried out in *k*-space, with signal and idler polariton emission on two opposite **k** directions superimposed with each other. To directly visualize the superiority of strong parametric emission with preserved coherence in quantum confinement regime, we compare the *k*-space interferometry in a strongly confined microcavity and a weakly confined cavity (both dressed with disorder) at time delay of ~0.03 ps. Figure 2d shows the momentum space interferogram for a strongly confined microcavity at 9 μJ cm^−2^ (1.5 *P*_th_) where clear interference fringes were observed for overlapping signal and idler polaritons, as circled out by white dashed boxes on one side of the Fourier plane. The visibility is calculated to be ~0.72 according to the line profile of the interference fringes (Fig. 2e). Such visibility demonstrates strong phase coherence between signal and idler polaritons in our microcavities. In contrast, for the weakly confined microcavity, when it was pumped at similar level (10 μJ cm^−2^, 1.5 *P*_th_), parametric emission is rather weak (Supplementary Fig. 15). Therefore, higher pump fluence (20 μJ cm^−2^, 3.0 *P*_th_) is needed for the generation of strong parametric emission comparable with the strongly confined microcavities (Fig. 2f). Such pump fluence inevitably leads to dephasing processes and broadening of the linewidth of signal and idler polaritons (Fig. 2f, g, Supplementary Fig. 16).

The role of confinement in enhancing polariton–polariton interactions can be anticipated from the increased wavefunction overlap and thus stronger nonlinear coupling, as has been reported in a variety of microcavity configurations^36,37,39,48^. However, in previously reported confined schemes^13,14^, the parametric emission intensity is often not substantially improved compared to planar microcavities. In our case, the disorder potential acts as the key enabler for the observation of strong coherent parametric emission. To qualitatively illustrate its role, we investigated variation of parametric emission with different levels of disorder (Supplementary Fig. 17). The markedly enhanced parametric emission in the presence of a moderate amount of disorder highlights its crucial importance in triggering efficient parametric emission. The disorder potential breaks overall inversion symmetry and thereby lifts the degeneracy for wavevector selection rules^36,39,49^, allowing the parametric process to occur under less stringent conditions.

**Fig. 3:**
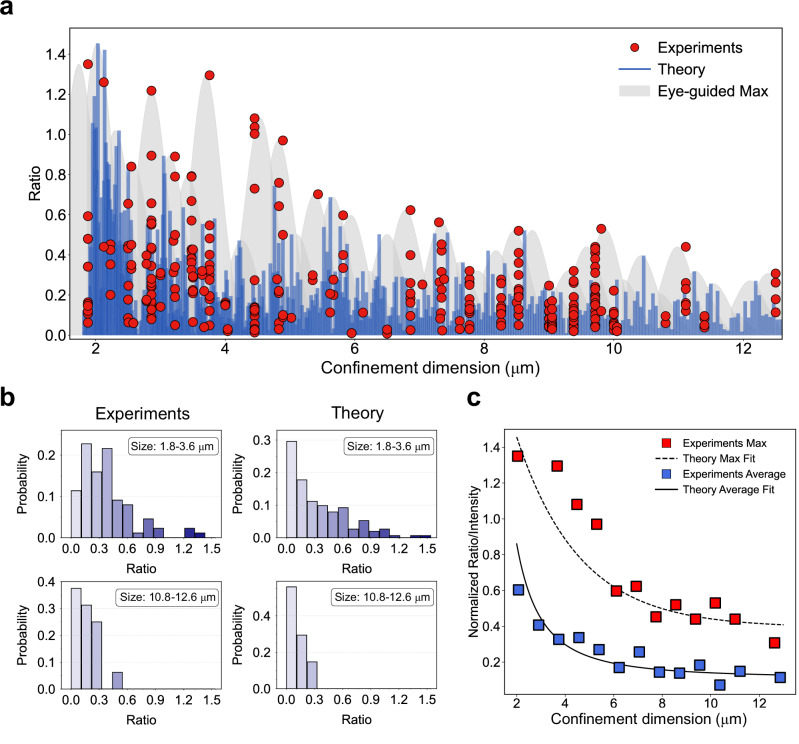
Quantum size effect mediating the parametric emission. **a** Statistical counts of scattering ratio (signal-/idler-to-source) with different geometric confinement dimensions. Red scatters denote experimental values of different samples or energy levels. Blue scatters are theoretically calculated results with various confinement and disorder configurations. An identical trend is observed that fluctuation in ratio increases when confinement dimension scales down. Gray Gaussian distribution backgrounds are for eye-guidance only. **b** Histogram comparison of scattering efficiency fluctuation level in strongly confined microcavities (width: 1.8–3.6 μm) and weakly confined microcavities (width: 10.8–12.6 μm) for experiments and theory, respectively. **c** Average and maximum of scattering efficiency with respect to confinement dimension from statistical analysis on experiments and theory. Red and blue scatters denote maximum value and average value from experiments. Dashed and solid lines are fitted line for maximum and average of theoretical calculation.

To further examine how confinement and disorder correlate and mediate parametric interactions, we compiled a statistical data set with the lateral confinement size (*L*_y_) taken as the independent variable. The confinement size (*L*_y_) can be accurately defined, whereas the degree of disorder is difficult to parameterize. For each size, all accessible disorder configurations measured are included, effectively averaging over random realizations and allowing the overall trends and systematic behavior to be revealed. The statistical analysis is shown in Fig. 3 and carried out in parallel with theory based on the driven-dissipative Gross-Pitaevskii equation as described in the Methods section. An identical trend is observed for experiments (scatters) and theoretical results (lines) that with decreasing confinement dimension, the fluctuation in parametric scattering ratio becomes stronger. Such phenomenon is typically observed in strongly interacting systems such as nano-sized superconductors^50–53^. For small particles or thin films, trivial size variation could lead to evident changes in number of energy levels near the Fermi energy, causing prominent fluctuations in the spectral density and are reflected in a gradually increasing fluctuation in order parameters like superconducting transition temperature^52^ or energy gap^53^, etc.

Adopting the scheme to our polariton OPO, we attribute such rapid fluctuation to an analogous quantum size effect. When the system size (predominated by confinement *L*_y_ in CsPbBr_3_ microcavity) becomes comparable to de Broglie wavelength^32^, disorder potential would induces a strong sensitivity of nonlinear processes to system parameters like energy level position, energy level density of states, *etc*. In this regime, quantization of energy and momentum becomes strong and starts playing significant roles in mediating parametric interaction strength. Disorder in such quantum confinement configuration induce significant fluctuations in energy positions and density of states for signal, source and idler on respective LPBs even with small perturbations, strongly affecting the phase matching conditions and final state population density to stimulate efficient parametric scattering. As a result, the parametric ratio becomes rapidly oscillating as depicted by eye-guided background peaks in Fig. 3a. The fine-energy-matching mechanism could also be reflected in the gradually decreasing energy mismatch of signal and source, idler and source with increasing pump fluence (Supplementary Fig. 18) and acquisition of highly efficient parametric emission in strongly confined microcavities with small mismatch (shown in Supplementary Fig. 19 in a histogram map that correlates energy mismatch into the counted ratio).

Disorder is present in all cavities, but its impact becomes most pronounced when confinement approaches dimension comparable to the polariton de Broglie wavelength. To quantify the fluctuations and accessible ratios across confinement regimes, we select two confinement ranges (1.8–3.6 μm, and 10.8–12.6 μm) and plot the corresponding fluctuation level in probability histogram. For strongly confined regime (upper panel), the histogram distributes in a broad ‘ratio’ range and exhibits a probability amplitude that decreases with larger ‘ratio’. However, in weakly confined systems (lower panel), such histogram mainly distributes in a small ‘ratio’ range on the lower value side. Although the distribution shown in Fig. 3a, b refers to the randomness in emission intensity, a statistical analysis on its maximum and average value is meaningful in finding the expectation values in obtaining an efficient parametric oscillator, as shown in Fig. 3c. An increasing trend is observed for both maximum (red) and average (blue) values in scattering efficiency when decreasing confinement, indicating that the role of disorder is fully revealed in the strongly confined regime.

**Fig. 4:**
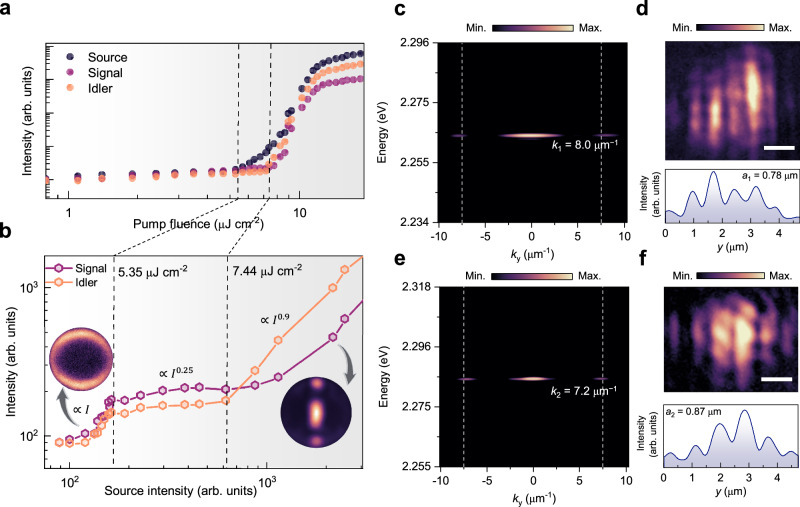
Room temperature supersolidity from the parametric oscillator. **a** Pump-fluence-dependent intensity evolution of source, signal and idler polaritons, with two nonlinear thresholds shown by black dashed lines. **b** Relationship of source intensity and signal/idler intensity near the two nonlinear thresholds. Different growth behaviors suggest different scattering processes with incoherent and coherent nature. Inset shows *k*-space distribution for the respective processes, changing from uniform dominant exciton reservoir distribution (left inset) to coherent parametric scattering from source to signal/idler polaritons (right inset, no additional spectral filtering was applied). **c** Angle-resolved PL spectra of parametric oscillator with signal/idler centered around *k*_1_ = 8.0 μm^−1^. **d** Upper panel: real space image of the parametric oscillator with periodic fringes. Lower panel: extracted line profiles from the real space image, showing periodicity *a*_1_ = 0.78 μm, corresponding to momentum space of parametric emission in **c**. **e** Angle-resolved PL spectra of parametric oscillator with signal/idler centered around *k*_2_ = 7.2 μm^−1^. **f** Upper panel: real space image of the parametric oscillator with periodic fringes. Lower panel: extracted line profiles from the real space image, showing periodicity *a*_2_ = 0.87 μm, corresponding to momentum space of parametric emission in **e**. Scale bars in **d** and **f**: 1 μm.

The coherent interacting nature, combined with conjugated *k*-space position of the parametric polaritons makes energy-degenerate OPO an ideal platform to investigate crystallized supersolid phase of the quantum fluid of light. The recently observed supersolidity of polariton OPO in GaAs at liquid helium temperature is a crucial step towards this goal^22,23^. Benefitting from the large exciton binding energy and robust light-matter interaction of CsPbBr_3_ at room temperature^27,29,30,34,54,55^, we realize such supersolidity at ambient conditions, substantiated by the observation of two thresholds in parametric emission growth behavior, and coordinate space pattern formation with a period correlated with **k** position of the parametric polariton. As illustrated in Fig. 4a, the evolution of intensity exhibits two nonlinear thresholds (marked by black dashed lines), with sub-linear growth at low pump fluence and subsequent nonlinear growth of source (5.35 μJ cm^−2^) and signal/idler (7.44 μJ cm^−2^), respectively. Different growth behavior of parametric emission with respect to source BEC density (*I*) is retrieved by examining the respective intensity evolution closely near the two thresholds, as shown in Fig. 4b and denoted by *I*, *I*^0.25^, and *I*^0.9^. Below the first threshold, incoherent relaxation from the exciton reservoir, and linear scattering from source polariton is responsible for the population at signal and idler states. Crossing the first threshold, source condensate first exhibits nonlinear growth. With source condensate population building up, nonlinear parametric scattering was initiated and nonlinearly increases the signal and idler intensity, accompanied by a dramatic reorganization in momentum space as shown in the insets of Fig. 4b. The left inset depicts a uniform exciton reservoir distribution, while the right inset highlights coherent parametric scattering between the source condensate and conjugate signal/idler polaritons. Respective angle-resolved PL spectra and momentum space images for this microcavity at different pump fluences are depicted in Supplementary Fig. 20.

As shown in Fig. 4c–f, for two parametric oscillators with angle-resolved PL spectra (Fig. 4c, e) showing signal and idler polaritons centered at distinct wavevectors (8.0 μm^−1^ and 7.2 μm^−1^), corresponding features were observed in real space emission patterns. The real space images (Fig. 4d, f, upper panels) with striking periodic fringes confirm a ‘lattice’ constant of 0.78 μm and 0.87 μm by extracting the line profiles (lower panels). Such spatial periodicity aligns precisely with the momentum-space emission peaks via Fourier reciprocity, confirming the spontaneous ‘crystallization’ of the polariton condensate and its supersolid nature. We believe that such coherent parametric emission with intensity comparable to source condensate enables direct observation of an anomalous supersolid phase with periodic stripes in real space, which would otherwise be obscured by strong source condensate emission. In strongly confined microcavities, such phenomenon with correlated momentum space position and real space fringe spacing was also observed (Supplementary Fig. 21), except with presence of limited number of fringes due to the finite dimensionality. Circumventing the extreme cooling requirements of traditional quantum fluids establishes the optical parametric oscillator in our microcavity as an ideal testbed for emergent macroscopic quantum effects.

In summary, this work establishes quantum confined semiconductor microcavities as a robust platform for strong coherent parametric polariton generation through the synergistic interplay of geometric confinement and surface disorder. Highly efficient parametric emission is achieved in presence of disorder in the strongly confined regime, with parametric emission intensity rivaling those of the source condensate, while preserving high spectra-purity with sub-1-meV linewidth. The ultra-narrowband emission enables the observation of phase coherence between signal and idler polaritons. Statistical analysis from experiments and theory reveals the role of quantum size effect in mediating parametric emission intensity in the quantum confined microcavity. In addition, the efficient emission enables direct observation of emerging supersolid phase at room temperature. These results demonstrate that non-trivial potential landscape act as key enablers of unconventional parametric phenomena—which could be readily extended to other material platforms, providing transformative strategies to harness polariton nonlinearities and accelerate the development of quantum photonic devices.

## Methods

### Perovskite Synthesis

The CsPbBr_3_ microplatelets were synthesized using an anti-solvent method^31^. CsBr and PbBr_2_ powders of analytical grade were mixed in a 1:1 molar ratio and dissolved in dimethylformamide to prepare a 20 mM precursor solution. Glass substrates were placed in a dichloromethane anti-solvent filled beaker, and the precursor solution was dropped onto the substrate, as shown in Supplementary Fig. 2a. Beaker was covered with perforated Parafilm and placed in an oven with temperature of 30 °C for 48 h, enabling evaporation and CsPbBr_3_ single crystal growth. The synthesis procedure yields microplatelets of various lateral dimensions, shown in Supplementary Fig. 2b.

### Microcavity Fabrication

The CsPbBr_3_ microcavity consists of top and bottom distributed Bragg reflector (DBR) mirrors and central CsPbBr_3_ microplatelets. Bottom DBR consists of 20 pairs of electron-beam evaporated titanium oxide (TiO_2_) and silicon dioxide (SiO_2_) with quarter-wavelength thickness, and top DBR consists of 8 pairs of such oxide layers. CsPbBr_3_ microplatelets were dry transferred to bottom DBR with polydimethylsiloxane (PDMS) stamps, followed by ~60 nm PMMA layer encapsulation before top mirror evaporation, acting as protection layer for perovskites.

### Optical Characterization

Optical characterization was conducted using a custom-built system based on Fourier imaging, allowing for both real-space and momentum-space resolved spectroscopy and imaging. For real space imaging and spectroscopy on the micron-scale sized sample, a short-focal-length lens was used to magnify the image, to resolve emission from the microplatelet microcavity. A Michelson interferometer with mirror symmetric configuration was utilized to investigate first-order spatial and temporal coherence in both real space and *k*-space. Pump laser spot size was enlarged by a long-focal-distance lens in the excitation path, as shown in Supplementary Fig. 6. Emission from the sample was captured with a 100× objective (NA = 0.9) and spatially filtered with an adjustable rectangular aperture at the focal plane and directed to a liquid nitrogen-cooled charge-coupled device (CCD) spectrometer (Princeton Instruments, SP-2500i). For pulsed excitation, the 800 nm fundamental pulses (100 fs, 1 kHz) from a Coherent Astrella regenerative amplifier were directed to a beta barium borate (BBO) crystal to generate non-resonant pump pulses at 400 nm, or directed to an optical parametric amplifier (Coherent OperA Solo) to generate resonant pump pulses at 540 nm. Continuous-wave excitation was performed using a 405 nm laser.

### Other characterizations

Atomic force microscopy (AFM) imaging was carried out in tapping mode with a BRUKER Dimension ICON system, while laser confocal microscopy was performed using a Zeiss LSM800 Airyscan confocal microscope. X-Ray diffraction (XRD) measurement was performed with Rigaku XtaLAB PRO. Annular dark-field scanning transmission electron microscopy (STEM) characterization was performed with Titan Cubed Themis G2 at 300 kV voltage. SEM images were obtained from Thermo Fisher Helios G4 UX DualBeam system.

### Theoretical model

Our theoretical description of parametric interaction in a microcavity is based on two lower polariton branches (labeled by the index ${n}=1,\,2$), which originates from the strong coupling of excitons with two different cavity modes. Each polariton branch in the two-dimensional microcavity planes follows the two-component driven-dissipative Gross-Pitaevskii equation:

1 $${i}{{\hslash }}\frac{{\partial }{{\psi }}_{{n}}\left({{\mathbf{r}}},{t}\right)}{{\partial }{t}}=	 \left[ {{\hat{E}}}_{{n}}^{{{\mbox{LP}}}}+{{V}}_{{{\mbox{dis}}}}\left({{\mathbf{r}}}\right) - \frac{i}{{2}} \left( {\gamma } - {{P}}_{{n}}\left({{\mathbf{r}}},{t}\right) \right) - {i}{\Gamma} {\left| {{\psi }}_{{n}}\left({{\mathbf{r}}},{t}\right) \right|}^{{2}} \right] {{\psi }}_{{n}}\left({{\mathbf{r}}},{t}\right) \\ 	+{{2}}{\alpha }{{\psi }}_{{m}}{\left({{\mathbf{r}}},{t}\right)}^{{2}}{{\psi }}_{{n}}^{*}\left({{\mathbf{r}}},{t}\right)+{{2}}{\alpha }{{\psi }}_{{m}}{\left({{\mathbf{r}}},{t}\right)}^{{2}}{{\psi }}_{{n}}\left({{\mathbf{r}}},{t}\right)$$

where ${E}_{n}^{{{\rm{LP}}}}\left(k\right)$ is the kinetic energy operator of the $n$-th lower polariton branch, ${V}_{{{\rm{dis}}}}({{\bf{r}}})$ represents the disorder potential in the microcavity, *γ* represents decay, and ${P}_{n}({{\bf{r}}},t)$ represents the net gain to the lower polariton branches. The kinetic energy operator is given by,

2 $${{E}_{n}^{{{\mbox{LP}}}}\left({k}\right)=\frac{{\left[ {{E}_{n}^{{{\mbox{cav}}}}\left({k}\right)+{E}_{{{\mbox{ex}}}} - \sqrt{{{\left( {{E}_{{{\mbox{cav}}}}\left({k}\right) - {E}_{{{\mbox{ex}}}}} \right)}^{{2}}}+{{\Omega }}^{{2}}}} \right]}}{{2}}}$$

where ${E}_{n}^{{{\rm{cav}}}}\left(k\right)$ represents the *n*-th cavity mode energy, *E*_ex_ represents the exciton energy and $\Omega$ is the Rabi-splitting energy. The parametric scattering effects are induced by the nonlinear interaction between polaritons that are represented by the parameter *α*. The nonlinear decay term Γ represent the saturation of the polariton density due to the effective reduction of the exciton-reservoir induced gain upon the significant polariton density. Detailed parameter values for the theoretical model are provided in Supplementary Information.

### Polariton de Broglie wavelength

In our microplatelet microcavities, polariton effective mass is 1.1 × 10^−5^ *m*_e_**–**1.5 × 10^−5^ *m*_e_ for most LPBs of interest for parametric emission. Therefore, polariton thermal de Broglie wavelength^32^ ($({{\lambda }}_{{{\mbox{dB}}}}=\sqrt{{{2}}{\pi }{{\hslash }}^{{2}}{{\left( {{m}_{{{\mbox{eff}}}}{k}_{{{\mbox{B}}}}{T}} \right)}}^{{-1}}})$) is 1.1**–**1.3 μm.

## Supplementary information

Supplementary Information

Transparent Peer Review file

## Data Availability

All data to evaluate the conclusions are present in the manuscript, and the Supplementary Information. Raw data are available from the corresponding authors upon request.
