# Supplementary material for: Harnessing highly efficient coherent polariton parametric emission in quantum confined perovskite microcavities
Source: Nat Commun. 2026 Apr 2;17:5294. doi: 10.1038/s41467-026-71322-1 (PMC13270094; doi:10.1038/s41467-026-71322-1)
Supplement: Supplementary file 1 — Supplementary Information [file 41467_2026_71322_MOESM1_ESM.pdf]

Supplementary Information for

Harnessing highly efficient coherent polariton parametric emission in quantum confined perovskite microcavities

Xinyi Deng[1†], Sanjib Ghosh[2†], Jiepeng Song[1], Changhai Zhu[1], Chengyong Yu[2], Qinglin Jia[1], Kangshu Li[1], Chun Li[1], Xiaoxu Zhao[1], Xinfeng Liu[3*], Qing Zhang[1*]

[1]School of Materials Science and Engineering, Peking University, Beijing 100871, P.R. China

[2]School of Science and Engineering, The Chinese University of Hong Kong, Shenzhen, Shenzhen 518172, P.R. China

[3]CAS Key Laboratory of Standardization and Measurement for Nanotechnology, National Center for Nanoscience and Technology, Beijing 100190, P.R. China

[†]These authors contributed equally: Xinyi Deng and Sanjib Ghosh.

*Corresponding author. Email: q_zhang@pku.edu.cn; liuxf@nanoctr.cn

Supplementary Note 1. Cavity quality

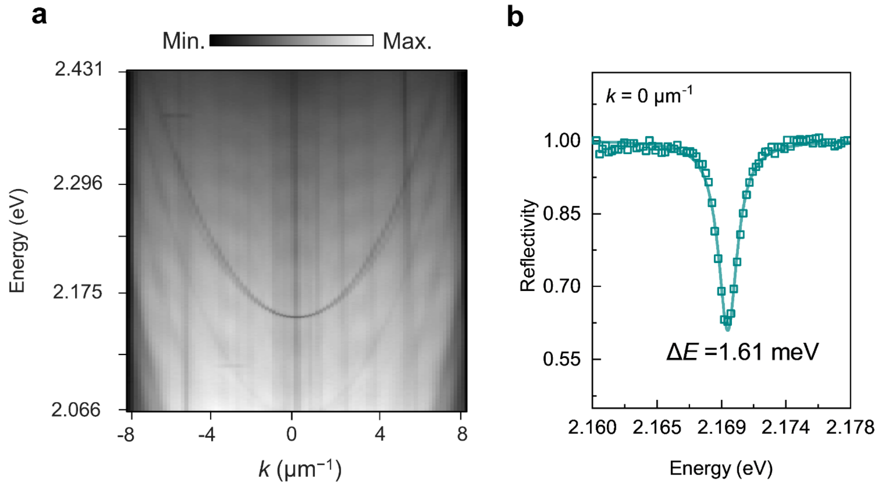

Supplementary Fig. 1| (a) Angle-resolved reflectivity of the empty cavity. (b) Extracted reflection spectrum at 0 μm^{-1} from (a), showing a linewidth of 1.61 meV and Q factor of ~1300.

Supplementary Note 2. Fabrication and characterization of the $CsPbBr_3$ microplatelets

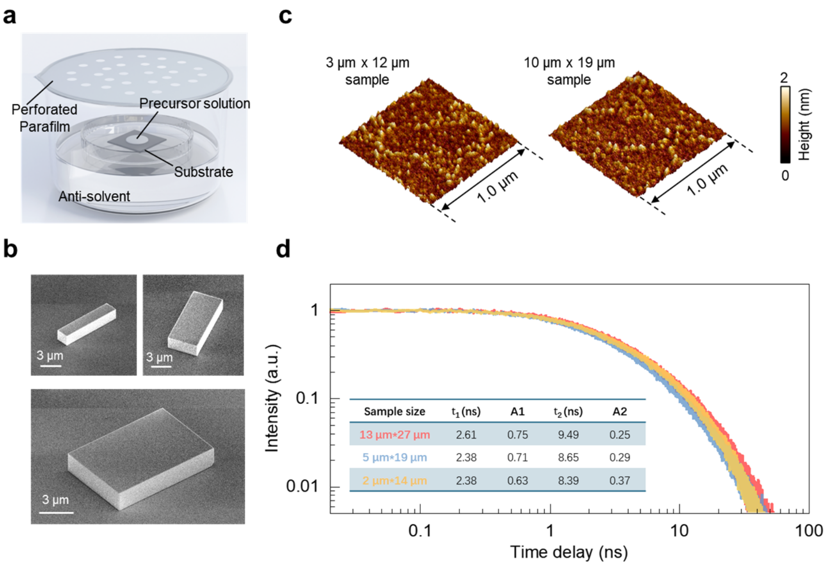

Sample size	t_1 (ns)	A1	t_2 (ns)	A2
13 μm*27 μm	2.61	0.75	9.49	0.25
5 μm*19 μm	2.38	0.71	8.65	0.29
2 μm*14 μm	2.38	0.63	8.39	0.37

Supplementary Fig. 2| (a) Schematic of anti-solvent synthesis[1] procedure. (b) SEM images for tilted view of $CsPbBr_3$ microplatelets before PDMS transfer. (c) AFM measurement of surface roughness profiles for the microplatelets with different lateral dimensions. (d) The time-resolved PL spectra for the microplatelets at pump fluence of 1 μJ cm^{-2}. Excitation laser: 515 nm, 50 kHz, 250 fs.

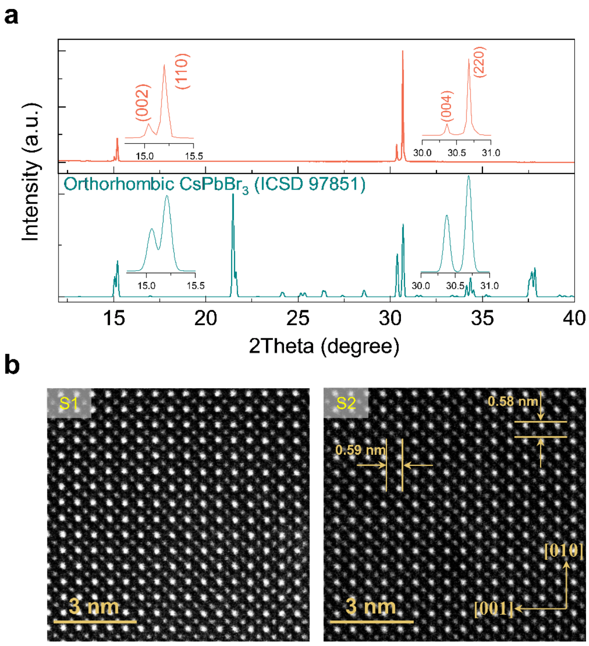

Supplementary Fig. 3| (a) XRD characterizations of the $CsPbBr_3$ microplatelets. Diffraction peaks are labelled with crystallographic planes, corresponding to

orthorhombic phase $CsPbBr_3$ (ICSD 97851) shown in lower panel. Insets show zoom-in peak profiles. (b) Annular dark field scanning transmission electron microscopy image of $CsPbBr_3$ microplatelets with different lateral dimensions, viewing from the [010] zone axis, showing an identical crystalline structure of orthorhombic $CsPbBr_3$[2]. Atomic-resolution images reveal an inter-plane spacing of 0.58 nm between the [010] planes and 0.59 nm between the [001] planes, respectively.

Supplementary Note 3. $CsPbBr_3$ absorption, PL, & polariton lasing spectra

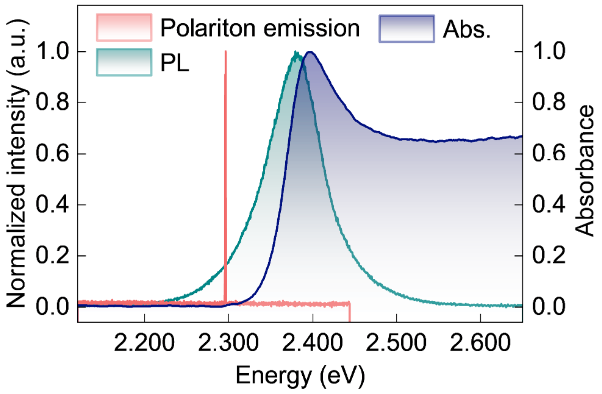

Supplementary Fig. 4| Absorbance, and PL spectra of bare $CsPbBr_3$, and polariton condensate emission in a $CsPbBr_3$ microcavity.

Supplementary Note 4. Surface disorder profile

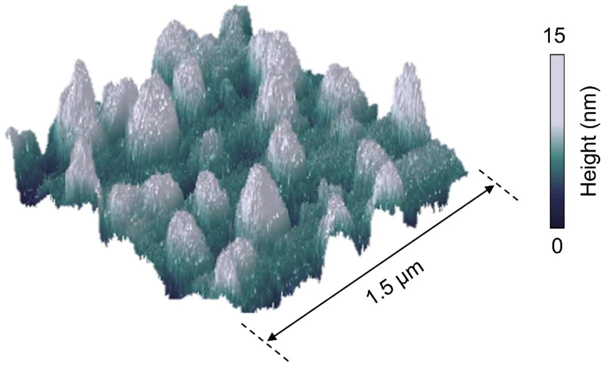

Supplementary Fig. 5| Atomic force microscope measured surface morphology of a $CsPbBr_3$ microplatelet after PDMS dry transfer[3], with highly disordered profile. Pseudo-colors denote height profile.

Supplementary Note 5. Schematic diagram of the Fourier-space imaging setup

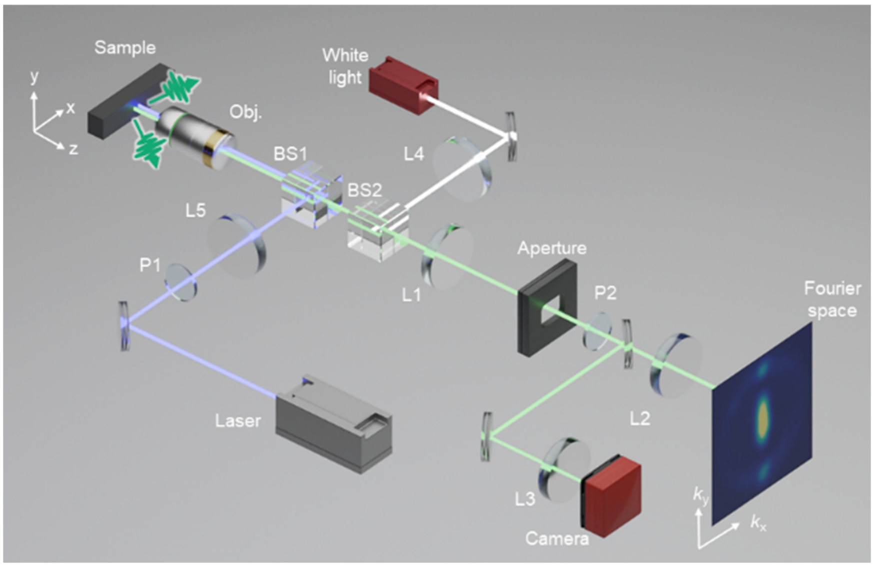

Supplementary Fig. 6| $CsPbBr_3$ microplatelet (gray cube in upper left corner) and respective signal and idler polariton (green arrows near the sample, and bright high-*k* spots in Fourier space) are depicted. (Obj: objective, L: lens, P: polarizer, BS: beam splitter) L5 denote the long focal distance lens utilized for enlarging the spot size to ~50 µm. An adjustable rectangular aperture was used to spatially select the emission from the entire MP microcavity except its edges, with cavity dimensions ranging from ~2 µm to ~15 µm, as shown in Fig. 3. (Graph rendered in Blender using assets by Ryo Mizuta Graphics)

Supplementary Note 6. Strong exciton photon coupling in thick, confined $CsPbBr_3$ microcavity and evaluation of confinement induced energy levels

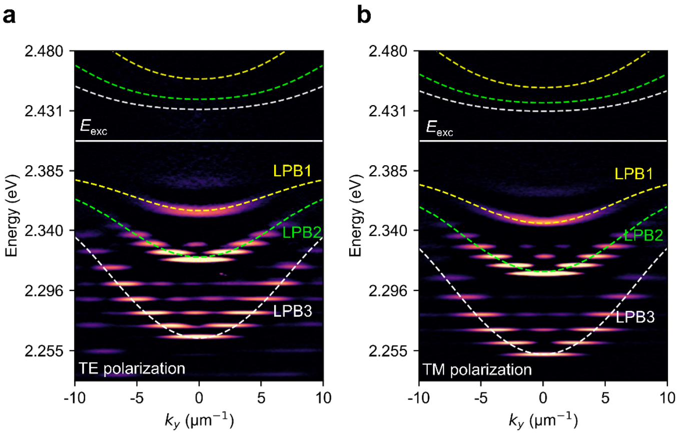

c

TM polarization	LPB1	LPB2	LPB3
Rabi splitting (meV)	119	120	127
Effective mass (*m_ex 10^{-5})	2.031	1.404	1.160
$\Delta E_{1,2}$ (meV, experiment)	/	6.0	7.2
$\Delta E_{1,2}$ (meV, calculation)	3.98	5.75	6.96

Supplementary Fig. 7| The microcavity (weak disorder) is with dimension of L_y =3.7 μm, and L_x = 12.6 μm. Excitation: CW, 405 nm). (a) Angle-resolved PL spectra for TE polarization. The colored dashed lines: respective dispersion fitting for lower polariton branches (LPBs) based on coupled oscillator model. (b) Angle-resolved PL spectra for TM polarization with dashed lines fitting the respective LPBs, solid line indicates exciton energy position. (c) Fitted Rabi splitting, LPB effective mass, and the as-measured/calculated energy level spacing for the lowest two energy levels of LPB1, LPB2 and LPB3 shown in (b), respectively.

The coupled oscillator model fitting shown in dashed lines in Supplementary Fig. 7 a and b demonstrate strong coupling features in the $CsPbBr_3$ MP cavity. The respective energy level spacing associated with each LPB could be identified as follows.

In a microcavity with micron-scale lateral confinement, energy of the polariton could be written as[4,5]

$$E_{n_x,n_y} = \frac{\hbar^2\pi^2}{2m^*}\left(\frac{n_x^2}{L_x^2}+\frac{n_y^2}{L_y^2}\right)+V_0 \tag{1}$$

where E is the energy of LPB, m^* as the effective polariton mass, $n_{x,y}$ as the quantum number for quantized lower polariton branches, $L_{x,y}$ as the confinement size for x and y directions, V_0 is the potential depth. Considering the lateral profile in our $CsPbBr_3$ as described in main text, the x-direction behavior could be treated as a free particle with continuous momentum k_x while the y-direction experiences confinement and energy level quantization. The energy difference between adjacent states becomes

$$\Delta E = \frac{\hbar^2 \pi^2}{2m^* L_y^2}\left(2n_y + 1\right) \tag{2}$$

For the lowest two energy levels (n = 0 and n = 1), the relationship becomes

$$\Delta E = \frac{3\hbar^2 \pi^2}{2m^* L_y^2} \tag{3}$$

similarly for higher energy levels with n of a larger quantum number. Therefore, fitted LPB dispersion could help identify the confinement induced energy levels. In the microcavity as shown in Supplementary Fig. 7, the polaritons for LPB2 and LPB3 have effective mass of 1.404×10^{-5} m_e and 1.160×10^{-5} m_e, respectively, and the calculated lowest energy spacing (in small wave vector limits[6]) for the two branches are 5.75 meV and 6.96 meV, close to the measured mode spacing of 6.0 meV and 7.2 meV.

The as-measured mode spacings are comparable with calculated values $\Delta E \approx \frac{3\hbar^2 \pi^2}{2m^* L_y^2}$ for LPB2 and LPB3. For LPB1, however, the effective mass is large, so the mode spacing of the lowest two energy levels is further reduced to 3.98 meV, close to its linewidth of 3.0 meV. Therefore, the quantization of the LPB1 can no longer be resolved.

Supplementary Note 7. Identification of energy levels associated with confinement and disorder, and evaluation of disorder induced energy levels

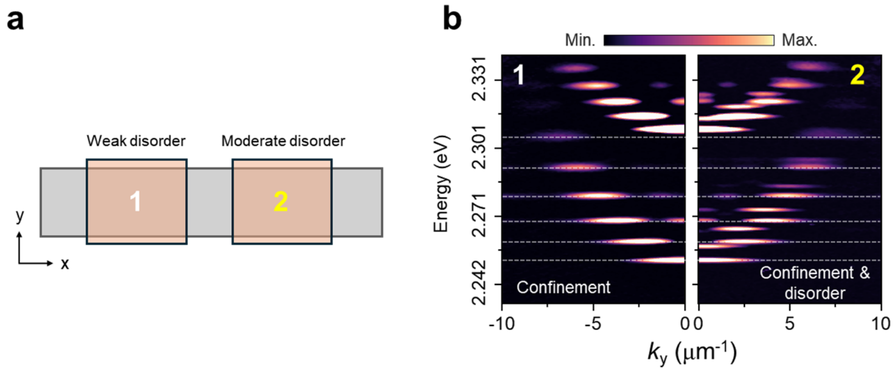

Supplementary Fig. 8| (a) Schematic for collection sites from a confined microcavity, with (1) weak surface disorder and (2) moderate surface disorder. (b) Angle-resolved PL spectra (TM polarized) from the sites in (a), where region (1) emission is mainly from L_y confinement, and region (2) emission stems from both confinement and disorder. Gray dashed lines highlight the energy levels on this LPB associated with L_y confinement.

For a photonic description of the disorder-induced energy levels in microcavities[5], we consider the disorder as a thickness variation of quantum-well layer in the DBR cavity, where the surface perovskite is locally replaced by PMMA. Within a classical-wave picture, the transfer-matrix method[7] could be used to estimate the resulting LPB resonance shift (in meV) and the corresponding disorder height/depth (in nanometers). As an example, the calculation shows that for a cavity with perovskite thickness of 2.0 μm, one of the resonant modes appear at 2305.10 meV. When the surface 10 nm of the perovskite (n ~ 2.5 at 2305.10 meV[8]) layer is replaced with PMMA (n ~ 1.45), the resonance position shift to 2311.30 meV, giving an energy shift of 6.20 meV in LPB bottom, effectively as the potential depth of the 10 nm disorder.

In Supplementary Fig. 8b right panel, the lowest energy associated with disorder lies 5.46 meV above the LPB bottom, reflecting an effective disorder height of ~ 8 nm[5,7].

Supplementary Note 8. Schematic for extraction of spectrum in Fig. 2a, and evaluation of parametric ratio

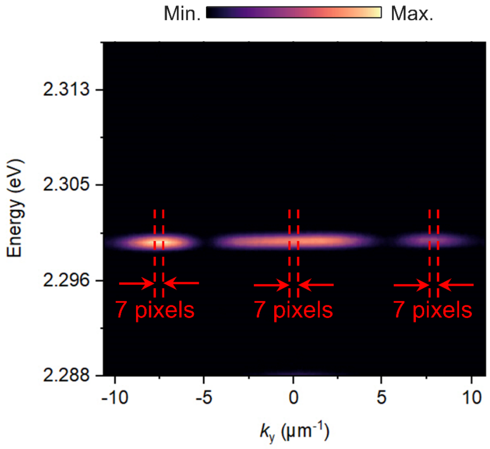

Supplementary Fig. 9| Angle-resolved PL spectra showing areas used for integration and evaluation of parametric ratio. The angle-resolved emission collected from the objective (100x, NA=0.9) was mapped to 166 pixels (angular direction) on the Fourier plane in the spectrometer. The emission centered around (red dashed lines) the respective states with 7 pixels were integrated in the angular direction for evaluation.

Supplementary Note 9. Identification of signal and idler state population mechanism

Population of signal and idler states stem from few different mechanisms. Firstly, it could be populated by exciton reservoir relaxation. Secondly, linear scattering could populate the phase matched states on LPB2. Thirdly, nonlinear parametric scattering can contribute in the stimulated regime. In non-resonant excitation, all three mechanisms play roles in the population of signal and idler. To identify the mechanism for signal/idler population observed in the strong coherent emission, we performed respective analysis for an identical sample with non-resonant and resonant pump and assigned the main contributing mechanism for the strong coherent emission as nonlinear parametric scattering from the source polariton.

For non-resonant pump, a 400 nm (1 kHz, 100 fs) pulsed laser is directed onto the sample. For resonant pumping, a 540 nm (1 kHz, 100 fs) pulsed laser is derived from an optical parametric amplifier and filtered with sets of tunable filters, to have the laser energy slightly blue-detuned from the source condensate, while also avoiding excitation of the exciton reservoir. Polarizers and half wave plates are utilized to tune laser polarization. The resonant laser beam spot size and incidence angle in Fourier space are tuned (Supplementary Fig. 10a) by an angle-variable lens, enabling focused injection near LPB1 source polariton position[9-11]. In the collection path, sets of tunable bandpass filters were tuned to allow collection at LPB1 source position while filtering out most of the incidence laser (Supplementary Fig. 10b).

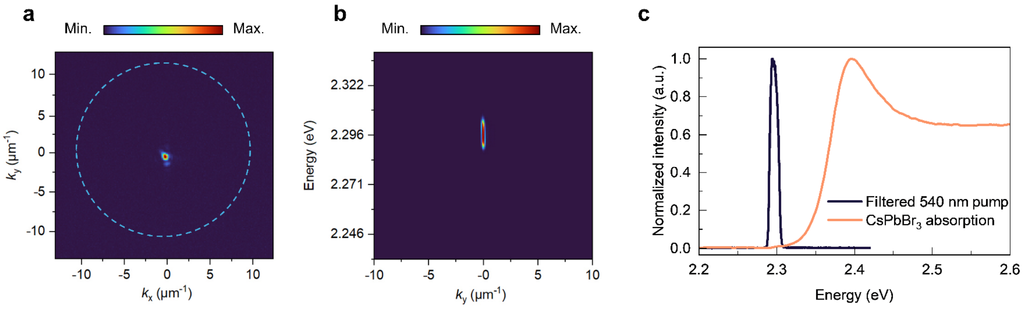

Supplementary Fig. 10| Schematic for resonant excitation on lower polariton branches. (a) Focused resonant pump laser in Fourier space, where the incidence angle could be tuned by an angle-variable lens. (b) Angle-resolved spectrum for the pump pulse, filtered by sets of tunable filters. (c) Spectrum for the filtered pump pulse and $CsPbBr_3$ absorption spectrum.

As shown in Supplementary Fig. 11a, under non-resonant pumping condition, the population on both LPB1 and LPB2 emerges in the linear regime, with discretized energy levels arising from confinement and disorder. Above the nonlinear parametric emission threshold (Supplementary Fig. 11b, the second threshold), the signal and idler emerge at energy positions resonant with the source emission. The corresponding growth rate of signal and idler intensity with respect to source emission intensity is depicted in Supplementary Fig. 11c. For the resonant pump condition, Supplementary Fig. 11d depicts the angle-resolved PL spectra below the nonlinear parametric scattering threshold (laser position circled out). The flat and narrow emission, highlighted by white arrows and just below the excitation laser energy, is the source condensate. While at this stage, the emission on the LPB2 at the respective energy position is missing. As the power further increased to higher pump fluence (Supplementary Fig. 11e), parametric emission appears at the LPB2 dispersion positions resonant with source condensate. The corresponding growth rate of signal and idler intensity with respect to source emission intensity is depicted in Supplementary Fig. 11f. The non-resonantly pumped case features a two-threshold behavior (Supplementary Fig. 11c), while resonantly pumped case exhibits a single-threshold behavior (Supplementary Fig. 11f).

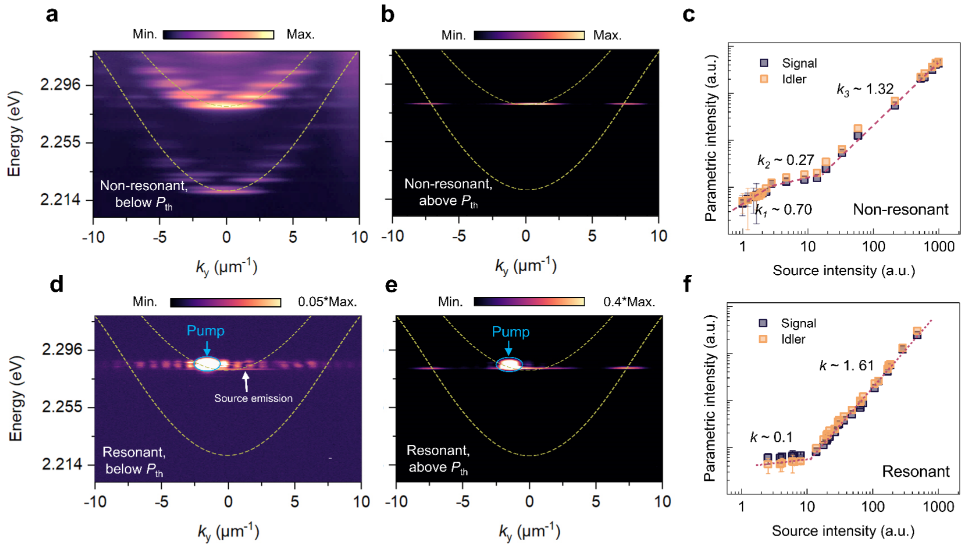

Supplementary Fig. 11| (a-c) Parametric emission under non-resonant pumping condition. a: angle-resolved PL spectra below the condensation threshold; b: angle-resolved PL spectra with parametric emission; c: the respective growth behavior of signal/idler emission intensity with respect to source polariton. (d-f) Parametric emission under non-resonant pumping condition; d: angle-resolved PL spectra below

the condensation threshold; e: angle-resolved PL spectra with parametric emission; f: the respective growth behavior of signal/idler emission intensity with respect to source polariton. The error bar represents the standard deviation.

Different processes could be identified as they exhibit different growth behavior for parametric polaritons with respect to the source polariton. Upon non-resonant excitation, below the first threshold (condensation threshold), the population of the signal and idler states is mainly supplied by incoherent relaxation from the exciton reservoir and by linear scattering from the source polaritons. Once the first threshold is crossed, the source condensate starts to grow nonlinearly. Detailed intensity, linewidth, and blueshift behavior with respect to pump fluence is shown in Supplementary Fig. 12. As its population builds up, nonlinear parametric scattering is triggered and drives a nonlinear increase of the signal and idler intensities, indicated by the second threshold in Supplementary Fig. 11c.

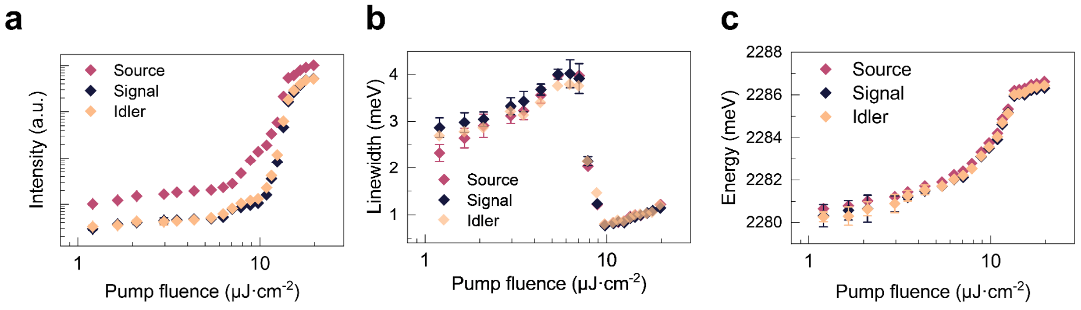

Supplementary Fig. 12| Power dependent behavior of source/signal/idler in non-resonant pumped case. (a) Pump fluence dependent intensity growth for source/signal/idler, respectively. (b) Linewidth evolution. (c) Energy blueshift. The error bar represents the standard deviation.

In resonant excitation, the exciton reservoir is excluded, therefore the population solely comes from linear scattering and nonlinear stimulated scattering. These processes could be separated as they exhibit different growth behavior for parametric polaritons with respect to the source polariton, as indicated by the transition in slope (Supplementary Fig. 11f).

Therefore, we attributed the mechanism that leads to nonlinear signal and idler emission as nonlinear scattering from source polariton. As evidenced by the comparable parametric emission ratio, and the respective growth rate in the final regime, suggesting

that identical mechanism dominated such processes. The effective slope ($k \sim 1.61$) upon resonant excitation is slightly larger than that under non-resonant ($k \sim 1.32$) excitation, due to a relatively stronger nonlinearity when the source condensate is quasi-directly fed by the fs-laser pulses.

Supplementary Note 10. Other evidence for parametric emission

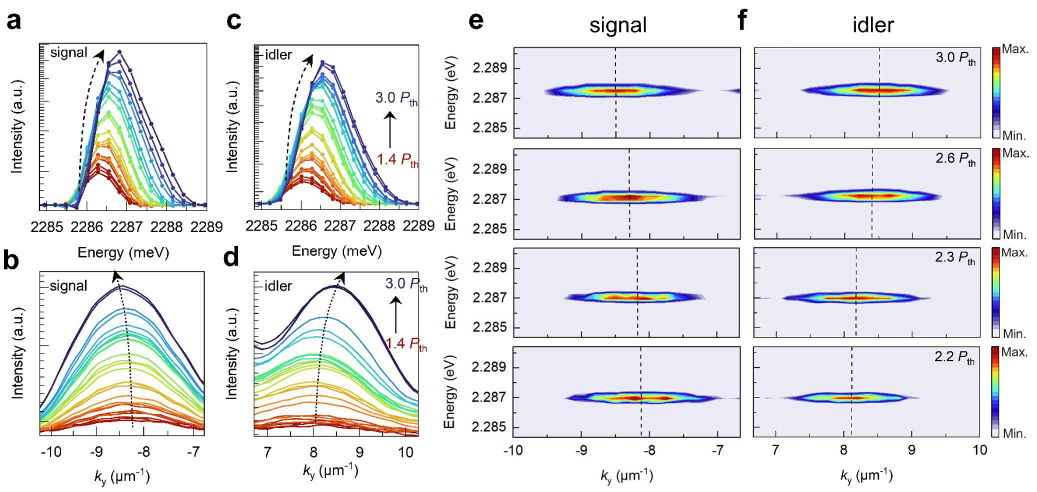

Supplementary Fig. 13| (a) Energy-resolved spectra for the 'signal' emission with increasing pump fluence. (b) Wavevector-resolved spectra for the 'signal' emission extracted at peak center (maximum) in (a). (c) Energy-resolved spectra for the 'idler' emission with increasing pump fluence. (d) Wavevector-resolved 'spectra' extracted at energy positions in (c). (e-f) Zoomed-in angle-resolved PL emission of (e) signal and (f) idler at four different pump densities, with black solid line as reference to highlight relative wavevector shift.

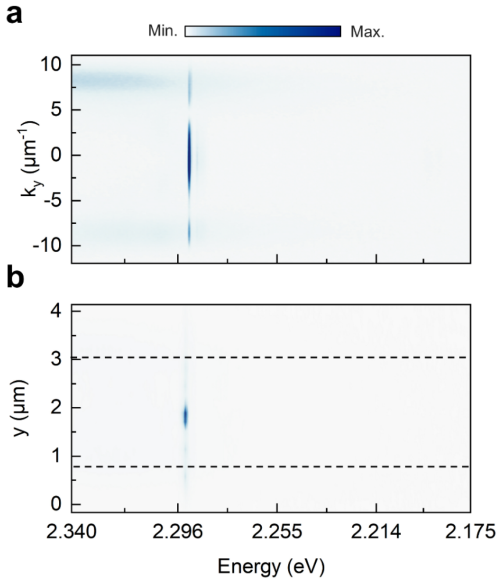

Supplementary Fig. 14| Comparison of the parametric emission in (a) momentum and (b) coordinate space, above nonlinear parametric scattering threshold. Black dashed lines in (b) highlight the real space edge of the sample.

Supplementary Note 11. Parametric scattering in a weakly confined microcavity

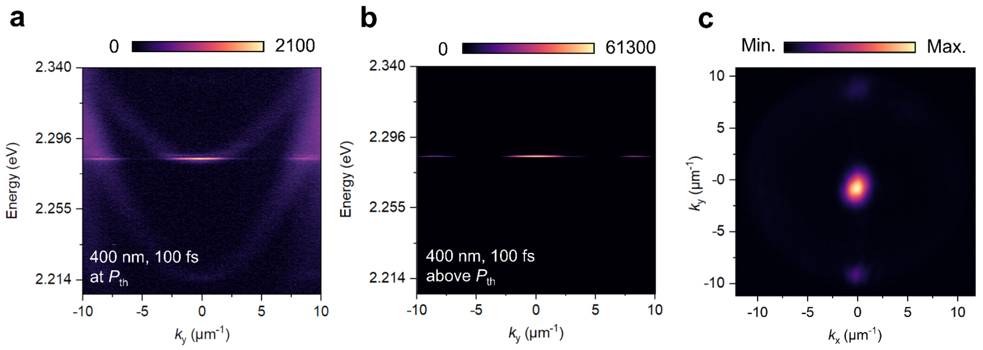

Supplementary Fig. 15| (a) Parametric scattering in a weakly confined microcavity at threshold. (b) Parametric scattering in a weakly confined microcavity above threshold. (c) *k*-space image of for (b), showing bright condensate emission and weak parametric emission.

Supplementary Note 12. Fourier space interferometry patterns of parametric polaritons in strongly and weakly confined microcavity

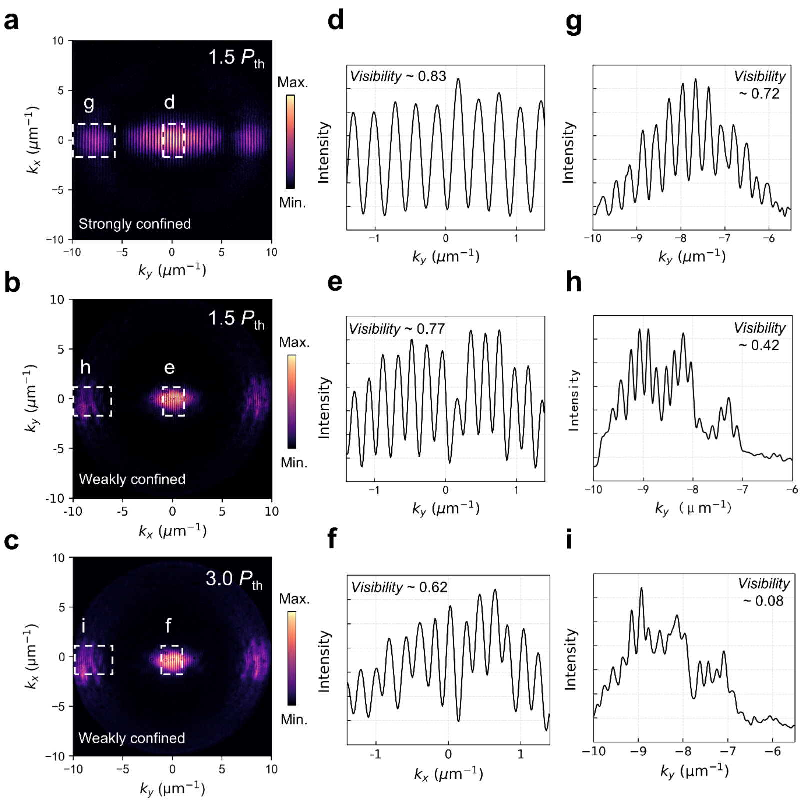

Supplementary Fig. 16| (a) Interferometry patterns of parametric polaritons in a strongly confined microcavity at 1.5 P_{th} (condensation threshold). (b) Interferometry patterns of parametric polaritons in a weakly confined microcavity at 1.5 P_{th}. (c) Interferometry patterns of parametric polaritons in a strongly confined microcavity at 3.0 P_{th}. (d-f) Respective intensity profiles extracted from the interferometric images circled by the white dashed line highlighted 'source' condensate area. d: strongly confined, 1.5 P_{th}. e: weakly confined, 1.5 P_{th}. f: weakly confined, 3.0 P_{th}. (g-i) Respective intensity profiles extracted from left panel interferometric images circled by the white dashed line highlighted 'signal + idler' area. g: strongly confined, 1.5 P_{th}. h: weakly confined, 1.5 P_{th}. i: weakly confined, 3.0 P_{th}..

Supplementary Note 13. Role of disorder in enhancing parametric emission

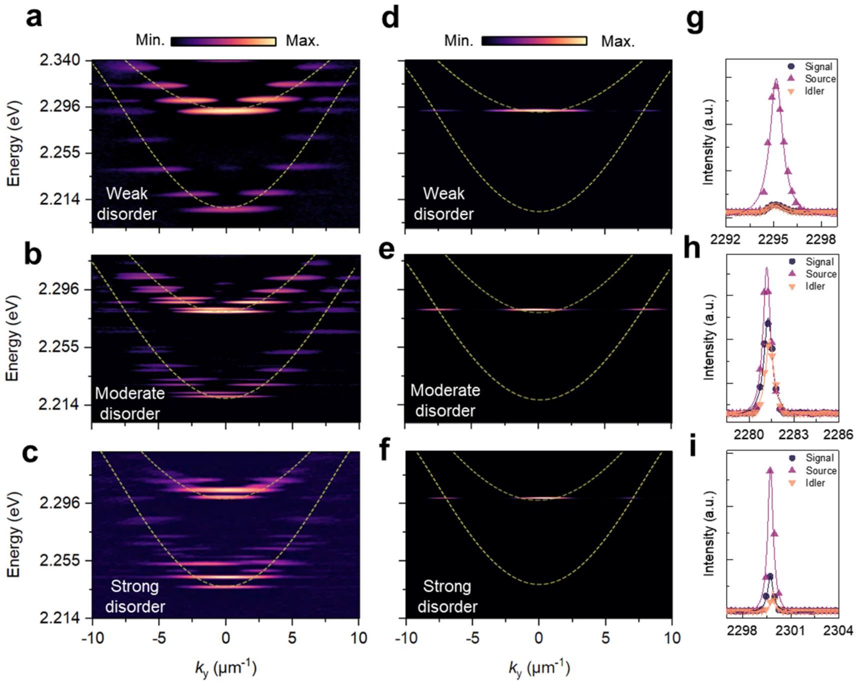

Supplementary Fig. 17| (a-c) Angle-resolved PL spectra (TM polarized) of phase matched microcavities at low excitation density. a: weak disorder; b: moderate disorder; c: strong disorder. Dashed lines are guidance for LPB1 and LPB2 dispersion. (d-f) Respective angle-resolved PL spectra of phase matched microcavities above nonlinear parametric scattering threshold. d: weak disorder; e: moderate disorder; f: strong disorder. (g-i) Spectra extracted (as discussed in Fig. S9) for respective signal, idler and source emission. g: weak disorder; h: moderate disorder; f: strong disorder.

To illustrate the role of disorder in giving rise to enhanced parametric emission, we locate three samples with close parameters including L_y (3.4 μm, 3.8 μm, 3.8 μm, respectively for a, b, c), detuning energy, coupling strength, and satisfied phase matching condition, ensuring that the variation in parametric ratio is mostly affected by levels of surface disorder. CW excitation (405 nm) was utilized to enhance clarity for the quantized energy levels in the linear regime (a-c), while pulsed excitation (400 nm) was applied to resolve nonlinear parametric emission above threshold (d-f).

In the weak disorder or surface-smooth case (Supplementary Fig. 17a), regularly spaced modes with symmetric angular dispersion are resolved in the linear regime, indicating the only presence of geometric confinement. In the moderate-disorder case (Supplementary Fig. 17b), two manifolds of mode mainly dominate: the larger spacing

for the sets of modes is related to geometric confinement, and the two sets of fine modes indicate the presence of disorder. In the case of strong disorder (Supplementary Fig. 17c), multiple disorder energy levels are superposed with the geometric confinement induced energy levels and become highly quantized and hybridized.

For the parametric emission (nonlinear regime), weak intensity is observed in the weak disorder case (Supplementary Fig. 17d), and the linewidth is relatively broad. In the moderate disorder case (Supplementary Fig. 17e), strong parametric emission is observed. A further increase in disorder density (Supplementary Fig. 17f) leads to a decrease in parametric emission intensity. Respective spectra were extracted and shown in Supplementary Fig. 17g-i. These data suggest enhanced emission in presence of disorder. It qualitatively suggests the presence of moderate disorder largely enhances parametric emission.

Supplementary Note 14. Energy mismatch in the parametric oscillator

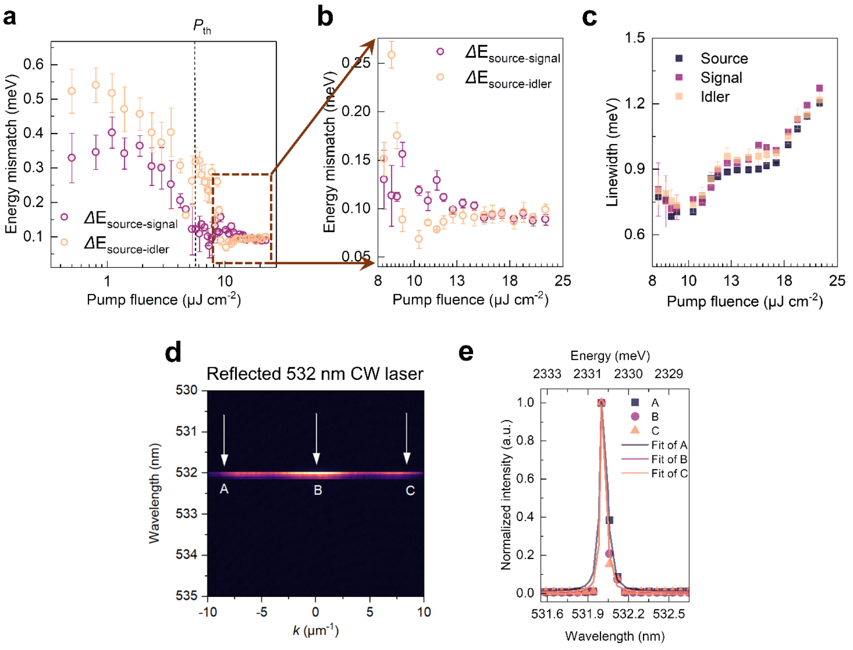

Supplementary Fig. 18| Energy mismatch analysis and exclusion of system error. (a) Evolution of source to signal, and source to idler energy mismatch in a parametric oscillator, with pump fluence from 0.3 to 27.5 μJ cm^{-2}. The dashed line indicates condensation threshold. (b) Zoom-in view of energy mismatch in dashed circled area from 8.0 to 25.0 μJ cm^{-2}. (c) Corresponding linewidth broadening for source, signal, and idler, with pump fluence from 8.0 to 25.0 μJ cm^{-2}. The source to signal, and source to idler mismatch tend to saturate, accompanied with continuous broadening in linewidth. The error bar represents the standard deviation. (d) Angle-resolved spectrum of a 532 nm CW laser. The laser was focused in real space and therefore angularly dispersed in Fourier space. (e) Normalized spectrum extracted at position A, B, C indicated by the white arrow in (d). The overlapped spectrum indicates identical dispersive feature at different positions on CCD camera, excluding the system error for the imbalanced signal, idler, and source energy position.

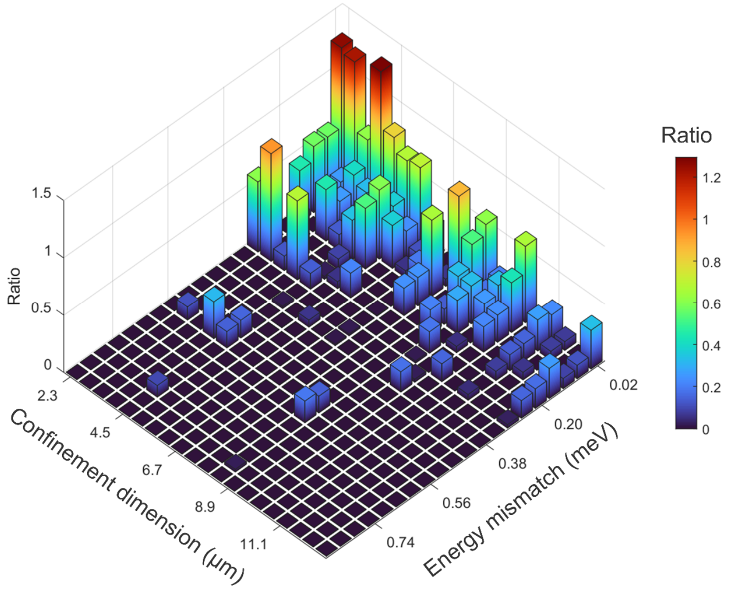

Supplementary Fig. 19| The parametric ratio in relation to both confinement dimension and LPB1/LPB2 energy mismatch. When confinement scales down and when mismatch is smaller, efficiency reaches higher values. Most of the parametric oscillators have an energy mismatch smaller than 0.35 meV. Such value is less than full width half maximum of the source and parametric emission, suggesting a quasi-phase matching condition is still needed to enable efficient parametric processes[12-14]. Such phase matching condition in confined microcavities has also been investigated by resonantly induced parametric scattering at higher energy levels within the same LPB, as reported in previous literature[15].

Supplementary Note 15. Pump-fluence-dependent emission from the microcavity in Fig. 4a.

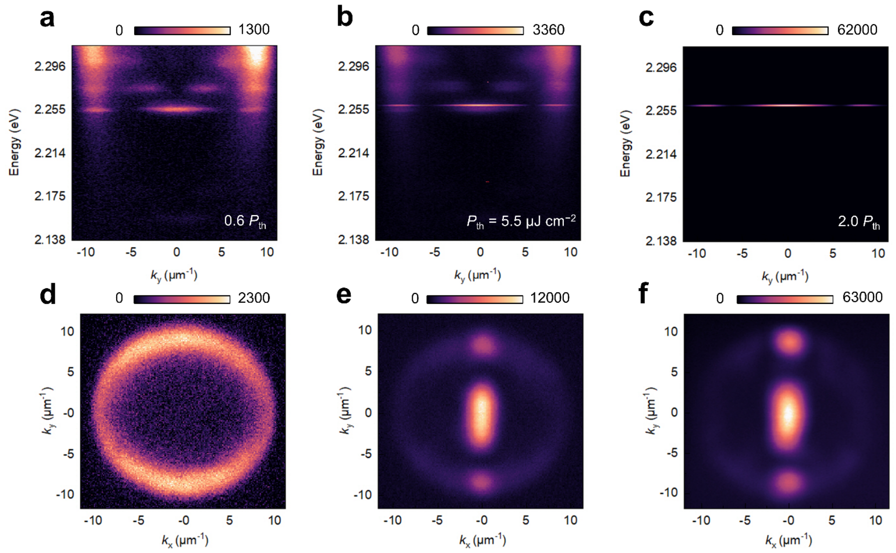

Supplementary Fig. 20| (a-c) Angle-resolved PL spectra under non-resonant pulsed excitation at $0.6P_{\mathrm{th}}$, P_{th}, and $2P_{\mathrm{th}}$, respectively. (d-f) Momentum space emission corresponding to (a-c), no additional spectral filtering was applied in collecting the momentum space emission.

Supplementary Note 16. Supersolidity in strongly confined microcavities.

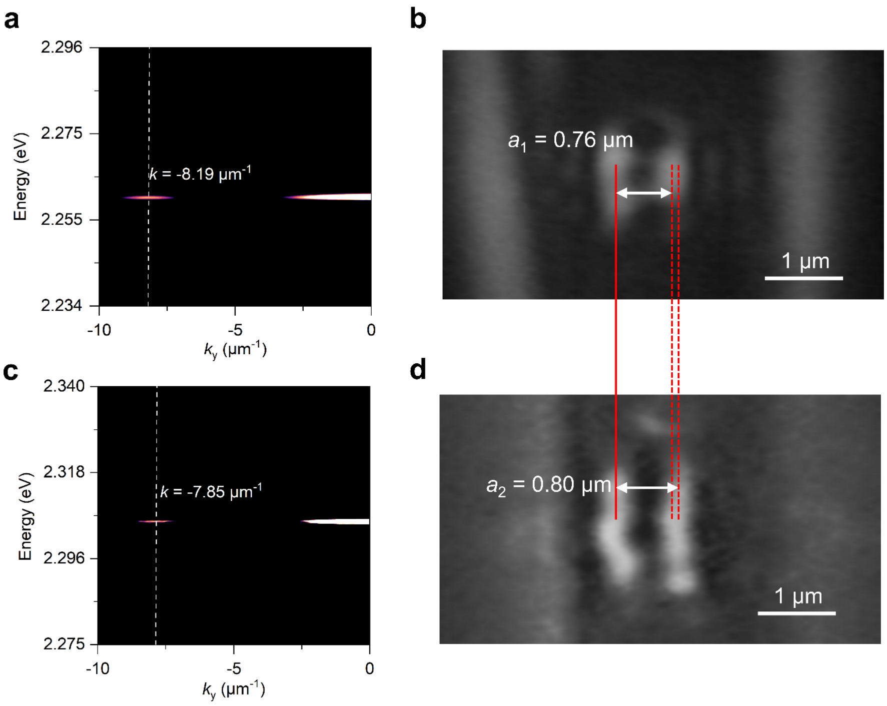

Supplementary Fig. 21| (a) Angle-resolved PL spectra of parametric oscillator with signal centered around k_1 = -8.19 μm^{-1}. (b) real space image of the parametric oscillator with fringes, showing fringe spacing a_1 = 0.76 μm, corresponding to momentum space of parametric emission in (a). (c) Angle-resolved PL spectra of parametric oscillator with signal centered around k_1 = -7.85 μm^{-1}. (d) real space image of the parametric oscillator with with fringes, showing fringe spacing a_2 = 0.80 μm, corresponding to momentum space of parametric emission in (c).

Supplementary Note 17. Theoretical models on parametric emission and supersolid phase

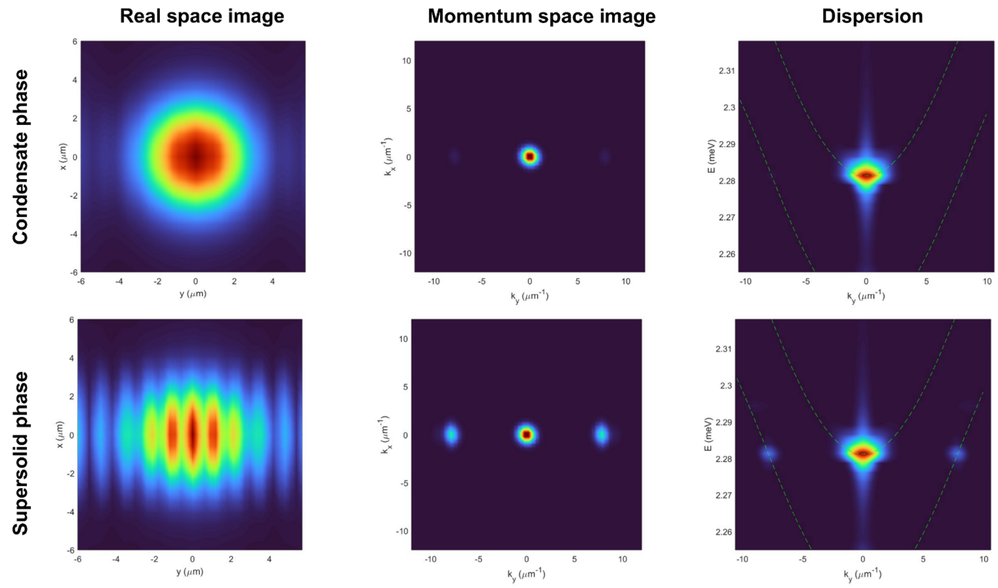

Supplementary Fig. 22| Theoretically simulated supersolid phase. Upper panels show the respective real space image, momentum space image, and dispersion of a polariton condensate. Lower panels show the respective results for a parametric oscillator, demonstrating formation of the supersolid phase.

In our theoretical model we have considered the following dispersion for the LPB:

$$E_n^{\mathrm{LP}}(k) = \frac{\left[E_n^{\mathrm{cav}}(k) + E_{\mathrm{ex}} - \sqrt{(E_{\mathrm{cav}}(k) - E_{\mathrm{ex}})^2 + \Omega^2}\right]}{2} \tag{4}$$

The dispersion relations for the pure cavity modes are given by, $E_n^{\mathrm{cav}}(k) = E_n(0) + \frac{\hbar^2}{2m_{\mathrm{cav}}}k^2$, where $E_n(0)$ is a constant energy shift which is taken different for different cavity modes. In our simulations, we have considered two cavity modes with $n = 1$ and $n = 2$. The energy separation between the modes is considered $E_2(0) - E_1(0) = 40$ meV. The effective mass of each cavity mode m_{cav} is approximately considered the same. In our simulation, we have considered the following parameters:

Rabi splitting $\Omega = 116$ meV, detuning between the cavity mode and the first cavity mode is 70 meV. System sizes are varied as mentioned in the main text. For Supplementary Fig. 22 the system size is taken as $12 \times 12\ \mu\mathrm{m}^2$. The disorder potential is considered as a random Gaussian distribution with a standard deviation $V_0 = 0.1$ meV. The effective mass of the cavity modes $m_{\mathrm{cav}} = 4 \times 10^{-5} m_e$ (where m_e is

the electron mass), nonlinear decay $\Gamma = 0.6\ \mathrm{meV/\mu m^2}$, nonlinear interaction $\alpha = 0.15\ \mathrm{meV/\mu m^2}$, and the decay rate $\gamma = 0.5\ \mathrm{meV}$. For the effective gain profile, we have considered a gaussian spot of $2.5\ \mu\mathrm{m}$ width in both x and y directions.

The emergence of spatial modulation in the supersolid phase in a coherent parametric oscillator can be approximately understood from an effective 3 mode approximation. As can be seen from the momentum space image of the supersolid phase in Supplementary Fig. 22, the effective emission is coming from the 3 modes, the condensate phase with zero momentum $\psi_c = A_c$, the signal $\psi_s = A_s\, e^{-ik_0 y}$ and the idler $\psi_i = A_i e^{ik_0 y}$. Thus, the total wavefunction is given by the superposition between all three components. However, the superposition can only be ensured by the coherence between the signal and idler, which have been proven experimentally in the main text. Given the coherence between all three components, the effective wave function is given by,

$$\psi = \psi_c + \psi_i + \psi_s \tag{5}$$

From the momentum space image, we also confirm the symmetry between the idler and the signal implying $|\psi_i| = |\psi_s|$ ensuring $|A_i| = |A_s|$. With all these, we find that the density representing the supersolid phase is given by,

$$\begin{aligned} n(r) &= |\psi(\mathbf{r})|^2 \\ &= |A_c|^2 + 4|A_s|^2 \cos^2 k_0 y \\ &+ 4\,[Re(A_s)Re(A_c) + Im(A_s)Im(A_c)] \cos k_0 y \end{aligned} \tag{6}$$

We can see the density modulation in $n(r)$ along the y axis due to the cosine function in the above expression. The density modulation comes from the coherent interference between the idler and signal creating a standing wave on top of the condensate.

In addition, in the simulation corresponding to Supplementary Fig. 22, we model the net gain for the source by setting the decay rate to $\gamma = 0.5\, meV$ and by using the following pump profile for the LPB1 mode:

$$P_1(\mathbf{r}, t) = P_0 \exp[-\frac{\mathbf{r}^2}{L^2} - \frac{t}{\tau}] \tag{7}$$

with $P_0 = 11.6\ \mathrm{meV}$, $\tau = 50\ \mathrm{ps}$, and a pump spot size $L = 11.7\ \mu\mathrm{m}$. Although the applied non-resonant pulsed pump is on the femtosecond scale, the effective temporal

profile experienced by the system is governed by the exciton reservoir. This reservoir acts as an incoherent pump with a phenomenological lifetime much longer than 100 fs, thereby setting the relevant time scale for the non-resonant pumping process. In our theoretical model, there is no linear process existing between LPB1 and LPB2.

The net gain at t = 0 for the modes in LPB1 is shown in the Supplementary Fig. 23 below and is obtained from the imaginary part of the energy eigenvalues of the corresponding Hamiltonian. As no external gain is applied to LPB2 in the model, all LPB2 modes exhibit negative net gain, reflecting losses arising solely from their finite lifetimes.

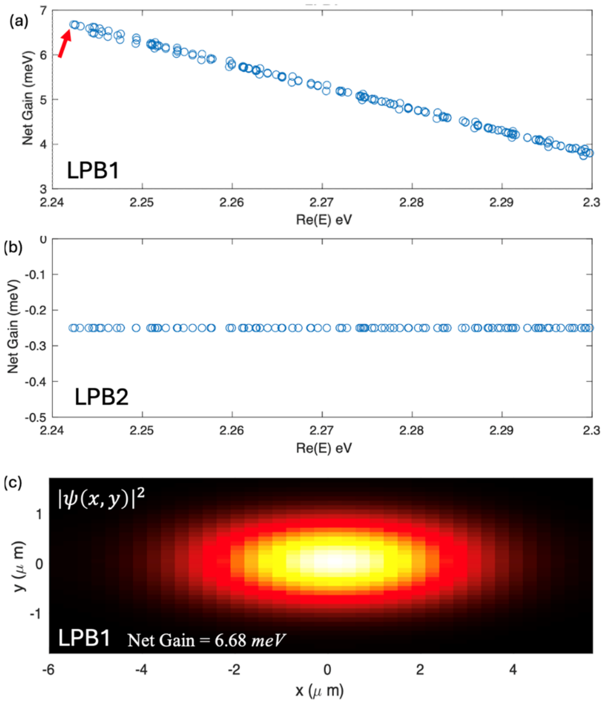

Supplementary Fig. 23| (a) The net gain of the LPB1 band, extracted from the imaginary part of the corresponding energy eigenvalues. The mode with the highest positive net gain (6.68 meV), indicated by the red arrow, identifies the state in which the source condensate forms. (b) The net gain of the LPB2 band, where all values are negative, reflecting the shorter lifetime of these modes and the absence of external pumping. (c) The spatial density profile of the LPB1 mode with the largest net gain, illustrating the real-space distribution of the condensate.

Supplementary Note 18. Theoretical simulation of resonant pump with, and without nonlinear LPB1 & LPB2 coupling.

Here, we show theoretically that the resonant excitation on the LPB1 will induce strong parametric emission only when nonlinear coupling and interaction term present for LPB1 and LPB2.

Our theoretical model is based on the following two-component driven-dissipative Gross-Pitaevskii equation, as discussed in Methods:

$$i\hbar\frac{\partial\psi_n(\mathbf{r},t)}{\partial t} = \left[\hat{E}_n^{\mathrm{LP}} + V_{\mathrm{dis}}(\mathbf{r}) - \frac{i}{2}\big(\gamma - P_n(\mathbf{r},t)\big) - i\Gamma|\psi_n(\mathbf{r},t)|^2\right]\psi_n(\mathbf{r},t) + 2\alpha\psi_m(\mathbf{r},t)^2\psi_n^*(\mathbf{r},t) + 2\alpha\psi_m(\mathbf{r},t)^2\psi_n(\mathbf{r},t) \quad (8)$$

where $n = 1,2$ represent the index of the different LPBs (here we considered two such branches, Supplementary Fig. 24). In our theoretical model, we have explicitly considered that the gain function of the second branch $P_2(\mathbf{r},t) = 0$. We pump only the first mode with a Gaussian profile $P_1(\mathbf{r},t) = P_0\exp[-\frac{\mathbf{r}^2}{L^2} - \frac{t}{\tau}]$. This choice of the pump scheme enforces that there is no gain in the second branch except the polariton transfer from the first branch to second one through the nonlinear parametric process. We have confirmed this by switching off the nonlinear term in the driven-dissipative Gross-Pitaevskii equation. We find that for a linear regime second branch shows no polariton population.

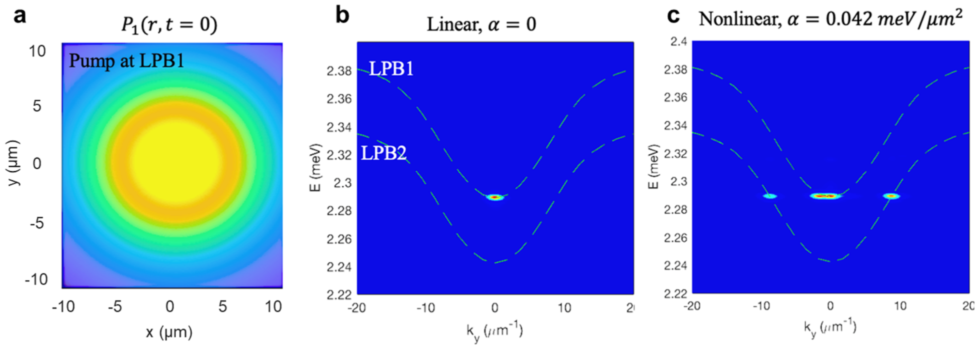

Supplementary Fig. 24| (a) gain profile of LPB1 $P_1(\mathbf{r},t=0)$, while the gain profile of LPB2 is considered zero $P_2(\mathbf{r},t) = 0$. (b) Dispersion obtained without the nonlinear terms ($\alpha = 0$). As we can see, in the absence of the nonlinear term, no effective gain exists in the second branch. (c) Dispersion obtained with the nonlinear terms ($\alpha \neq 0$) causing the parametric scattering. Here the system size is 3.6 μm, and OPO ratio ~0.82.

References:

1 Shang, Q. *et al.* Role of the Exciton–Polariton in a Continuous-Wave Optically Pumped $CsPbBr_3$ Perovskite Laser. *Nano Lett.* **20**, 6636-6643 (2020).

2 Zhou, Y. Y., Sternlicht, H. & Padture, N. P. Transmission Electron Microscopy of Halide Perovskite Materials and Devices. *Joule* **3**, 641-661 (2019).

3 Song, J. *et al.* Room-temperature continuous-wave pumped exciton polariton condensation in a perovskite microcavity. *Sci. Adv.* **11**, eadr1652 (2025).

4 Antón, C. *et al.* Optical control of spin textures in quasi-one-dimensional polariton condensates. *Phys. Rev. B* **91**, 075305 (2015).

5 Kaitouni, R. I. *et al.* Engineering the spatial confinement of exciton polaritons in semiconductors. *Phys. Rev. B* **74**, 155311 (2006).

6 Deng, H., Haug, H. & Yamamoto, Y. Exciton-polariton Bose-Einstein condensation. *Rev. Mod. Phys.* **82**, 1489-1537 (2010).

7 Yeh, P. *Optical waves in layered media*. Wiley, (1988).

8 Ermolaev, G. *et al.* Giant and Tunable Excitonic Optical Anisotropy in Single-Crystal Halide Perovskites. *Nano Lett.* **23**, 2570-2577 (2023).

9 Amo, A. *et al.* Collective fluid dynamics of a polariton condensate in a semiconductor microcavity. *Nature* **457**, 291-295 (2009).

10 Zhao, J. *et al.* Nonlinear polariton parametric emission in an atomically thin semiconductor based microcavity. *Nat. Nanotechnol.* **17**, 396-402 (2022).

11 Cilibrizzi, P. *et al.* Self-Induced Valley Bosonic Stimulation of Exciton Polaritons in a Monolayer Semiconductor. *Phys. Rev. Lett.* **130**, 036902 (2023).

12 Boyd, R. W. *Nonlinear optics*. 4. edn, Academic Press, (2019).

13 Savvidis, P. G. *et al.* Angle-resonant stimulated polariton amplifier. *Phys. Rev. Lett.* **84**, 1547-1550 (2000).

14 Saba, M. *et al.* High-temperature ultrafast polariton parametric amplification in semiconductor microcavities. *Nature* **414**, 731-735 (2001).

15 Lecomte, T. *et al.* Optical parametric oscillation in one-dimensional microcavities. *Phys. Rev. B* **87**, 155302 (2013).
