# Supplementary material for: Harnessing highly efficient coherent polariton parametric emission in quantum confined perovskite microcavities
Source: Nat Commun. 2026 Apr 2;17:5294. doi: 10.1038/s41467-026-71322-1 (PMC13270094; doi:10.1038/s41467-026-71322-1)
Supplement: Supplementary file 2 — Transparent Peer Review file [file 41467_2026_71322_MOESM2_ESM.pdf]

nature portfolio

Peer Review File

Harnessing highly efficient coherent polariton parametric emission in quantum confined perovskite microcavities

Corresponding Author: Professor Qing Zhang

This file contains all reviewer reports in order by version, followed by all author rebuttals in order by version.

Version 0:

Reviewer comments:

Reviewer #1

(Remarks to the Author)
The manuscript by Deng et al. reports efficient parametric emission from $CsPbBr_3$ microcavities while preserving a sub 1meV linewidth. The authors claim that the mechanism enabling the highly efficient generation of parametric emission is modulated by quantum confinement. Furthermore, the authors observe spontaneous 'crystallization' at room temperature forming a supersolid phase with real space periodicity. The work is relevant to the community, the efficient generation of narrow linewidth parametric emission is a remarkable outcome. However, a deeper analysis of the mechanism enabling this process is essential to make this a scalable process.

Main Comments:
1) The manuscript attributes the discretization of the polariton dispersion to a combination of geometric confinement and disordered surface potentials, with a reported 7.5 meV mode spacing. However, the discussion does not clearly distinguish the contributions from in-plane confinement and disorder. A systematic study isolating the effects of in-plane confinement, and surface roughness, such as comparing dispersions for different lateral sizes and controlled disorder amplitudes, would strengthen the mechanistic interpretation and clarify the origin of the observed discretization. From Fig. 1b, the roughness reaches ~10 nm, an analysis showing that this amplitude can localize lower polaritons and yield a level spacing on the order of the observed, would be beneficial. To separate the two contributions, k-space reflectance for similar cavities with reduced surface roughness would explain how the spacing and the appearance of discrete lines evolve relative to surface roughness.

2) The term "non-confined cavity" needs an explicit definition. A more detailed explanation of what this means would benefit the paper. Does this denote a planar microcavity with a larger crystal? The authors should report the fabrication process for this larger device. Providing these details will allow for a fair comparison with the confined structures.

3) The authors claim that the disordered surface potentials that are formed as a result of the fabrication process are instrumental in generating fine modes and efficient parametric emission. However, the role of the disorder in enhancing parametric emission if not quantitively measured. A study of how the different levels of surface roughness effect the signal /ideler ratio would be beneficial.

4) The authors chose $CsPbBr_3$ as the active material, but the manuscript does not justify why this particular halide perovskite is uniquely suited for sustaining parametric scattering and supersolid behavior. Could other halide compositions (e.g., $CsPbI_3$, mixed halides, or hybrid organic-inorganics) offer advantages in terms of exciton binding energy, linewidth, or tunability of the cavity modes? A few sentences in the introduction would help here.

5) The manuscript would benefit from structural characterization of the $CsPbBr_3$ microplatelets. While the microcavities are strongly characterized, no direct evidence is provided for the crystallographic quality or phase purity of the material. Techniques such as X-ray diffraction could confirm the absence of secondary phases, strain, or structural defects that might influence the dispersion and parametric scattering behavior. Including such data would strengthen the link between the observed effects in the strongly coupled system and the intrinsic properties of the active material, and ensure that the reported effects are not influenced by variations in material quality.

6) Given that perovskites are prone to point defects and surface traps, how do the authors ensure that these do not dominate the coherence and linewidth of the parametric emission? Is there any passivation strategy or post-treatment applied or can you comments on how that might affect properties?

Reviewer #2

(Remarks to the Author)
The manuscript "Harnessing highly efficient coherent polariton parametric emission in quantum confined perovskite microcavities" by Xinyi Deng et al. reports the first-time observation of resonantly enhanced parametric scattering of exciton-polaritons in a halide perovskite microcavity. The authors emphasize the key result of their work: the strong influence of in-plane polariton quantization, accompanied by potential disorder, on the nonlinear optical response of the studied system. They experimentally observe coherent polariton emission involving parametric scattering and also claim the "first observation of strong phase coherence between the two parametric species in momentum space."

The importance of the observations is not in doubt: the suggested arrangement is suitable for generating phase-correlated photon pairs, with potential for operation down to the single-photon regime. The halide perovskite platform is favorable in this sense, as it is known for its ultimate quantum efficiency of exciton-enabled light emission.

The manuscript is generally well-written. However, some further clarifications and polishing of the text would be beneficial, as noted below. The team employs well-established experimental methods; therefore, the data quality is suitable. The analytical model is clear; some parameters used need to be specified in the updated version of the manuscript.

Although I am generally positive about this work, I must point out that some of the authors' claims require further justification. The authors have to address several questions before I can recommend this work for publication in Nature Communications.

Major issue:

My primary concern is related to the mechanisms of population of signal and idler states. I can suggest at least three of those:
(1) Conventional relaxation from the reservoir to a resonant state (gain).
(2) Linear scattering of polaritons from the source to signal and idler states.
(3) Nonlinear parametric scattering of source polaritons towards signal/idler state, claimed as the primary mechanism.

Firstly, the right panel in Figure 2a clearly shows the strong asymmetry in signal (1.36)/idler (0.43) relative intensities. In case of mechanism (3), exclusively responsible signal/idler population, this picture must be purely symmetric. The largest of these two values is compared to other works in Figure 2b and is referred to as "parametric emission efficiency", which might mislead the reader. This efficiency is fundamentally limited by the minimum of the two values mentioned for each particular measurement.

The authors state in the text: "Below the first threshold, incoherent relaxation from the exciton reservoir is responsible for the population at signal and idler states." It is unclear why authors neglect this mechanism when discussing the system behavior above the first thresholds.

Let me suggest an explanation of the observed effects involving only mechanisms (1) and (2). Above the first threshold, the condensate appears at the source state. The polaritons there undergo elastic scattering into signal/idler modes, with efficiency enhanced by surface disorder. These scattered polaritons seed the condensates in signal/idler states, which appear at a larger threshold and remain coherent with the source, as they are seeded by polaritons coming from it. Another reason for the signal, source, and idler states to be mutually coherent is the complete overlap of these modes in real space.

The condensation threshold for the signal/idler states can be larger than that of the source state for several reasons, including differences in polariton lifetimes. The nearest polaritonic state below the source is more than 50 meV apart, while for signal/idler states, this distance is below 10 meV.

I firmly believe that with this massive amount of experimental data in hand, authors can distinguish between mechanisms (1-3) more accurately.

Another way to completely exclude the mechanism (1) from the list is to perform additional measurements with the resonant pumping of the source state by appropriately tuning the excitation photon energy and adjusting the spot size to avoid direct excitation of signal/indler modes. To distinguish pump photons from those in a condensate (and estimate the condensate emission rate), one can pump at an angle, staying within the positive/negative in-plane wavevectors, yet remaining resonant with the source mode.

Additional related question: What are the values of net gains P_n for signal, source, and idler used for the modelling in Figure S11?

Minor remarks:

1. In the manuscript, only the elastic scattering process is discussed. Meanwhile, Figure 2c reveals an apparent spectral mismatch between signal, source, and indler emission peaks. Owing to the inversion symmetry of the system, is there any explanation for this effect? Could it be a measurement artifact, such as a slight misalignment of the CCD camera, which needs to be corrected during data post-processing?

2. In the text, the authors compare confined and unconfined microcavities. Unfortunately, I was unable to find the two respective definitions. It is worth clarifying this for the reader.

3. I suggest adding a discussion on the role of the second dimension of the rectangular microcavities. Why can the confinement in the second dimension be neglected?

4. I was also unable to find an explicit discussion of the role of potential disorder. How does surface roughness improve the system's performance?

One aspect could be as follows. Polariton, scattering on surface roughness, may acquire an additional portion of momentum, inversely proportional to the lateral size of the surface feature. This process, therefore, enhances scattering from the source to signal/idler states.

5. Authors state: "Meanwhile, downscaling the confinement dimension leads to energy eigenstates with larger spacing that induces the randomness in matching of source and signal/idler energy position, leading to a larger fluctuation amplitude in parametric interaction strength and acquisition of highly efficient parametric emission in confined microcavities."

Indeed, the LA1 and LA2 branches have generally different sets of quantized levels. The detuning (and overlap) between the fundamental level of LA1 (the source state) and the nearest LA2 state is expected to significantly affect both linear and nonlinear elastic scattering processes in the system.

In this context, would the data presented in red symbols in Figure 3a, showing nearly random behavior, become better structured when used to correlate signal&idler/source with the LA1-LA2 nearest-level detuning? Of course, the randomness induced by surface roughness will stand.

6. Within the text, one can find many terms, probably referring to the exact property of the system under study: surface potential landscape, disorder potential landscape, disordered surface potential landscape, transfer-induced potential landscape, disorder potential, etc. I recommend leaving a single term.

7. The authors should proofread the Supplementary Material:
Figure S2 ("microscope" misspelled),
Figure S5 (d-f) - is it a single frequency or summation over a range?
Figure S6 (b) and (a) should be exchanged.
Figure S7 (g) instead of (b). It is unclear why the source and idler energy- and wavevector-dependent spectra are plotted on different axes. I suggest using the exact representation and adding the signal plots as well.

I would be glad to review the revised manuscript if the authors address the concerns and questions that have been raised.

Reviewer #3

(Remarks to the Author)
I co-reviewed this manuscript with one of the reviewers who provided the listed reports. This is part of the Nature Communications initiative to facilitate training in peer review and to provide appropriate recognition for Early Career Researchers who co-review manuscripts.

Reviewer #4

(Remarks to the Author)
The work by Deng et al. reports on the efficient generation of parametric emission in a polaritonic microcavity. The authors claim that the presence of random in-plane potentials is responsible for the observed anomalous enhancement of parametric scattering. To support their claims, they first present a comparison among three configurations: a non-confined microcavity (showing low-efficiency scattering), a laterally confined cavity without random potential (showing no parametric scattering), and a laterally confined cavity with random potential (exhibiting highly efficient scattering). They then perform statistical analysis to justify the observed high efficiency and develop a theoretical model. Finally, they also present evidence of supersolidity at room temperature.
The topic is certainly interesting, and the overall idea of investigating the role of disorder and lateral confinement in the regime of nonlinear polariton scattering is nice. However, I found some aspects of the work problematic and the final claims somewhat unclear. The authors attempt to highlight the role of disorder, suggesting that scattering is not observed in a similar laterally confined structure without disorder. This implies that disorder is a fundamental requirement. However, a close examination of Fig. S9 clearly shows that phase matching cannot be satisfied in that case. Therefore, the comparison is not accurate, and the authors' claim appears incorrect, especially considering that parametric scattering has been

previously observed in laterally confined systems (e.g., 10.1103/PhysRevB.87.155302). Indeed, they later acknowledge that downscaling the lateral confinement introduces randomness in the phase-matching conditions, leading to larger fluctuations in the occurrence of highly efficient parametric emission. However, it is unclear what the authors are precisely claiming here. If they intend to argue that strong and coherent parametric emission is uniquely associated with laterally confined disordered microcavities, then they should present a reference sample—free from disorder but with well-matched phase-matching conditions—for a meaningful comparison. Otherwise, the role of disorder appears to be limited to mode broadening which facilitate phase matching, which is less significant.

Even the comparison with the non-confined system is problematic. The sample shown in Fig. S8 exhibits a more positive detuning (with visible lasing around 2323 meV), while the confined-disordered cavity is more negatively detuned (~2300 meV). This results in a higher excitonic fraction for the non-confined sample, which strongly influences both the visibility of the parametric emission and, in fact, the condensate itself. The condensate at k=0 in the non-confined sample appears to have lower visibility compared to that of the confined-disordered cavity. Therefore, this comparison does not convincingly support the claims regarding coherence. Moreover, the reported efficiency value—while I appreciate the large statistical analysis and the effort put into developing a suitable model—also appears problematic. The claimed efficiency seems to exceed the theoretical limit for a parametric process, in which the combined intensity of the signal and idler cannot surpass that of the pump. This suggests that there may be an issue in how the overall intensity of the condensate is being evaluated.

Overall, although the work has potential, I cannot recommend publication of the manuscript in its current form. A clear and convincing demonstration of the key claims—particularly regarding the role of disorder and the reported efficiency—is necessary before the work can be considered for publication.

Below is a list of my comments.

-In the introduction, the authors cite the work from J. Wu et al. (10.1021/acs.nanolett.1c00283), stating that the reported case exhibits suboptimal parametric emission efficiency and that pump powers "several times" above the condensation threshold are required to observe parametric scattering. However, upon reviewing the referenced paper, it is clear that parametric scattering is already visible at pump powers 2–3 times above the condensation threshold. This would more accurately be described as "a few times," rather than "several," and does not support the implication of particularly poor efficiency.

-The authors used the same sample in one of their recent publications to observe polariton condensation under CW excitation. Why a pulsed excitation scheme was chosen for the present experiments?. Could the authors show how the excitation regime (CW vs. pulsed) influences the key observed properties—such as parametric efficiency, linewidth, and coherence? Are these parameters intrinsic to the system or strongly dependent on the excitation conditions?

-Still regarding the excitation scheme, polariton parametric scattering has been extensively studied in GaAs-based 1D microcavities, where, under resonant excitation, compelling evidence for phase matching and its critical role in the scattering process has been demonstrated (10.1103/PhysRevB.87.155302, 10.1002/pssb.201100115). Have the authors attempted similar measurements under resonant excitation? Would the reported effects—particularly the claimed high efficiency and coherence—still hold under pulser or CW resonant excitation? These experiments would help to establish the generality and robustness of the observed phenomena.

-Regarding the surface-smooth sample shown in Fig. S9, it is unclear why the discretization of the TE_1 and TM_1 modes is not visible. Given that this sample is used as a key comparison point, the absence of clear mode discretization raises questions about the confinement quality. As mentioned earlier, to convincingly support their claims about the role of disorder, the authors should identify a surface-smooth sample with good phase matching, similar lateral dimensions, and comparable detuning.

-How are the overall intensities of the signal, idler, and source evaluated? Part of the condensate signal may propagate outside the excitation region and thus be excluded from the intensity measurement. If so, this could lead to an underestimation of the total condensate power and therefore to high efficiency. Additionally, the authors should provide an estimate of the experimental uncertainty associated with the efficiency ratio evaluation.

-The authors emphasize that the presented configuration enables the generation of balanced signal and idler beams. However, in Fig. 2a, the idler appears to be approximately three times weaker than the signal. The authors should clarify the origin of the imbalance.

-For the non-confined microcavities below threshold dispersion should be shown.

Version 1:

Reviewer comments:

Reviewer #1

(Remarks to the Author)

The authors have thoroughly addressed all points raised during the first round of revisions. The revised manuscript and improved Supplementary Information will be valuable to the community, and I therefore recommend publication. One minor point remains regarding terminology. The manuscript uses the term “disorder” in a way that is ambiguous, since disorder can refer to multiple mechanisms (morphological, crystallographic, compositional, or energetic). In the present context, the effect being discussed is specifically due to surface or interface roughness. I recommend replacing “disorder” with something more specific, like roughness. This will make it more clear for the reader that the disorder being discussed here is morphological.

Reviewer #2

(Remarks to the Author)

First, I appreciate the authors’ impressive efforts, including additional experiments, which enabled them to address the

concerns raised by the reviewers in the previous stage. In particular, in the refactored text, the authors address previously missing critical points: explicitly define terms, identify the dominant parametric scattering mechanism of the observed coherent emission, and distinguish the roles of confinement and roughness in achieving high emission efficiency.

After carefully reading the updated text, I have two minor suggestions to further improve the paper's clarity:
1. The authors define a specific threshold value (4 μm) for confinement, which is likely structure-specific. To be more general and material-platform-independent, one could define a strongly confined system as one capable of supporting a parametric ratio greater than 1.
2. It is worth specifying the sizes of the pump spot and the spatially filtered detection region, since both may affect the observations. Accordingly, a long focal distance lens may be missing in the excitation channel in Fig.S6.

These cosmetic remarks do not require further review and do not affect the novelty, scientific value, results, or conclusions of the work.

The authors' claims and conclusions are now well supported by the data and analysis presented in the manuscript. In its current form, the work constitutes a valuable contribution to nonlinear polaritonics. I therefore strongly support its publication in Nature Communications.

Sincerely,
Anton Samusev

Reviewer #3

(Remarks to the Author)
I co-reviewed this manuscript with one of the reviewers who provided the listed reports. This is part of the Nature Communications initiative to facilitate training in peer review and to provide appropriate recognition for Early Career Researchers who co-review manuscripts.

Reviewer #4

(Remarks to the Author)
The authors have carefully addressed the concerns raised in my previous report and have substantially strengthened the manuscript through additional experiments and a clearer discussion. In particular, the role of disorder is now supported by more appropriate reference samples with comparable confinement, detuning, and phase-matching conditions, and the comparison between strongly confined and weakly confined microcavities is significantly improved. The clarification regarding the definition of the parametric ratio and the distinction from pump efficiency also resolves the main conceptual ambiguity.
In addition, I note that further measurements requested by other referees (such as resonant excitation experiments) have been successfully implemented and add important information. At this stage, I believe that only one additional clarification would further consolidate the physical picture. Since the manuscript now explicitly discusses TE and TM mode quantization and presents their respective dispersions, as requested in the previous review, it would be highly valuable to demonstrate whether parametric scattering under resonant excitation can be achieved by pumping a discretized localized mode rather than the ground state where condensation occurs.
Specifically, with reference to Fig. R18, it would be important to clarify whether parametric scattering can be realized within the same polariton branch by resonantly exciting one of the higher-energy localized states at zero in-plane momentum. As phase matching for these higher-energy states is satisfied in both LPB2 and LPB3, as well as in both TE and TM polarizations, demonstrating parametric scattering under these conditions would provide a stringent test of the proposed mechanism.
Apart from this point, the main scientific issues raised in the previous review have been addressed, and the manuscript is close to being suitable for publication.

Open Access This Peer Review File is licensed under a Creative Commons Attribution 4.0 International License, which permits use, sharing, adaptation, distribution and reproduction in any medium or format, as long as you give appropriate credit to the original author(s) and the source, provide a link to the Creative Commons license, and indicate if changes were made.
In cases where reviewers are anonymous, credit should be given to 'Anonymous Referee' and the source.
The images or other third party material in this Peer Review File are included in the article's Creative Commons license, unless indicated otherwise in a credit line to the material. If material is not included in the article's Creative Commons license and your intended use is not permitted by statutory regulation or exceeds the permitted use, you will need to obtain permission directly from the copyright holder.
To view a copy of this license, visit https://creativecommons.org/licenses/by/4.0/

Response to Reviews (NCOMMS-25-52756-A)

Response to Reviewer 1

The manuscript by Deng et al. reports efficient parametric emission from $CsPbBr_3$ microcavities while preserving a sub-1-meV linewidth. The authors claim that the mechanism enabling the highly efficient generation of parametric emission is modulated by quantum confinement. Furthermore, the authors observe spontaneous 'crystallization' at room temperature, forming a supersolid phase with real space periodicity. The work is relevant to the community, the efficient generation of narrow linewidth parametric emission is a remarkable outcome. However, a deeper analysis of the mechanism enabling this process is essential to make this a scalable process.

Response:

We thank the reviewer for the careful evaluation of our manuscript. We are grateful for the positive comments on the novelty of our work and the on the significance of efficient parametric emission with coherent features. We also appreciate the constructive suggestions on clearer identification of energy levels, and illustration of disorder in enhancing parametric emission, which have helped us in a clearer demonstration of the effect of the geometric features. Following these recommendations, we have added additional experimental results together with extended analysis and discussions, which we believe could address the reviewer's concerns. The point-by-point responses are highlighted in blue color, and the corresponding modifications in the main text are marked in blue color.

Comment 1:

The manuscript attributes the discretization of the polariton dispersion to a combination of geometric confinement and disordered surface potentials, with a reported 7.5 meV mode spacing. However, the discussion does not clearly distinguish the contributions from lateral confinement and disorder. A systematic study isolating the effects of lateral confinement, and surface roughness, such as comparing dispersions for different lateral sizes and controlled disorder amplitudes, would strengthen the mechanistic

interpretation and clarify the origin of the observed discretization. From Fig. 1b, the roughness reaches ~10 nm, an analysis showing that this amplitude can localize lower polaritons and yield a level spacing on the order of the observed, would be beneficial. To separate the two contributions, k-space reflectance for similar cavities with reduced surface roughness would explain how the spacing and the appearance of discrete lines evolve relative to surface roughness.

Response 1:

We thank the reviewers for the valuable suggestion on assigning energy levels in Fig. 1 more clearly to confinement and disorder, which helps us illustrate the role of 'confinement' and 'disorder' in the dispersion more explicitly. And we thank the reviewer for the helpful suggestions on possible methods to identify the energy levels. Here in the revised manuscript, we employed two approaches to isolate the respective energy levels and revised the respective Fig. 1 dispersion.

As the reviewer suggested, precisely controlling the disorder amplitude would be very valuable; however, this remains extremely challenging at present. Given the constraints of typical top-down fabrication methods (*Nat. Commun.* **15**, 3345 (2024), *Science* **367**, 1018–1021 (2020)), it is difficult to reliably achieve the required dimensional profile showing an lateral size ranging from several tens to several hundred nanometers, combined with a thickness of 5–15 nm. Thus, herein we employed spatial selection of the PL from different sites showing different degrees of disorder in a microcavity sample (L_y = 3.7 μm, L_x = 12.6 μm, Fig. R1a). As shown in Fig. R1b, the TM polarized angle-resolved PL spectrum at location-1 (left panel, weak or nearly without disorder) reveals the confinement induced energy level quantization on the two lower polariton branches (LPBs), with regularly spaced energy levels (highlighted with gray dashed lines) due to geometric confinement (*Phys. Rev. B* **74**, 155311 (2006)), *Phys. Rev. B* **91**, 075305 (2015)). While in the angle-resolved PL spectrum for the location-2 (disordered area, right panel), more quantized energy levels appear in addition to the confinement induced energy levels, suggesting the role of disorder. Moreover, in presence of disorder, the overall linewidth for the LPB modes becomes narrower, indicating the effect of moderate level of disorders in generation of extra fine modes as we studied before (*Sci. Adv.* **11**, eadr1652 (2025)).

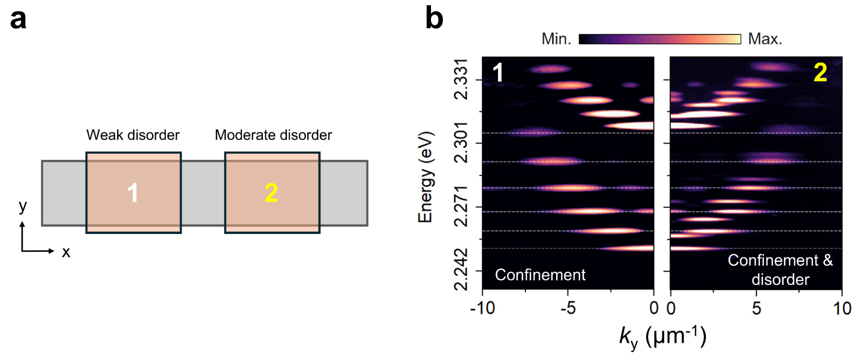

Fig. R1. (a) Schematic for collection sites from a confined microcavity, with weak surface disorder (1) and moderate surface disorder (2). (b) Angle-resolved PL spectra (TM polarized) from the sites in (a), where region (1) emission is mainly from confinement, and region (2) emission stems from both confinement and disorder. Gray dashed lines highlight the energy levels on this LPB associated with confinement in *y* direction.

In a confined microcavity with micron-scale size, the energy of the polariton could be written as (*Phys. Rev. B* **74**, 155311 (2006), *Phys. Rev. B* **91**, 075305 (2015))

$$E_{n_x,n_y} = \frac{\hbar^2\pi^2}{2m^*}\left(\frac{n_x^2}{L_x^2}+\frac{n_y^2}{L_y^2}\right)+V_0$$

where E is the energy of LPB, m^* as the effective polariton mass, $n_{x,y}$ as the quantum number for quantized lower polariton branches, $L_{x,y}$ as the confinement size for x and y directions, V_0 is the potential depth. In our $CsPbBr_3$ MPs, $L_x >> L_y$, as we later discuss in Response 2, the x-direction behavior could be treated as a free particle with continuous momentum k_x while the y-direction experiences confinement and energy level quantization. The energy difference between adjacent states becomes

$$\Delta E = \frac{\hbar^2\pi^2}{2m^*L_y^2}\left(2n_y+1\right)$$

For the lowest two energy levels ($n = 0$ and $n = 1$), the relationship becomes

$$\Delta E \approx \frac{3\hbar^2\pi^2}{2m^*L_y^2}$$

In the microcavity as shown in Fig. R1b, the polaritons for LPB2 and LPB3 have effective mass (in the limit of small wave vectors, *Rev. Mod. Phys.* **82**, 1489–1537

(2010)) of 1.404×10^{-5} m_e and 1.160×10^{-5} m_e, respectively, and the calculated lowest energy spacing for the two branches are 5.75 meV and 6.96 meV, close to the measured mode spacing of 6.0 meV and 7.2 meV, as shown in Fig. S7 and discussions. Therefore, such derivation could resolve quantized energy levels arising from geometric confinement.

For a one-to-one correspondent characterization of AFM profile and polariton dispersion, we have demonstrated previously in *Sci. Adv.* **11**, eadr1652 (2025). To illustrate the effect of 10 nm variation as raised by the reviewer, we conducted the transfer matrix simulation (*Optical Waves in Layered Media*. Wiley, 1990, *Phys. Rev. B* **74**, 155311 (2006)) of the cavity transmission with 10 nm perturbation in a thick perovskite cavity. As an example, the calculation shows that for a cavity with perovskite thickness of 2.0 µm, one of the resonant modes appear at 2305.10 meV. When the surface 10 nm of the perovskite layer is replaced with PMMA (refractive index: 1.45), the mode positions shift to 2311.30 meV, giving an energy shift of 6.20 meV in LPB bottom. The newly emerged lowest energy level as shown in Fig. R1b right panel lies 5.46 meV above the original LPB bottom energy level, reflecting a disorder with ~8 nm thickness, comparable with the disorder profile we depicted in Fig. 1. In addition, when multiple disorders coexist, their corresponding energy levels undergo further hybridization, giving rise to more intricate dispersion profiles. This phenomenon has been established in our prior work and will not be revisited here.

Revision 1:

Main text, page 4, Fig. 1b and Fig. 1d revised.

Main text, page 3, added: 'To illustrate the respective roles of confinement and disorder on polariton dispersion, TM polarized PL spectra was collected from this microcavity on sites (Fig. S8) with weak disorder (Fig. 1d, left panel) and moderate disorder (Fig. 1d, right panel). The spacing for lowest two energy levels are 6.0 meV and 7.2 meV respectively for the two LPBs depicted on left panel, consistent with the model considering L_y dimension and polariton effective mass in the small wavevector limit (Fig. S7)[1]. For the moderate disorder regime, additional energy levels arise with an overall narrowed linewidth, making the dispersion further enriched to fulfill phase

matching condition and to enhance polariton parametric interactions through lifted degeneracy[2-6]. Here the lowest energy associated with disorder lies 5.75 meV above the lowest energy level, reflecting an effective disorder height of ~ 8 nm (Fig. S8)[7].'

Main text, page 4, Fig 1d, caption, addd: '(d) Transverse magnetic (TM) polarized angle-resolved photoluminescence (PL) spectra from a microcavity with L_y = 3.7 μm (excitation laser: CW, 405 nm). Left panel: weak disorder, predominated by L_y confinement; right panel: moderate disorder, affected by both L_y confinement and disorder potential (Fig. S8).'

SI, page 7-9, added: Fig. S7 and Fig. S8, and discussion on the evaluation of energy levels associated with confinement and disorder.

Comment 2:

The term "non-confined cavity" needs an explicit definition. A more detailed explanation of what this means would benefit the paper. Does this denote a planar microcavity with a larger crystal? The authors should report the fabrication process for this larger device. Providing these details will allow for a fair comparison with the confined structures.

Response 2:

We thank the reviewers for pointing out the issue for a clear definition of confinement and the fabrication process. We fabricated the single-crystalline $CsPbBr_3$ MPs using the anti-solvent synthesis route, as shown in Fig. R2, as we reported in previous studies (*Nano Lett.* **20**, 6636–6643 (2020)). A glass substrate was placed in a petri-dish in an anti-solvent atmosphere. The beaker was filled with diluted anti-solvent and sealed with perforated Parafilm which enabled gradual evaporation and crystallization in the precursor solution. Such process yields $CsPbBr_3$ MPs with various lateral dimensions for the study in this work (Fig. 2b). Notably, neither external spatial confinement nor pressure was applied during growth, which ensures uniform surface roughness and crystallinity quality for the larger and smaller MPs. The as-grown MPs were then transferred to bottom DBR and spin coated with PMMA and finally encapsulated with top evaporated DBR mirrors. Fig. R2c and R2d show the AFM images and the time-

resolved photoluminescence (TRPL) spectra on the MPs (before transfer process) with varying lateral dimensions, respectively. The surface roughness is almost the same. Meanwhile, TRPL suggests identical bi-exponential decay curves with comparable fast and slow decay lifetimes, indicating similar defect densities and carrier recombination behavior (*Nat. Photonics* **10**, 295–302 (2016)).

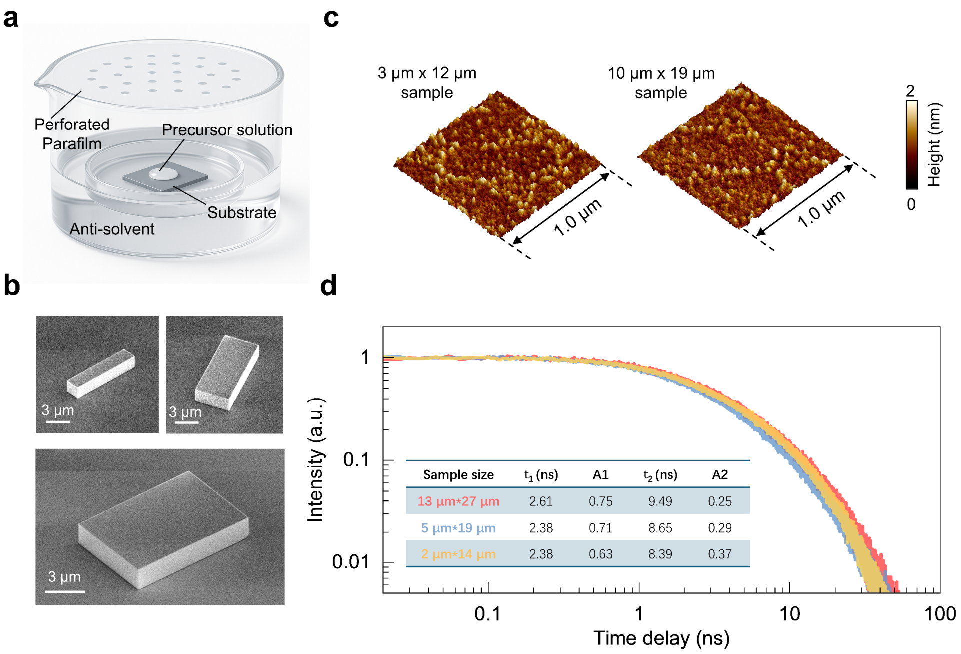

Fig. R2. Fabrication and characterization for the $CsPbBr_3$ MPs. (a) Schematic of anti-solvent synthesis procedure. (b) SEM images for tilted view of $CsPbBr_3$ MPs before PDMS transfer. (c) AFM measurement of surface roughness profiles for the MPs with different lateral dimensions. (d) The time-resolved photoluminescence for the MPs at pump fluence of 1 $\mu J \cdot cm^{-2}$. Excitation laser: 515 nm, 50 kHz, 250 fs.

Next, we would like to explain the 'confinement' regime according to your suggestion. The MPs typically exhibit an anisotropic lateral dimension with L_x (length of x-axis) > L_y (length of y-axis). The L_x is generally no less than 10 µm, while L_y can be as short as around 2 µm (Fig. S2). While polariton thermal de Broglie wavelength ($\lambda_{dB} = \sqrt{2\pi\hbar^2 (m_{\mathrm{eff}} k_B T)^{-1}}$, *Phys. Rev. Lett.* **121**, 067401 (2018)) is 1.1-1.3 µm. Therefore, the confinement effect is considered according to L_y dimension. According to the model of quantum size effect as displayed in Fig. 3, an evident increase in parametric emission ratio emerges at L_y ~4 µm, which we use as the 'boundary' for different levels of confinement. To make the statement clear, according to your

suggestion, we revise the 'confined' and 'non-confined' terminology to '*strongly confined*' and '*weakly confined*', referring respectively to samples with L_y *smaller than* or *larger than* 4 μm.

Revision 2:

Main text, page 3, added: 'The MPs were fabricated by an anti-solvent route (Fig. 1b)[8], which exhibit an anisotropic lateral dimension with L_x (length of *x*-axis) > L_y (length of *y*-axis). The L_x is generally no less than 10 μm, while L_y can be as short as around 2 μm (Fig. S2). Nevertheless, the MPs feature smooth facets and exhibit consistent optical properties across the relevant range of lateral dimensions (Fig. S2, S3). Considering the polariton thermal de Broglie wavelength (1.1-1.3 μm, Methods)[9], the influence of L_x could be neglected, and we therefore consider the spatial confinement and energy quantization according to L_y dimension[7]. Since the high-efficiency parametric emission emerges when L_y decreases to 4 μm as shown in later discussions, we define the regime where L_y is 'smaller than' 4 μm as 'strongly confined' and L_y is 'larger than' 4 μm as 'weakly confined', respectively.'

Main text, page 4, the caption of Fig.1, added: 'Inset shows cross-sectional view of the MP typical anisotropic dimension with *y* direction dimension (L_y) approximately 2-4 μm, and the thickness typically ranging from 1 to 3 μm. Surface disorders are formed by a stamp transfer process. (b) Scanning electron microscope (SEM) image of an anti-solvent grown $CsPbBr_3$ MP with clear and smooth facets, and strong L_y confinement.'

Main text, page 12, Methods, added: 'The $CsPbBr_3$ microplatelets were synthesized using an anti-solvent method[8]. Glass substrates were placed in a placed in dichloromethane anti-solvent filled beaker, and the precursor solution was dropped onto the substrates, as shown in Fig. S2a. Beaker was covered with perforated Parafilm to and placed in an oven with temperature of 30 °C for 48 h, enabling evaporation and $CsPbBr_3$ single crystal growth. The synthesis procedure yields microplatelets of various lateral dimensions, shown in Fig. S2b.'

Main text, page 13, Methods, added: 'In our MP microcavities, polariton effective mass is $1.1\times10^{-5}\,m_e$-$1.5\times10^{-5}\,m_e$ for most LPBs of interest for parametric emission. Therefore, polariton thermal de Broglie wavelength[9] ($\lambda_{dB}=\sqrt{2\pi\hbar^2(m_{\mathrm{eff}}k_BT)^{-1}}$) is 1.1-1.3 μm.'

SI, page 2-3, added: Fig. S2 and Fig. S3 for material synthesis description and characterization.

Comment 3:

The authors claim that the disordered surface potentials that are formed as a result of the fabrication process are instrumental in generating fine modes and efficient parametric emission. However, the role of the disorder in enhancing parametric emission is not quantitively measured. A study of how the different levels of surface roughness effect the signal /idler ratio would be beneficial.

Response 3:

We thank the reviewers for pointing out the need for a clearer comparison to demonstrate role of disorder in enhancing parametric interactions. According to your suggestion, in the revised manuscript we have presented parametric scattering data for the MPs with comparable dimensions but different degrees of disorder. As shown in Fig. R3, we locate three samples with close parameters including L_y, detuning energy, coupling strength, and satisfied phase matching condition, ensuring that the variation in parametric ratio is mostly affected by levels of surface disorder, respectively. CW excitation (405 nm) was utilized to enhance clarity for the quantized energy levels in the linear regime (a-c), while pulsed excitation (400 nm) was applied to resolve nonlinear parametric emission above threshold (d-f).

In the weak disorder or surface-smooth case (a), regularly spaced modes with symmetric angular dispersion are resolved in the linear regime, indicating the only presence of geometric confinement. In the moderate-disorder case (b), two mode manifolds mainly dominate: the larger spacing for the sets of modes is related to geometric confinement, and the two sets of fine modes indicate the presence of disorder. In the case of strong disorder (c), multiple disorder energy levels are superposed with the geometric confinement induced energy levels and become highly quantized and hybridized.

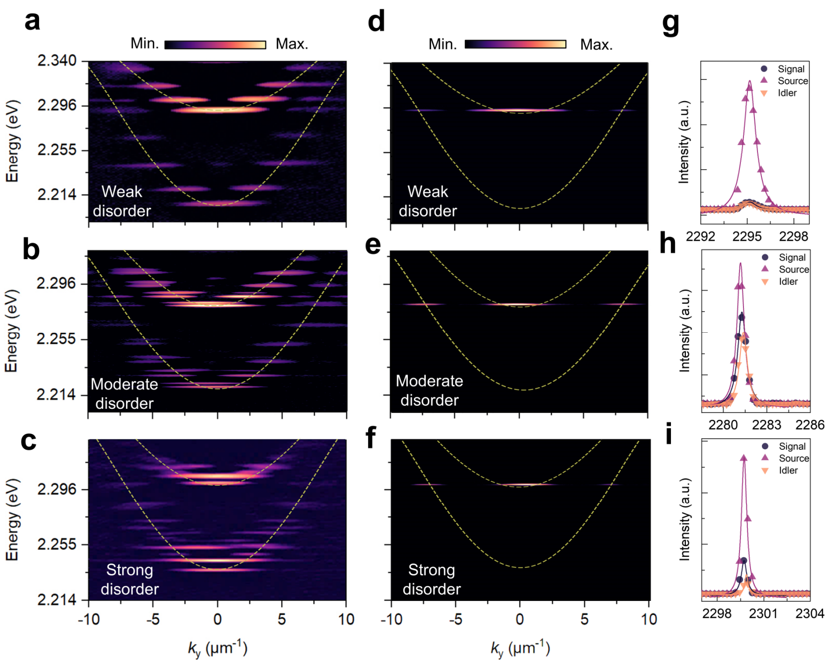

Fig. R3. (a-c) Angle-resolved PL spectra (TM polarized) of phase matched microcavities at low excitation density. a: weak disorder; b: moderate disorder; c: strong disorder. Dashed lines are guidance for LPB1 and LPB2 dispersion. (d-f) Respective angle-resolved PL spectra of phase matched microcavities above nonlinear parametric scattering threshold. d: weak disorder; e: moderate disorder; f: strong disorder. (g-i) Spectra extracted (as discussed in Fig. S9) for respective signal, idler and source emission. g: weak disorder; h: moderate disorder; f: strong disorder.

For the parametric emission (nonlinear regime), we observe the weak parametric emission in the weak disorder case (d and g), and the linewidth is relatively broad. In the moderate disorder case (e and h), strong parametric emission is observed. A further increase in disorder density (f and i) leads to a decrease in parametric emission intensity. It qualitatively suggests the presence of moderate disorder largely enhances parametric emission. The presence of disorder breaks the generalized wavevector selection rule for polariton parametric interactions and makes the process happen with less-stringent wave vector selection rules (*Phys. Rev. B* **79**, 045319 (2009), *Phys. Rev. Lett.* **106**, 126401 (2011), *Phys. Rev. Lett.* **129**, 147402 (2022)), enhancing the parametric emission efficiency. While further increasing the disorder density decreases the emission again, probably due to more randomly distributed scattering pathways (*Sci. Adv.* **11**, eadr1652 (2025)), and reduced density of states at the phase-matched energy levels.

Revision 3:

Main text, page 7, added: 'The role of confinement in enhancing polariton–polariton interactions can be anticipated from the increased wavefunction overlap and thus stronger nonlinear coupling, as has been reported in a variety of microcavity configurations[2,3,5,10]. However, in previously reported confined schemes[11,12], the parametric emission intensity is often not substantially improved compared to planar microcavities. In our case, the disorder potential acts as the key enabler for the observation of strong coherent parametric emission. To qualitatively illustrate its role, we investigated variation of parametric emission with different levels of disorder (Fig. S17). The markedly enhanced parametric emission in the presence of a moderate amount of disorder highlights its crucial importance for triggering efficient parametric emission. The disorder potential breaks overall inversion symmetry and thereby lifts the degeneracy for wavevector selection rules[2,5,13], allowing the parametric process to occur under less stringent conditions.'

SI, page 17, added Fig. S17 and respective discussions.

Comment 4:

The authors chose $CsPbBr_3$ as the active material, but the manuscript does not justify why this particular halide perovskite is uniquely suited for sustaining parametric scattering and supersolid behavior. Could other halide compositions (e.g., $CsPbI_3$, mixed halides, or hybrid organic-inorganics) offer advantages in terms of exciton binding energy, linewidth, or tunability of the cavity modes? A few sentences in the introduction would help here.

Response 4:

We thank the reviewers for raising the suggestion for discussion on choice of $CsPbBr_3$ as active gain material in our study. We chose $CsPbBr_3$ because of its relatively large exciton binding energy (~35–45 meV), which preserves robust exciton–photon coupling at room temperature, and its high PL quantum yield, which ensures efficient probing of light–matter interaction. The Rabi splitting could readily exceed 100 meV in DBR microcavities with quality factor ~1000 (*Nat. Mater.* **20**, 1315–1324 (2021)). Secondly, compared to other perovskites such as $CsPbI_3$ and 2D perovskites, $CsPbBr_3$

exhibits high stability under photo-excitation and resistance to moisture and oxygen. These advantages make it ideal for creating deterministic cavity geometries with high quality confinement without the need for complex top-down fabrication. Further considering our previous experience (*Sci. Adv.* **11**, eadr1652 (2025), *Nano Lett.* **20**, 6636–6643 (2020)) in light-matter interactions and low threshold lasing for this material, we selected it for the current work.

We agree with the reviewer's point of view that $CsPbI_3$, 2D perovskites, and many hybrid organic-inorganic materials can also be used for study of polariton physics, extending the wavelength range and enhancing nonlinearity, *etc.* However, $CsPbI_3$ has a relatively low exciton binding energy and poorer stability against moisture and oxygen. 2D perovskites have a high nonlinear coefficient, but based on our laboratory results, their stability under strong photoexcitation is not as good as that of $CsPbBr_3$. Other hybrid organic-inorganic materials may also feature high exciton binding energies, and if room-temperature condensation can be readily achieved with promising stability, they could also be suitable. The focus of the work lies in the mechanism and can be extended to these materials.

Revision 4:

Main text, page 3, added: 'The $CsPbBr_3$ was selected due to its large exciton binding energy, high photoluminescence quantum yield and good structure stability, enabling robust room-temperature polaritonics[14-17].'

Main text, page 11, added: 'These results demonstrate that non-trivial potential landscape acts as key enablers of unconventional parametric phenomena—which could be readily extended to other material platforms'.

Comment 5:

The manuscript would benefit from structural characterization of the $CsPbBr_3$ microplatelets. While the microcavities are strongly characterized, no direct evidence is provided for the crystallographic quality or phase purity of the material. Techniques such as X-ray diffraction could confirm the absence of secondary phases, strain, or structural defects that might influence the dispersion and parametric scattering behavior. Including such data would strengthen the link between the observed effects in the

strongly coupled system and the intrinsic properties of the active material, and ensure that the reported effects are not influenced by variations in material quality.

Response 5:

We thank the reviewer for this constructive suggestion on structure characterization of the $CsPbBr_3$ MPs. As the reviewer pointed out, a high density of defects would strongly suppress exciton-photon coupling and hinder the realization of polariton condensation. In this study, we have optimized the precursor solution preparation, and the anti-solvent growth process by enabling slow evaporation to enhance the crystallinity quality. Using the grown MPs, we have realized very low-threshold condensation and lasing, reported in previous work (*Sci. Adv.* **11**, eadr1652 (2025), *Nano Lett.* **20**, 6636–6643 (2020)).

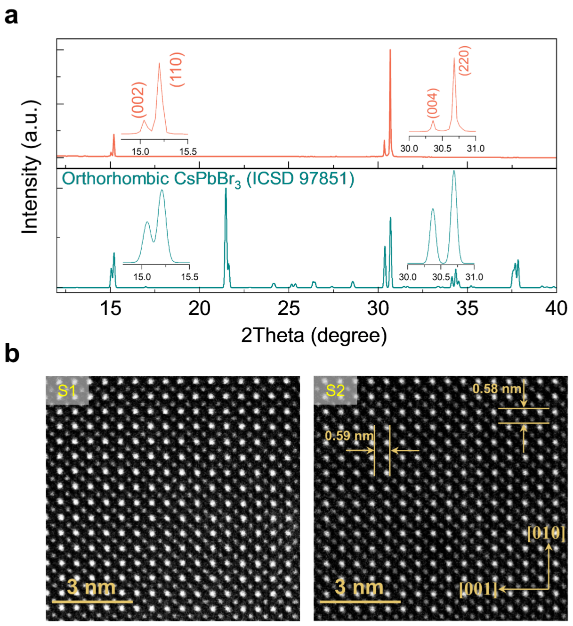

Fig. R4. Crystal structural characterization. (a) XRD characterizations of the $CsPbBr_3$ microplatelets. Diffraction peaks are labelled with crystallographic planes, corresponding to orthorhombic phase $CsPbBr_3$ (ICSD 97851) shown in lower panel. Insets show zoom-in peak profiles. (b) Annular dark field scanning transmission electron microscopy image of $CsPbBr_3$ MPs with different lateral dimensions, viewing from the [010] zone axis, showing an identical crystalline structure orthorhombic $CsPbBr_3$. Atomic-resolution images reveal an inter-plane spacing of 0.58 nm between the [010] planes and 0.59 nm between the [001] planes, respectively.

Herein, to emphasize this issue, we include XRD pattern (Fig. R4a) and angular dark field scanning transmission electron microscopy (ADF-STEM) images (Fig. R4b) to confirm the high crystallinity of the MPs. The XRD pattern shows dominant peaks at 15.04, 15.20, 30.36, and 30.68 degrees, corresponding well to the (002), (110), (004), (220), planes of orthorhombic phase $CsPbBr_3$ (lower panel, ICSD 97851). The narrow linewidth of ~0.04 degree indicates a high degree of crystallinity (*Nat Commun* **6**, 7586 (2015)). The scanning transmission electron microscopy images for a larger (Fig. R5, S1) and smaller MPs (Fig. R5, S2) show identical crystalline structure characteristic of orthorhombic phase $CsPbBr_3$ (*Nat Commun* **13**, 2587 (2022)). We have also compared the TRPL spectra (Fig. R2 in response to comment 2), and find that the PL decay curve is almost identical for different samples, suggesting the consistent optical quality across scales.

Revision 5:

SI, page 2, added Fig. S3 on structural characterizations.

Comment 6:

Given that perovskites are prone to point defects and surface traps, how do the authors ensure that these do not dominate the coherence and linewidth of the parametric emission? Is there any passivation strategy or post-treatment applied or can you comments on how that might affect properties?

Response 6:

We thank the reviewer for raising the issue of point defects and surface traps and their potential impact. The high quality of single crystal has been demonstrated in response to comment 5. Meanwhile, as used in our previous studies (*Sci. Adv.* **11**, eadr1652 (2025), *Nat. Commun.* **15**, 3345 (2024), *Nat. Commun.* **15**, 1893 (2024)) about 60 nm of PMMA layer were spin coated after transferring to bottom DBR. The PMMA acts as a physical barrier against moisture/oxygen and passivates surface defects, enhancing stability and optical properties. Such layer also protects the perovskite crystal during e-beam evaporation of top DBR.

Revision 6:

Main text, page 12, Methods section, added ‘followed by ~60 nm PMMA layer encapsulation before top mirror evaporation, acting as protection layer for perovskites.

Response to Reviewer 2

The manuscript "Harnessing highly efficient coherent polariton parametric emission in quantum confined perovskite microcavities" by Xinyi Deng et al. reports the first-time observation of resonantly enhanced parametric scattering of exciton-polaritons in a halide perovskite microcavity. The authors emphasize the key result of their work: the strong influence of lateral polariton quantization, accompanied by potential disorder, on the nonlinear optical response of the studied system. They experimentally observe coherent polariton emission involving parametric scattering and also claim the "first observation of strong phase coherence between the two parametric species in momentum space."

The importance of the observations is not in doubt: the suggested arrangement is suitable for generating phase-correlated photon pairs, with potential for operation down to the single-photon regime. The halide perovskite platform is favorable in this sense, as it is known for its ultimate quantum efficiency of exciton-enabled light emission.

The manuscript is generally well-written. However, some further clarifications and polishing of the text would be beneficial, as noted below. The team employs well-established experimental methods; therefore, the data quality is suitable. The analytical model is clear; some parameters used need to be specified in the updated version of the manuscript.

Although I am generally positive about this work, I must point out that some of the authors' claims require further justification. The authors have to address several questions before I can recommend this work for publication in Nature Communications.

Response:

We sincerely thank the reviewer for reviewing our manuscript. We appreciate the favorable evaluation of the novelty and importance of our study. The constructive suggestions on recognizing population mechanisms on the second branch helps us to develop a deeper understanding in the scattering process and identify the nonlinear origin. In response, we have added respective experimental and theoretical results, with

detailed analysis and discussion, which we believe could resolve the reviewer's concerns. Point-by-point replies are shown in light blue in the response letter, and the corresponding changes in the manuscript are indicated in blue in the response letter and revised manuscript.

Comment 1:

My primary concern is related to the mechanisms of population of signal and idler states. I can suggest at least three of those:

(1) Conventional relaxation from the reservoir to a resonant state (gain).

(2) Linear scattering of polaritons from the source to signal and idler states. (power dependence transition) not purely linear scattering.

(3) Nonlinear parametric scattering of source polaritons towards signal/idler state, claimed as the primary mechanism.

Firstly, the right panel in Fig. 2a clearly shows the strong asymmetry in signal (1.36)/idler (0.43) relative intensities. In case of mechanism (3), exclusively responsible signal/idler population, this picture must be purely symmetric. The largest of these two values is compared to other works in Fig. 2b and is referred to as "parametric emission efficiency", which might mislead the reader. This efficiency is fundamentally limited by the minimum of the two values mentioned for each particular measurement.

The authors state in the text: " Below the first threshold, incoherent relaxation from the exciton reservoir is responsible for the population at signal and idler states." It is unclear why authors neglect this mechanism when discussing the system behavior above the first thresholds.

Let me suggest an explanation of the observed effects involving only mechanisms (1) and (2). Above the first threshold, the condensate appears at the source state. The polaritons there undergo elastic scattering into signal/idler modes, with efficiency enhanced by surface disorder. These scattered polaritons seed the condensates in signal/idler states, which appear at a larger threshold and remain coherent with the source, as they are seeded by polaritons coming from it. Another reason for the signal,

source, and idler states to be mutually coherent is the complete overlap of these modes in real space.

The condensation threshold for the signal/idler states can be larger than that of the source state for several reasons, including differences in polariton lifetimes. The nearest polaritonic state below the source is more than 50 meV apart, while for signal/idler states, this distance is below 10 meV.

I firmly believe that with this massive amount of experimental data in hand, authors can distinguish between mechanisms (1-3) more accurately.

Another way to completely exclude the mechanism (1) from the list is to perform additional measurements with the resonant pumping of the source state by appropriately tuning the excitation photon energy and adjusting the spot size to avoid direct excitation of signal/idler modes. To distinguish pump photons from those in a condensate (and estimate the condensate emission rate), one can pump at an angle, staying within the positive/negative lateral wavevectors, yet remaining resonant with the source mode.

Response 1.1: Signal and idler population mechanisms

We thank the reviewer for the comprehensive analysis of the possible signal and idler population mechanisms. We performed respective experimental and theoretical analysis, from which we attribute the nonlinear coherent signal and idler emission to the nonlinear parametric scattering from source condensate.

According to your suggestion, we performed resonant/non-resonant pumping experiments for an identical sample and analyzed the intensity evolution behavior. For non-resonant pumping, a 400 nm (1 kHz, 100 fs) pulsed laser is directed onto the sample. For resonant pumping, a 540 nm (1 kHz, 100 fs) pulsed laser is utilized and filtered to have the laser energy slightly blue-detuned from the source condensate, while also avoiding excitation of the exciton reservoir. The schematic of resonant pumping can be found in Fig. S10 in the revised version of manuscript.

As shown in Fig. R5a, under non-resonant pumping conditions, the population on both LPB1 and LPB2 emerges in the linear regime, with discretized energy levels arising from confinement and disorder. Above the nonlinear parametric emission threshold (Fig. R5b, the second threshold), the signal and idler emerge at energy positions resonant with the source condensate. Fig. R5d depicts the angle-resolved PL spectra at

resonant pump condition below the nonlinear parametric scattering threshold (laser position circled out). The emergence of a flat narrow emission, highlighted by white arrows and just below the excitation energy, is the source emission. While at this stage, the emission on the LPB2 at the respective energy position is missing. As the power further increased to higher pump fluence (Fig. R5e), parametric emission appears at the LPB2 dispersion positions resonant with source condensate. Fig. R5c and f depict the respective growth rate of signal/idler intensity with respect to source emission intensity under non-resonant and resonant pump conditions, respectively. The non-resonantly pumped case features a two-threshold behavior, while resonantly pumped case exhibits a single-threshold behavior.

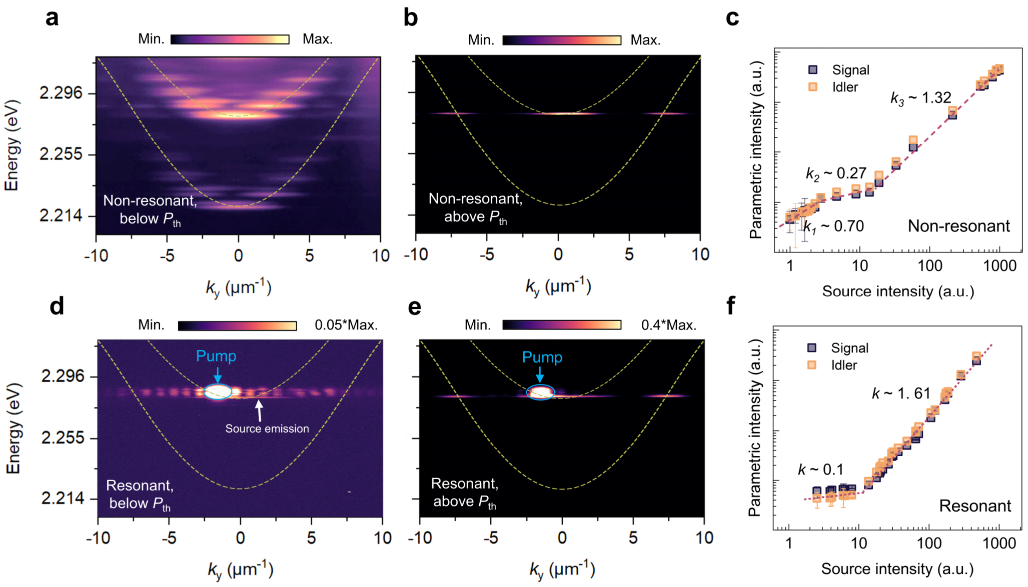

Fig. R5. (a-c) Parametric emission under non-resonant pumping condition. a: angle-resolved PL spectra below the condensation threshold; b: angle-resolved PL spectra with parametric emission; c: the respective growth behavior of signal/idler emission intensity with respect to source polariton. (d-f) Parametric emission under non-resonant pumping condition; d: angle-resolved PL spectra below the condensation threshold; e: angle-resolved PL spectra with parametric emission; f: the respective growth behavior of signal/idler emission intensity with respect to source polariton.

The population mechanism (1)(2)(3) as raised by the reviewer stem from different microscopic origins, therefore exhibit different efficiencies and growth behavior for the signal/idler states with respect to source polariton. Next, we analyze in detail to assign predominant mechanism in nonlinear regime to the nonlinear parametric scattering.

(1) Conventional relaxation from the reservoir to a resonant state:

Such possibility could be excluded with observation of nonlinear parametric emission in resonant pump condition, where exciton reservoir is absent.

(1) + (2): Linear scattering of polaritons from the source to signal and idler states, and seeded the nonlinear emission

We agree with the review that the mechansim (1)+(2) could generate strong signal/idler emission. To explore, we have carefully checked experimental data. The seeding effect, descried in this mechanism, would establish the source, signal and idler as independent species in the nonlinear regime, being continuously replenished by the exciton reservoir. However, the correlated evolution of the source, signal and idler modes, as reflected in their comparable blueshift and spectral broadening (Fig. S18), indicates that these polaritons are subject to strong mutual nonlinear interactions (*Nature* **639**, 337–341 (2025)).

Furthermore, our observations show that forming a macroscopic occupation directly at a high-*k* state on a strongly red-detuned LPB (>110 meV) requires a pump fluence of typically 100–200 $\mu J\cdot cm^{-2}$, corresponding to a high photoexcited exciton density (*Sci. Adv.* **7**, eabj7667 (2021)). Such high threshold is expected since LPB has its energy minimum at *k*~0, and such minimum is thermo-dynamically favored than a high-*k* state. Under non-resonant pumping the polaritons preferentially relax and redistributes population toward lower-energy, lower-*k* states—so a macroscopic occupation at a high-*k* point is hard to sustain unless the injection rate is very high (*Rev. Mod. Phys*. **82**, 1489–1537 (2010); *Phys. Rev. B* **62**, R2283–R2286 (2000); *Phys. Rev. B* **88**, 121303 (2013)) or with band-structure engineered to tailor local energy minimum positions (*Nat. Commun.* **4**, 1749 (2013); *Nat. Phys*. **7**, 681–686 (2011)). Consequently, the small separation between our two thresholds (7.1 $\mu J\cdot cm^{-2}$ versus 10.3 $\mu J\cdot cm^{-2}$ in Fig. R5c), would not support the hypothesis of ‘linearly scattered polariton after the first threshold seed the nonlinear parametric scattering’, since such seeding would be far too small to bridge the carrier density discrepancy required for a direct high-*k* condensate.

Finally, we attributed the mechanism that leads to nonlinear signal/idler emission as *nonlinear scattering from source polariton*. As evidenced by the comparable parametric emission ratio (Fig. R5b and e), and the respective growth rate (Fig. R5c and f), suggesting that identical mechanism dominated such processes. The effective

slope ($k \sim 1.61$) in resonant case is slightly larger than that under non-resonant ($k \sim 1.32$) excitation, due to a relatively stronger nonlinearity when the source condensate is quasi-directly fed by the fs-laser pulses (*Nat Commun* **10**, 3869 (2019)).

According to your suggestion, we have emphasized the identification of mechanism (3) in nonlinear regime and the role of mechanism of (1) and (2) in respective regimes in the revised version of manuscript.

Revision 1.1:

Main text, page 5, added 'Furthermore, to recognize the population mechanism for nonlinear parametric emission, resonant and non-resonant pump were performed in an identical microcavity. The comparable parametric ratio and intensity growth behavior suggested the dictating role of nonlinear parametric scattering, rather than direct condensation fed by the exciton reservoir (Fig. S10-12).'

SI, page 11-13, added part IX on 'Identification of signal and idler state population mechanism'.

Response 1.2: Theoretical simulation of resonant excitation

Next, we show theoretically that the 'resonant' excitation on the LPB1 will induce parametric emission only when nonlinear coupling term is introduced for LPB1 and LPB2.

Our theoretical model is based on the following two-component driven-dissipative Gross-Pitaevskii equation:

$$i\hbar \frac{\partial \psi_n(r,t)}{\partial t} = \left[\hat{E}_n^{\mathrm{LP}} + V_{dis}(r) - \frac{i}{2}\big(\gamma - P_n(r,t)\big) - i\Gamma|\psi_n(r,t)|^2\right]\psi_n(r,t) + 2\alpha\psi_m(r,t)^2\psi_n^*(r,t) + 2\alpha\psi_m(r,t)^2\psi_n(r,t)$$

where $n = 1,2$ represent the index of the different lower polariton branches (here we considered two such branches, see the Fig. below). In our theoretical model, we have explicitly considered that the gain function of the second branch $P_2(r,t) = 0$. We pump only the first mode with a Gaussian profile $P_1(r,t) = P_0 \exp[-\frac{r^2}{L^2} - \frac{t}{\tau}]$. This choice of the pump scheme enforces that there is no gain in the second branch except

the polariton transfer from the first branch to second one through the nonlinear parametric process. We have confirmed this by switching off the nonlinear term in the driven-dissipative Gross-Pitaevskii equation (see the Fig. below). We find that for a linear regime second branch shows no polariton population, which is in good agreement with the experiment. Though the linear scattering regime as we observe in first sublinear period (Fig. R5f) in resonant pumping scheme could not be resolved here, because the coupling in driven-dissipative Gross-Pitaevskii equation could only describe nonlinear coupling process (*Rev. Mod. Phys.* **85**, 299–407 (2013)), as also described in Methods section.

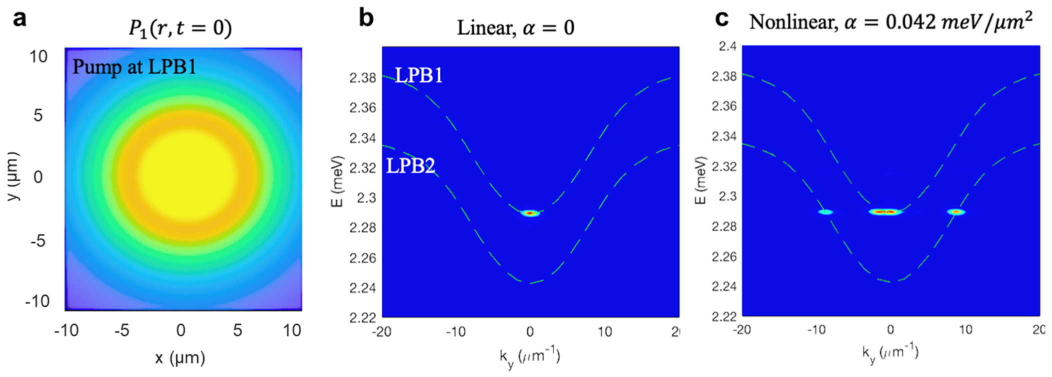

Fig. R6. (a) Gain profile of lower polariton branch 1 (LPB1) $P_1(r, t = 0)$, while the gain profile of the second branch (LPB2) is considered zero $P_2(r, t) = 0$. (b) Dispersion obtained without the nonlinear terms ($\alpha = 0$). As we can see, in the absence of the nonlinear term, no effective gain exists in the second branch. (c) Dispersion obtained with the nonlinear terms ($\alpha \neq 0$) causing the parametric scattering. Here the system size is 3.6 µm, and OPO ratio ~ 0.82.

Revision 1.2:

SI, page 26, added part XVII on Theoretical simulation of resonant pump with, and without nonlinear LPB1 & LPB2 coupling.

Response 1.3: Evaluation of parametric emission 'ratio', and efficiency

We agree with the reviewer that the parametric emission intensity should not be higher than the pumping intensity which is related to applied laser pump fluence. While herein the "source" intensity is attributed to the source condensate intensity which would be much lower than the applied non-resonant pump laser, thus an above-unity idler/source

(remaining source) emission ratio is possible considering the multiple population and depletion mechanisms involved. An above-unity signal-to-source ratio indicates a stimulated regime with depletion of the source state and suggests highly efficient nonlinear parametric emission, a characteristic that has likewise been reported in pure-photonic OPOs (*Opt. Lett.* **21**, 1336–1338 (1996), *Opt. Lett.* **26**, 620–622 (2001)). And the depletion of pump/source polariton has also been observed in intrabranch polariton OPOs (*Phys. Rev. Lett*. **90**, 106401 (2003), *Phys. Rev. Lett.* **91**, 107402 (2003)) in the stimulated regime. In addition, exciton reservoir provides small amount of seeding in this nonlinear regime, triggering the stimulated process to reach larger population. In our theoretical study, the above-unity ratio has also been observed as shown in data points in Fig. 3a.

In our microcavities in an energy-degenerate interbranch parametric scattering process, components near $k_y = 0$ would serve as the most effective source to create parametric polariton pairs at opposite in-plane wavevectors, due to the highly satisfied phase matching condition. Therefore, we extracted the spectra from central regions (integrated over 7 pixels in the angular direction) of the respective states (Fig. S9), considering that the source emission is broadened due to spatial confinement. On the other hand, the 'ratio' values for some literatures (*Nano Lett.* **22**, 2023–2029 (2022), *Phys. Rev. B* **106**, L020301 (2022), *Phys. Rev. B* **107**, L060303 (2023), *Nano Lett.* **22**, 3026–3032 (2022)) are not directly mentioned, therefore we extract the value (maximum) by reading the RGB color in the color bar. For the literature (*Nature* **639**, 337–341 (2025), *Nano Lett.* **21**, 3120–3126 (2021), *Phys. Rev. Lett.* **108**, 166401 (2012)) that directly gave the value, we utilized their given values and checked in respective angle-resolved plots that the values are not underestimated.

According to reviewer's suggestions, we have revised the term in main text from '*efficiency*' exclusively into '*parametric ratio*', and highlighted the feasibility of above -unity ratio, the method for extracting parametric ratio in the revised version of manuscript and SI. While we keep the title as 'highly efficient', since the general parametric emission intensity is still higher than it is normally observed in planar microcavities.

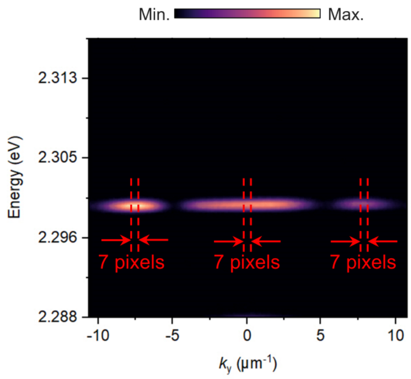

Fig. R7. Angle-resolved PL spectra showing areas used for integration and evaluation of parametric ratio. The angle resolved emission collected from the objective (100x, NA=0.9) was mapped to 166 pixels (angular direction) on the Fourier plane in the spectrometer. The emission centered around (red dashed lines) the respective states with 7 pixels were integrated in the angular direction for evaluation.

Revision 1.3:

Main text, page 4, added 'In the energy-degenerate interbranch parametric scattering process, components near k_y = 0 would serve as the most effective source to create parametric polariton pairs at opposite in-plane wavevectors, due to the highly satisfied phase matching condition[18-21]. Therefore, we extracted the spectra from central region spectra of the respective states (Fig. S9), considering that the source emission is broadened due to spatial confinement[7].'

Main text, page 5, added 'The above-unity signal-to-source ratio suggests depletion of the source state in the stimulated regime and indicates highly efficient nonlinear parametric process, a characteristic that has likewise been reported in pure-photonic[22,23] OPOs. The depletion of source state has also been observed in polariton OPOs in the stimulated regime[24,25].In our theoretical study, an above-unity ratio has also been observed as presented in the following discussion (Fig. 3a).'

Main text, page 6, Fig. 2b caption, added: 'A comparison of parametric emission ratio and linewidth of our work with reported literature values (based on given data[10,11,23] or estimated from color-scale maximum[13-16]).'

SI, page 9, added Fig. S9 on Schematic for extraction of spectrum in Fig. 2a, and evaluation of parametric ratio.

Response 1.4: Mechanism for imbalance behavior.

The unbalanced intensity of signal and idler is induced by *asymmetry in both population and depletion pathways* in the driven-dissipative system, which are further magnified in presence of surface disorder that breaks inversion symmetry. Such imbalance existing even in the nonlinear regime has been theoretically discussed (*Phys. Rev. B* **93**, 064203 (2016)) and experimentally observed in polariton nonlinear emission modulated by trap or disorder (*Phys. Rev. Lett.* **104**, 126403 (2010), *Nat. Commun.* **13**, 3080 (2022)). The emission intensity reflects averaged gain and loss at signal/idler in the driven dissipative unequilibrium system. Exciton relaxation and scattering from the source populate the signal and idler states, whereas radiative recombination and relaxation into LPB2 lower energy states act as loss channels. Due to broken inversion symmetry, the energy levels and scattering pathways become intrinsically asymmetric, which distort these linear processes and bias the subsequently stimulated scattering and population in nonlinear regime, resulting in the asymmetric/imbalanced angular emission as we observed.

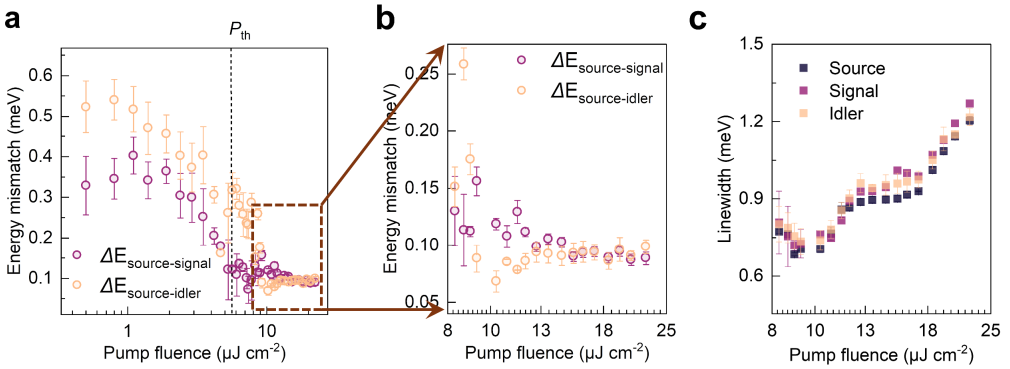

Fig. R8. (a) Evolution of source to signal, and source to idler energy mismatch with pump fluence from 0.3 to 27.5 μJ·cm^{-2}. The dashed line indicates condensation threshold. (b) Zoom-in view of energy mismatch in dashed circled area from 8.0 to 25.0 μJ·cm^{-2}. (c) Corresponding linewidth broadening for source, signal, and idler, with pump fluence from 8.0 to 25.0 μJ·cm^{-2}. The source to signal, and source to idler mismatch tend to saturate, accompanied by continuous broadening in linewidth.

Furthermore, a nonlinear and driven–dissipative behavior of the parametric oscillator could be reflected in the evolution of the energy mismatch in the nonlinear regime. Above the nonlinear parametric emission threshold (Fig. R8), the mismatch tends to

saturate, while the linewidth continues to broaden, revealing that nonlinear interactions gradually drive the populations towards a dynamic equilibrium and pin the mismatch to an almost constant value. In addition, in a theoretically simulated nonlinear parametric oscillator as we show in Fig. R6c, the emission intensity is unbalanced as well, supporting that the imbalance is theoretically applicable.

According to your suggestion, we have emphasized the mechanism for the imbalanced signal and idler emission in the revised version of manuscript.

Revision 1.4:

Main text, page 5, added: ‘An imbalance in the intensity of signal and idler emission could also be observed, which is expected in these microcavities dressed with disorder and confinement[3,26,27], considering the asymmetry in both population and depletion pathways in the driven-dissipative system.’

Comment 2:

Additional related question: What are the values of net gains P_n for signal, source, and idler used for the modelling in Fig. S11?

Response 2:

In the supersolid phase simulation corresponding to Fig. S11 (now as Fig. S22), we model the net gain for the source by setting the decay rate to $\gamma = 0.5\, meV$ and by using the following pump profile for the LPB1 mode:

$$P_1(r,t) = P_0 \exp[-\frac{r^2}{L^2} - \frac{t}{\tau}],$$

with $P_0 = 11.6\, meV$, $\tau = 50\, ps$, and a pump spot size $L = 11.7\, \mu m$. Although the applied non-resonant pulsed pump is on the femtosecond scale, the *effective* temporal profile experienced by the system is governed by the exciton reservoir. This reservoir acts as an incoherent pump with a phenomenological lifetime much longer than 100 fs, thereby setting the relevant time scale for the non-resonant pumping process. In our theoretical model, there is no linear process existing between LPB1 and LPB2.

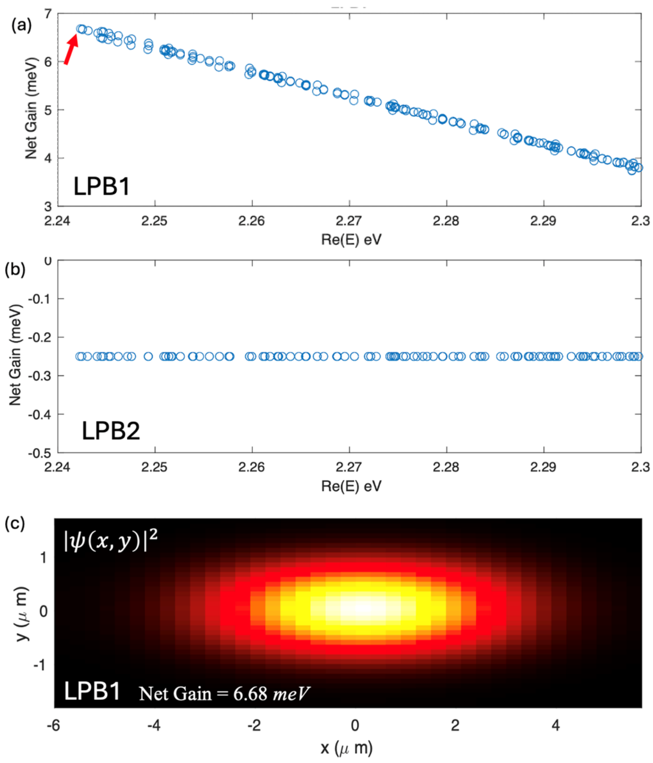

Fig. R9. (a) The net gain of the LPB1 band, extracted from the imaginary part of the corresponding energy eigenvalues. The mode with the highest positive net gain (6.68 meV), indicated by the red arrow, identifies the state in which the source condensate forms. (b) The net gain of the LPB2 band, where all values are negative, reflecting the shorter lifetime of these modes and the absence of external pumping. (c) The spatial density profile of the LPB1 mode with the largest net gain, illustrating the real-space distribution of the condensate.

The net gain at $t = 0$ for the modes in LPB1 is shown in Fig. R9a and is obtained from the imaginary part of the energy eigenvalues of the corresponding Hamiltonian. As no external gain is applied to LPB2 in the model, all LPB2 modes exhibit negative net gain (Fig. R9b), reflecting losses arising solely from their finite lifetimes.

According to your suggestion, we have added the respective discussion on the gain for the different polariton modes in the supplementary information.

Revision 2:

SI, page 25, added Fig. S23 and discussions on gain profile.

Comment 3:

In the manuscript, only the elastic scattering process is discussed. Meanwhile, Fig. 2c reveals an apparent spectral mismatch between signal, source, and idler emission peaks. Owing to the inversion symmetry of the system, is there any explanation for this effect? Could it be a measurement artifact, such as a slight misalignment of the CCD camera, which needs to be corrected during data post-processing?

Response 3:

We thank the reviewers for the suggestion on checking the measurement artifact and suggestion of explanation. We included the mechanism explanation on both energy mismatch and intensity imbalance in response to comment 1.

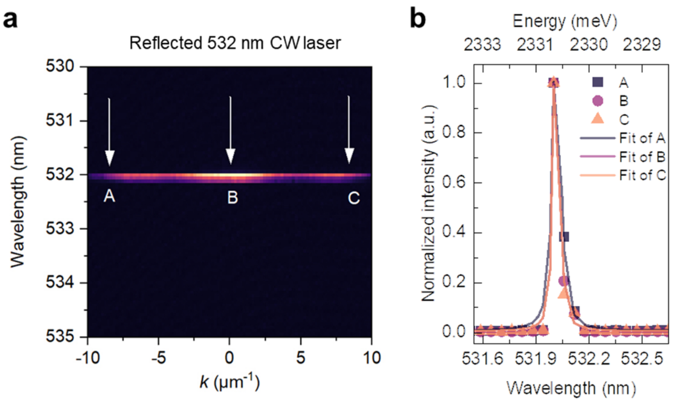

Fig. R10. (a) Angle-resolved spectrum of a 532 nm laser. The laser was focused in real space and therefore angularly dispersed in Fourier space. (b) Normalized spectrum extracted at position A, B, C indicated by the white arrow in (a). The overlapped spectrum indicates identical dispersive feature at different positions on CCD camera, excluding the system error for the imbalanced signal/idler emission.

Here we present the analysis on exclusion of measurement artifact. We utilized a single-wavelength 532 nm CW laser to resolve the spectrum in Fourier space. The laser was focused on the focal plane and thereby dispersed in Fourier space. The corresponding angle-resolved spectrum is shown in Fig. R10a. The spectrum at the pixels corresponding to typical positions (source, signal, and idler) in the parametric oscillator were extracted and plotted in Fig. R10b. As illustrated by the normalized spectrum, the sharp-peaked emissions all centers around the same pixel in the wavelength axis. Therefore, the parametric emission with slightly mismatched energy reflects the

intrinsic property of the parametric oscillator. In addition, we checked the energy mismatch by flipping the sample in *y* direction and observed identical mismatch towards the respective direction, excluding the system error as well.

According to your suggestion, we have revised the manuscript and added respective discussion on asymmetry mechanism, and exclusion of system error.

Revision 3:

SI, page 11, added Fig. S18(d) (e) for exclusion of system error when estimating energy mismatch.

Comment 4:

In the text, the authors compare confined and unconfined microcavities. Unfortunately, I was unable to find the two respective definitions. It is worth clarifying this for the reader. I suggest adding a discussion on the role of the second dimension of the rectangular microcavities. Why can the confinement in the second dimension be neglected?

Response 4:

We thank the reviewer for the suggestion of clear definition in confinement size and consideration of second dimension. The MPs typically exhibit an anisotropic lateral dimension with L_x (length of *x*-axis) > L_y (length of *y*-axis). The L_x is generally no less than 10 μm, while L_y can be as short as around 2 μm (Fig. S2). While polariton thermal de Broglie wavelength ($\lambda_{dB}=\sqrt{2\pi\hbar^2(m_{\text{eff}}k_BT)^{-1}}$, *Phys. Rev. Lett.* **121**, 067401 (2018)) is 1.1-1.3 μm. Therefore, the confinement effect is considered according to L_y dimension. According to the model of quantum size effect as displayed in Fig. 3, an evident increase in parametric emission ratio emerges at L_y ~4 μm, which we use as the 'boundary' for different levels of confinement. To make the statement clear, according to your suggestion, we revise the 'confined' and 'non-confined' terminology to '*strongly confined*' and '*weakly confined*', referring respectively to samples with L_y *smaller than* or *larger than* 4 μm.

In response to your suggestion, we have revised the manuscript, added descriptions of sample geometry in the respective discussion in Fig. 1 and discussed how the geometry

defines the 'confinement' as we studied in this work. The definition for 'strongly confined' and 'weakly confined' is added in this part and utilized throughout the manuscript.

Revision 4:

Main text, page 3, added: 'The MPs were fabricated by an anti-solvent route (Fig. 1b)[8], which exhibit an anisotropic lateral dimension with L_x (length of x-axis) > L_y (length of y-axis). The L_x is generally no less than 10 μm, while L_y can be as short as around 2 μm (Fig. S2). Nevertheless, the MPs feature smooth facets and exhibit consistent optical properties across the relevant range of lateral dimensions (Fig. S2, S3). Considering the polariton thermal de Broglie wavelength (1.1-1.3 μm, Methods)[9], the influence of L_x could be neglected, and we therefore consider the spatial confinement and energy quantization according to L_y dimension[7]. Since the high-efficiency parametric emission emerges when L_y decreases to 4 μm as shown in later discussions, we define the regime where L_y is 'smaller than' 4 μm as 'strongly confined' and L_y is 'larger than' 4 μm as 'weakly confined', respectively.'

Main text, page 4, the caption of Fig.1, added: 'Inset shows cross-sectional view of the MP typical anisotropic dimension with y direction dimension (L_y) approximately 2-4 μm, and the thickness typically ranging from 1 to 3 μm. Surface disorders are formed by a stamp transfer process. (b) Scanning electron microscope (SEM) image of an anti-solvent grown $CsPbBr_3$ MP with clear and smooth facets, and strong L_y confinement.'

Main text, page 12, Methods, added: 'The $CsPbBr_3$ microplatelets were synthesized using an anti-solvent method[8]. Glass substrates were placed in a placed in dichloromethane anti-solvent filled beaker, and the precursor solution was dropped onto the substrates, as shown in Fig. S2a. Beaker was covered with perforated Parafilm to and placed in an oven with temperature of 30 °C for 48 h, enabling evaporation and $CsPbBr_3$ single crystal growth. The synthesis procedure yields microplatelets of various lateral dimensions, shown in Fig. S2b.'

Main text, page 13, Methods, added: 'In our MP microcavities, polariton effective mass is $1.1\times10^{-5}\,m_e$-$1.5\times10^{-5}\,m_e$ for most LPBs of interest for parametric emission. Therefore, polariton thermal de Broglie wavelength[9] ($\lambda_{dB}=\sqrt{2\pi\hbar^2(m_{\text{eff}}k_BT)^{-1}}$) is 1.1-1.3 μm.'

SI, page 2-3, added: Fig. S2 and Fig. S3 for material synthesis description and characterization.

Comment 5:

I was also unable to find an explicit discussion of the role of potential disorder. How does surface roughness improve the system's performance? One aspect could be as follows. Polariton, scattering on surface roughness, may acquire an additional portion of momentum, inversely proportional to the lateral size of the surface feature. This process, therefore, enhances scattering from the source to signal/idler states.

Response 5:

We thank the reviewer for suggesting a more explicit discussion on role of disorder. We added in the respective parts of the manuscript to discuss how it assists a more efficient parametric process and added respective literature references.

Revision 5:

Main text, page 3, added 'For the moderate disorder regime, additional energy levels arise with an overall narrowed linewidth, making the dispersion further enriched to fulfill phase matching condition and to enhance polariton parametric interactions through lifted degeneracy[2-6].'

Main text, page 7, added: 'The role of confinement in enhancing polariton–polariton interactions can be anticipated from the increased wavefunction overlap and thus stronger nonlinear coupling, as has been reported in a variety of microcavity configurations[2,3,5,10]. However, in previously reported confined schemes[11,12], the parametric emission intensity is often not substantially improved compared to planar microcavities. In our case, the disorder potential acts as the key enabler for the observation of strong coherent parametric emission. To qualitatively illustrate its role, we investigated variation of parametric emission with different levels of disorder (Fig. S17). The markedly enhanced parametric emission in the presence of a moderate amount of disorder highlights its crucial importance for triggering efficient parametric emission. The disorder potential breaks overall inversion symmetry and thereby lifts the degeneracy for wavevector selection rules[2,5,13], allowing the parametric process to occur under less stringent conditions.'

Main text, page 7, added: 'When the system size (predominated by confinement L_y in $CsPbBr_3$ microcavity) becomes comparable to de-Broglie wavelength[9], disorder potential would induces a strong sensitivity of nonlinear processes to system parameters like energy level position, energy level density of states, *etc.'*

Main text, page 7, added: 'Disorder in such quantum confinement configuration induce significant fluctuations in energy positions and density of states for signal/source/idler on respective LPBs even with small perturbations, strongly affecting the phase matching conditions and final state population density to stimulate efficient parametric scattering.'

SI, page 17, added part XIII and Fig. S17 and discussions.

Comment 6:

Authors state: "Meanwhile, downscaling the confinement dimension leads to energy eigenstates with larger spacing that induces the randomness in matching of source and signal/idler energy position, leading to a larger fluctuation amplitude in parametric interaction strength and acquisition of highly efficient parametric emission in confined microcavities." Indeed, the LA1 and LA2 branches have generally different sets of quantized levels. The detuning (and overlap) between the fundamental level of LA1 (the source state) and the nearest LA2 state is expected to significantly affect both linear and nonlinear elastic scattering processes in the system. In this context, would the data presented in red symbols in Fig. 3a, showing nearly random behavior, become better structured when used to correlate signal&idler/source with the LA1-LA2 nearest-level detuning? Of course, the randomness induced by surface roughness will stand.

Response 6:

We thank the reviewer for the constructive suggestion on correlating detuning/mismatch with the size vs. parametric ratio plot. To do this we plot the data points in Fig. 3 in a 3D configuration, where one axis represents confinement dimension and another axis represents the energy mismatch/detuning, as shown in Fig. R13. A trend could be observed that when the L_y scales down and when mismatch is smaller, efficiency reaches higher values. In addition, most of the parametric oscillators have an energy mismatch smaller than 0.35 meV. Such value is less than full width half maximum of the source/signal/idler emission, suggesting a quasi-phase matching

condition is still needed to enable efficient parametric processes (*Nonlinear Optics* (Academic Press, 2019), *Phys. Rev. Lett.* **84**, 1547–1550 (2000), *Nature* **414**, 731–735 (2001)). Meanwhile, in our microcavities, the parametric ratio would not only be determined by such energy conservation but are also influenced by the density of available final states (*Phys. Rev. Lett.* **84**, 1547–1550 (2000), *Nat. Nanotechnol.* **17**, 396–402 (2022)) within the quantized polariton energy levels, and modified linear scattering channels assisted by the surface disorder as well as mentioned by the reviewer. The quantum size effect reflects a collective effect of rapid fluctuation in these parameters (energy mismatch, density of states, *etc.*) that finally gives fluctuating macroscopic order parameters (*Science* **306**, 1915–1917 (2004), *Nat. Mater.* **9**, 550–554 (2010), *Rep. Prog. Phys.* **77**, 116503 (2014)) which reflect as parametric ratio in this case and give rise to largely enhanced emission in the strongly confined scenario.

Therefore, we kept Fig. 3 as originally sketched and added the 3D histogram chart as supporting information to reflect the respective energy mismatch values in the model, as suggested by the reviewer. In the revised version of manuscript, we have also highlighted the influence of energy mismatch on the intensity of parametric emission.

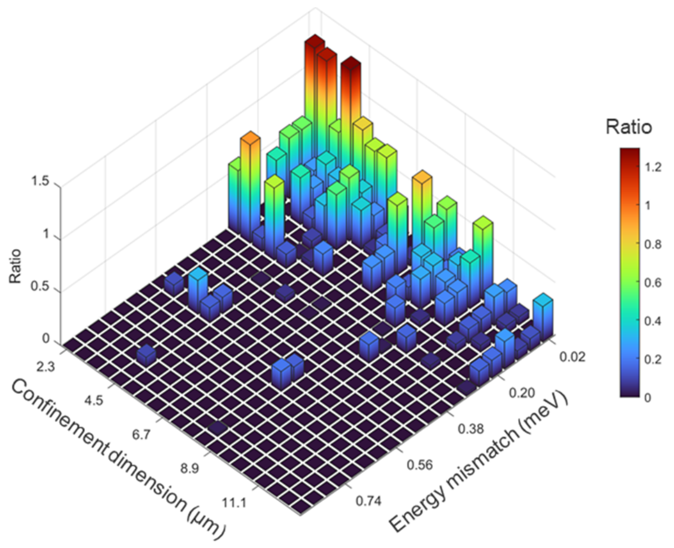

Fig. R11. The parametric ratio in relation to both confinement dimension and LPB1/LPB2 energy mismatch. When confinement scales down and when mismatch is smaller, efficiency reaches higher values.

Revision 6:

Main text, page 8, added 'The fine-energy-matching mechanism could be reflected in the gradually decreasing energy mismatch of signal/source, idler/source with increasing pump fluence (Fig. S18) and acquisition of highly efficient parametric emission in strongly confined microcavity with small mismatch (shown in Fig. S19 in a histogram map that correlates energy mismatch into the counted ratio).'

SI, page 20, added Fig. S19.

Comment 7:

Within the text, one can find many terms, probably referring to the exact property of the system under study: surface potential landscape, disorder potential landscape, disordered surface potential landscape, transfer-induced potential landscape, disorder potential, etc. I recommend leaving a single term.

Response 7:

We thank the reviewers for the suggestion on a more consistent terminology across the manuscript. We have revised all the terms as surface disorder and disorder potential landscape.

Revision 7:

Main text, and SI, change the respective terms to 'surface disorder' or 'disorder potential'.

Comment 8:

Fig. S2 ("microscope" misspelled)

Response 8:

We thank the reviewers for pointing out the misspelling, we have corrected it in the revised supplementary information.

Revision 8:

SI, page 9, 'microscopr' revised as 'microscope'.

Comment 9:

Fig. S5 (d-f) - is it a single frequency or summation over a range?

Response 9:

Fig. S5 (d-f) are k-space resolved emission collected over the complete range of wavelength, without spectral filtering, except for 405 nm long pass filter for the pump laser.

Revision 9:

SI, page 21: Added Figure S20 (d-f) caption: 'no additional spectral filtering was applied when collecting the momentum space image.'
SI, page 21, removed Fig. S20 (g-i) power-dependent intensity/linewidth/blueshift evolution. Power dependence behavior was added in Fig. S12 for the respective comparison with resonant excitation and discussion on population mechanism.

Comment 10:

Fig. S6 (b) and (a) should be exchanged.

Response 10:

We thank the reviewers for pointing out the error. In the revised version of manuscript, we made corresponding revisions.

Revision 10:

SI, Page 14: replace (b) momentum and (a) coordinate space' with '(a) momentum and (b) coordinate space'.

Comment 11:

Fig. S7 (g) instead of (b). It is unclear why the source and idler energy- and wavevector-dependent spectra are plotted on different axes. I suggest using the exact representation and adding the signal plots as well.

Response 11:

We thank the reviewers for the suggestion of plotting both signal and idler here. We now plot both 'signal' and 'idler' emission, as shown in Fig. R12.

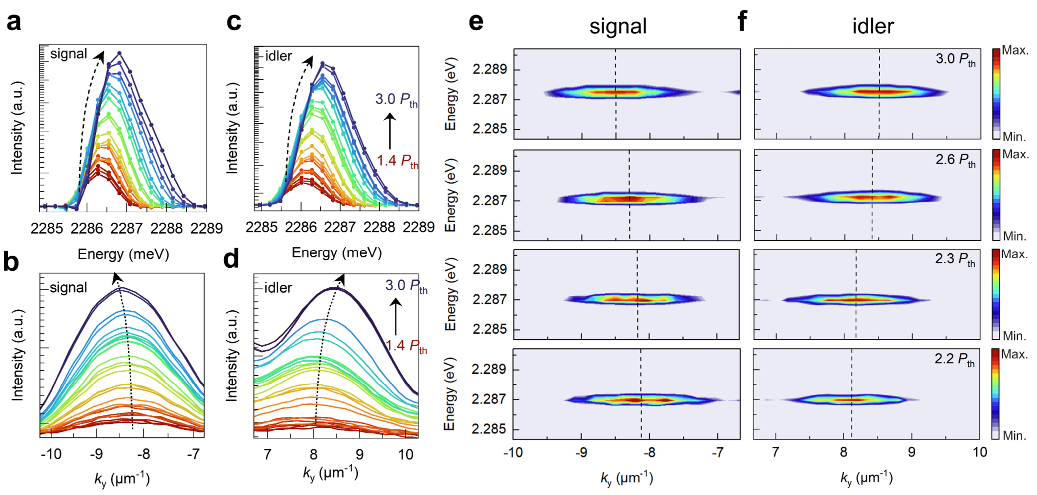

Fig. R12. (a) Energy-resolved spectra for the 'signal' emission with increasing pump fluence. (b) Wavevector-resolved spectra for the 'signal' emission extracted at peak center (maximum) in (a). (c) Energy-resolved spectra for the 'idler' emission with increasing pump fluence. (d) Wavevector-resolved 'spectra' extracted at energy positions in (c). (e-f) Zoomed-in angle-resolved PL emission of (e) signal and (f) idler at four different pump densities, with black solid line as reference to highlight relative wavevector shift.

Revision 11:

SI, page 14, revised as Fig. S13.

Response to Reviewer 3

I co-reviewed this manuscript with one of the reviewers who provided the listed reports. This is part of the Nature Communications initiative to facilitate training in peer review and to provide appropriate recognition for Early Career Researchers who co-review manuscripts.

Response:

We thank the reviewer for co-reviewing our manuscript. The comments are responded with point-to-point responses and revisions in manuscript.

Response to Reviewer 4

The work by Deng et al. reports on the efficient generation of parametric emission in a polaritonic microcavity. The authors claim that the presence of random lateral potentials is responsible for the observed anomalous enhancement of parametric scattering. To support their claims, they first present a comparison among three configurations: a non-confined microcavity (showing low-efficiency scattering), a laterally confined cavity without random potential (showing no parametric scattering), and a laterally confined cavity with random potential (exhibiting highly efficient scattering). They then perform statistical analysis to justify the observed high efficiency and develop a theoretical model. Finally, they also present evidence of supersolidity at room temperature.

The topic is certainly interesting, and the overall idea of investigating the role of disorder and lateral confinement in the regime of nonlinear polariton scattering is nice. However, I found some aspects of the work problematic and the final claims somewhat unclear. Overall, although the work has potential, I cannot recommend publication of the manuscript in its current form. A clear and convincing demonstration of the key claims—particularly regarding the role of disorder and the reported efficiency—is necessary before the work can be considered for publication.

Response:

We thank the reviewer for reviewing our manuscript and appreciate the reviewer's acknowledgment of the novelty and scientific significance of coherent parametric emission. We are grateful for the constructive suggestions which largely helped in strengthening the work. Accordingly, we have added further experimental data and expanded the analysis and discussion, and we believe these updates could address the questions raised. For clarity, point-by-point responses appear in blue in the response letter, and all revisions in the manuscript and supplementary information are shown in blue.

Comment 1.1:

The authors attempt to highlight the role of disorder, suggesting that scattering is not observed in a similar laterally confined structure without disorder. This implies that

disorder is a fundamental requirement. However, a close examination of Fig. S9 clearly shows that phase matching cannot be satisfied in that case. Therefore, the comparison is not accurate, and the authors' claim appears incorrect, especially considering that parametric scattering has been previously observed in laterally confined systems (e.g., 10.1103/PhysRevB.87.155302). Indeed, they later acknowledge that downscaling the lateral confinement introduces randomness in the phase-matching conditions, leading to larger fluctuations in the occurrence of highly efficient parametric emission. However, it is unclear what the authors are precisely claiming here. If they intend to argue that strong and coherent parametric emission is uniquely associated with laterally confined disordered microcavities, then they should present a reference sample—free from disorder but with well-matched phase-matching conditions—for a meaningful comparison. Otherwise, the role of disorder appears to be limited to mode broadening which facilitate phase matching, which is less significant.

Response 1.1

We thank the reviewers for the constructive suggestions on presenting the role of disorder with more rigid comparison and analysis. According to your suggestion, in the revised manuscript we have presented parametric scattering data for the MPs with comparable dimensions but different degrees of disorder. As shown in Fig. R13, we locate three samples with close parameters including L_y (3.4 μm, 3.8 μm, 3.8 μm, respectively for a, b, c), detuning energy, coupling strength, and satisfied phase matching condition, ensuring that the variation in parametric ratio is mostly affected by levels of surface disorder, respectively. CW excitation (405 nm) was utilized to enhance clarity for the quantized energy levels in the linear regime (a-c), while pulsed excitation (400 nm) was applied to resolve nonlinear parametric emission above threshold (d-f).

In the weak disorder or surface-smooth case (a), regularly spaced modes with symmetric angular dispersion are resolved in the linear regime, indicating the only presence of geometric confinement. In the moderate-disorder case (b), two mode manifolds mainly dominate: the larger spacing for the sets of modes is related to geometric confinement, and the two sets of fine modes indicate the presence of disorder. In the case of strong disorder (c), multiple disorder energy levels are superposed with the geometric confinement induced energy levels and become highly quantized and hybridized.

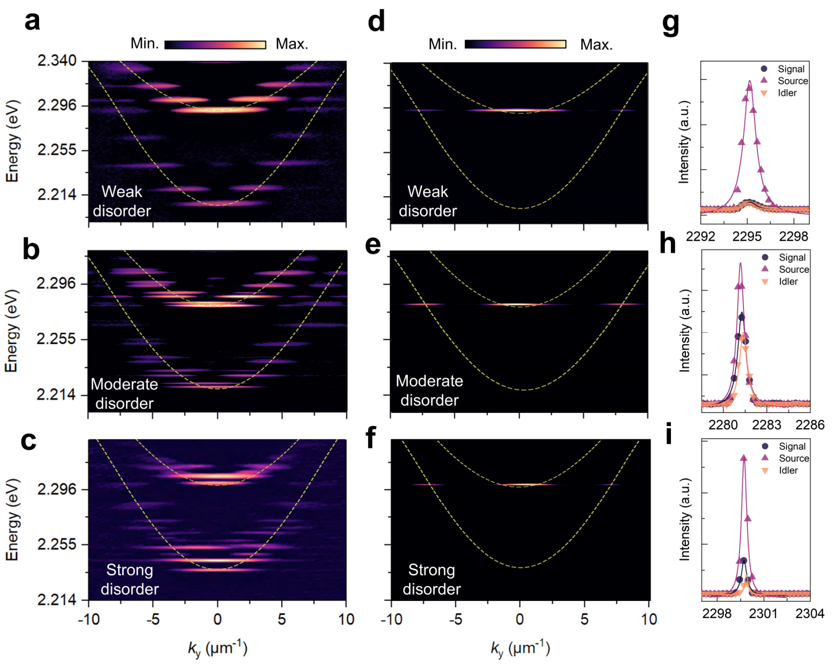

Fig. R13. (a-c) Angle-resolved PL spectra (TM polarized) of phase matched microcavities at low excitation density. a: weak disorder; b: moderate disorder; c: strong disorder. Dashed lines are guidance for LPB1 and LPB2 dispersion. (d-f) Respective angle-resolved PL spectra of phase matched microcavities above nonlinear parametric scattering threshold. d: weak disorder; e: moderate disorder; f: strong disorder. (g-i) Spectra extracted (as discussed in Fig. S9) for respective signal, idler and source emission. g: weak disorder; h: moderate disorder; f: strong disorder.

For the parametric emission (nonlinear regime), we observe the weak parametric emission in the weak disorder case (d & g), and the linewidth is relatively broad. In the moderate disorder case (e & h), strong parametric emission is observed. A further increase in disorder density (f &i) leads to a decrease in parametric emission intensity. These data suggest enhanced emission in presence of disorder. The presence of disorder breaks the generalized wavevector selection rule for polariton parametric interactions and makes the process happen with less-stringent wave vector selection rules (*Phys. Rev. B* **79**, 045319 (2009), *Phys. Rev. Lett.* **106**, 126401 (2011), *Phys. Rev. Lett.* **129**, 147402 (2022)), enhancing the parametric emission efficiency. While further increasing the disorder density decreases the emission again, probably due to more randomly distributed scattering pathways (*Sci. Adv.* **11**, eadr1652 (2025)), and reduced density of states at the phase-matched energy levels.

Revision 1.1

Main text, page 7, added: 'The role of confinement in enhancing polariton–polariton interactions can be anticipated from the increased wavefunction overlap and thus stronger nonlinear coupling, as has been reported in a variety of microcavity configurations[2,3,5,10]. However, in previously reported confined schemes[11,12], the parametric emission intensity is often not substantially improved compared to planar microcavities. In our case, the disorder potential acts as the key enabler for the observation of strong coherent parametric emission. To qualitatively illustrate its role, we investigated variation of parametric emission with different levels of disorder (Fig. S17). The markedly enhanced parametric emission in the presence of a moderate amount of disorder highlights its crucial importance for triggering efficient parametric emission. The disorder potential breaks overall inversion symmetry and thereby lifts the degeneracy for wavevector selection rules[2,5,13], allowing the parametric process to occur under less stringent conditions.'

SI, page 17, added Fig. S17 and respective discussions.

Comment 1.2

Even the comparison with the non-confined system is problematic. The sample shown in Fig. S8 exhibits a more positive detuning (with visible lasing around 2323 meV), while the confined-disordered cavity is more negatively detuned (~2300 meV). This results in a higher excitonic fraction for the non-confined sample, which strongly influences both the visibility of the parametric emission and, in fact, the condensate itself. The condensate at k=0 in the non-confined sample appears to have lower visibility compared to that of the confined-disordered cavity. Therefore, this comparison does not convincingly support the claims regarding coherence.

Response 1.2

We thank the reviewer for the valuable suggestion and pointing out this issue. In the previous version of manuscript, we provided the angle-resolved PL of a typical weakly-confined cavity in supplementary information as an example to demonstrate the weak parametric emission intensity. In the revised version of manuscript, as shown in Fig. R14 we provide the correspondent angle-resolved PL spectra for the sample in Fig. 2f and change to similar colormap for consistency. This sample has comparable detuning

energy with the one in Fig. 2a and its parametric emission intensity is significantly smaller than the strongly confined one in Fig. 2a.

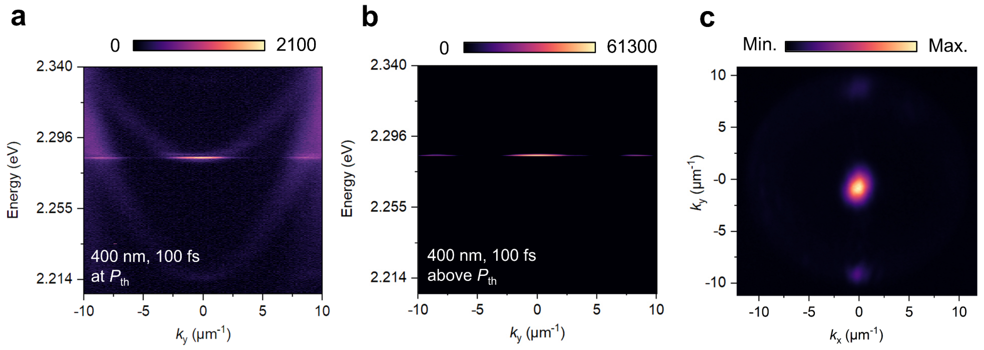

Fig. R14. (a) Parametric scattering in a weakly confined microcavity at threshold. (b) Parametric scattering in a weakly confined microcavity above threshold. (c) k-space image of for (b), showing bright condensate emission and weak parametric emission.

For the comparison of coherence, we initially present these two figures here to compare the coherence between a strongly confined microcavity and a weakly confined microcavity *at comparable parametric emission intensity*, to demonstrate that the strongly confined sample is a more suitable platform for developing coherent parametric light emission. Because the parametric emission in the weakly confined microcavity is usually weaker, we increased the pump power to 3 times the condensation threshold to obtain a stronger parametric emission from the weakly confined microcavity. This unavoidably led to linewidth broadening and a reduction of coherence as we discussed in the main text. To make the comparison more rigorous and illustrate the idea more straightforward, we included the extracted source interference profile for them and added the case of 1.5 P_{th} for weakly confined microcavities as shown in Fig. R15, into supplementary information. At similar pump fluence, the source visibility is at comparable level. Whereas the parametric emission in a strongly confined cavity preserves a higher level of coherence as demonstrated by a stronger visibility in region of interest. Furthermore, when pump fluence reaches $3P_{th}$ to have the comparable parametric emission intensity with that of the strongly confined sample, a drop in source visibility could be seen, the accompanied drop in parametric emission visibility is also shown as discussed in main text.

Revision 1.2:

Main text, page 6, revised as In contrast, for the weakly confined microcavity, when it was pumped at similar level (1.5 P_{th}), parametric emission is rather weak (Fig. S15).

SI, page 15, added Fig. S15 showing dispersions for the sample in main text Fig. 2f and included near threshold image to show the emergence of parametric emission from the LPB dispersion.

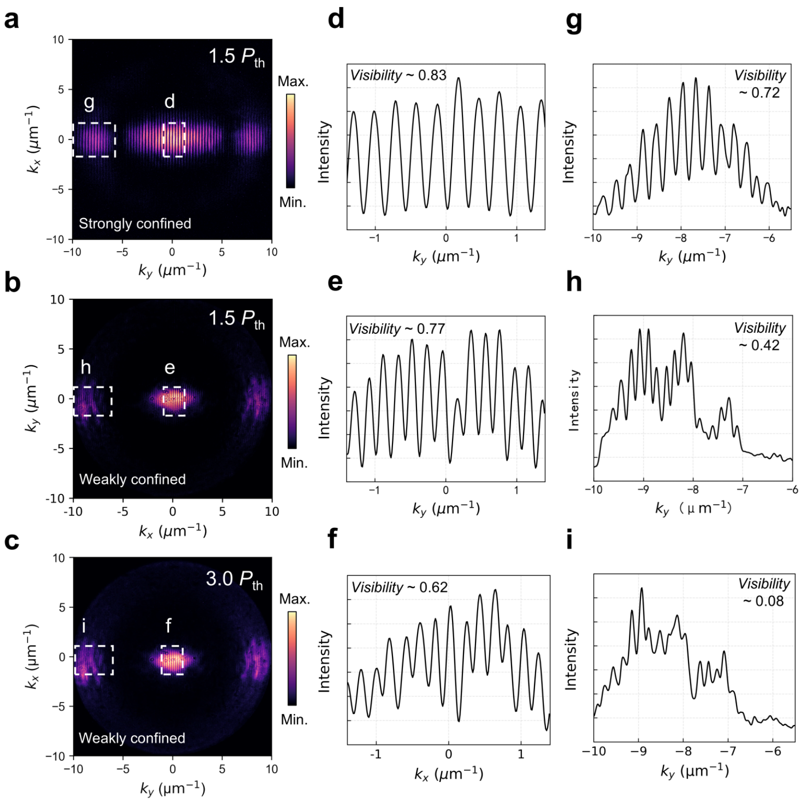

Fig. R15. (a) Interferometry patterns of parametric polaritons in a strongly confined microcavity at 1.5 P_{th} (condensation threshold). (b) Interferometry patterns of parametric polaritons in a weakly confined microcavity at 1.5 P_{th}. (c) Interferometry patterns of parametric polaritons in a strongly confined microcavity at 3.0 P_{th}. (d-f) Respective intensity profiles extracted from the interferometric images circled by the white dashed line highlighted 'source' condensate area. d: strongly confined, 1.5 P_{th}. e: weakly confined, 1.5 P_{th}. f: weakly confined, 3.0 P_{th}. (g-i) Respective intensity profiles extracted from left panel interferometric images circled by the white dashed line highlighted 'signal + idler' area. g: strongly confined, 1.5 P_{th}. h: weakly confined, 1.5 P_{th}. i: weakly confined, 3.0 P_{th}.

Revision 1.2:

Main text, page 5, revised as: ‘To directly visualize the superiority of strong parametric emission with preserved coherence in quantum confinement regime, we compare the *k*-space interferometry in a strongly confined microcavity and a weakly confined cavity (both dressed with disorder) at time delay of ~ 0.03 ps.’.

Main text, page 6, revised as: ‘Fig. 2d shows the momentum space interferogram for a strongly confined microcavity at 9 μJ·cm^{-2} (1.5 P_{th})’.

Main text, page 6, revised as: ‘Such visibility demonstrates strong phase-coherence between signal and idler polaritons in our microcavities. In contrast, for the weakly confined microcavity, when it was pumped at similar level (1.5 P_{th}), parametric emission is rather weak (Fig. S15). Therefore, higher pump fluence (20 μJ·cm^{-2}, 3.0 P_{th}) is needed for the generation of strong parametric emission comparable with the strongly confined microcavities. Such pump fluence inevitably leads to dephasing processes and broadens of the linewidth of signal and idler polaritons (Fig. 2f and 2g, Fig. S16).’

Main text, Fig. 2 caption, revised as: ‘(d) *k*-space interferometry patterns of parametric polaritons in a strongly confined microcavity (9.0 μJ·cm^{-2}, 1.5 P_{th}), where clear interference fringes appear for superimposed signal and idler polaritons. Line profiles in dashed rectangle area are plotted in (e) and a visibility of 0.72 could be extracted. (f-g) Respective interferometry and line profiles in a weakly confined microcavity (20.0 μJ·cm^{-2}, 3.0 P_{th}), when parametric emission is of comparable intensity to that in (d). Poor visibility is observed for superimposed signal and idler polaritons.’

SI, page 16, added Fig. R15 as Fig. S16.

Comment 1.3:

Moreover, the reported efficiency value—while I appreciate the large statistical analysis and the effort put into developing a suitable model—also appears problematic. The claimed efficiency seems to exceed the theoretical limit for a parametric process, in which the combined intensity of the signal and idler cannot surpass that of the pump. This suggests that there may be an issue in how the overall intensity of the condensate is being evaluated.

Response 1.3:

We thank the reviewer for raising concerns on the evaluation of 'ratio'. We agree with the reviewer that the theoretical limit of parametric emission intensity should not be higher than the pumping intensity which is related to applied laser pump fluence. While herein the "source" intensity is attributed to the source condensate intensity which would be much lower than the applied non-resonant pump laser, thus an above-unity emission ratio is possible considering the multiple population and depletion mechanisms involved. An above-unity parametric ratio indicates a stimulated regime with depletion of the source state and suggests highly efficient nonlinear parametric emission, a characteristic that has likewise been reported in pure-photonic OPOs (*Opt. Lett.* **21**, 1336–1338 (1996), *Opt. Lett.* **26**, 620–622 (2001)). And the depletion of pump/source polariton has also been observed in intrabranch polariton OPOs (*Phys. Rev. Lett*. **90**, 106401 (2003), *Phys. Rev. Lett.* **91**, 107402 (2003)) in the stimulated regime. In addition, exciton reservoir might provide small amount of seeding in this nonlinear regime, triggering the stimulated process to reach larger population. In our theoretical study, the above-unity ratio has also been observed as shown in data points in Fig. 3a.

In our microcavities in an energy-degenerate interbranch parametric scattering process, components near $k_y = 0$ would serve as the most effective source to create parametric polariton pairs at opposite in-plane wavevectors, due to the highly satisfied phase matching condition. Therefore, we extracted the spectra from central regions (integrated over 7 pixels in the angular direction) of the respective states (Fig. R16), considering that the source emission is broadened due to spatial confinement. On the other hand, the 'ratio' values for some literatures (*Nano Lett.* **22**, 2023–2029 (2022), *Phys. Rev. B* **106**, L020301 (2022), *Phys. Rev. B* **107**, L060303 (2023), *Nano Lett.* **22**, 3026–3032 (2022)) are not directly mentioned, therefore we extract the value (maximum) by reading the RGB color in the color bar. For the literature (*Nature* **639**, 337–341 (2025), *Nano Lett.* **21**, 3120–3126 (2021), *Phys. Rev. Lett.* **108**, 166401 (2012)) that directly gave the value, we utilized their given values and checked in respective angle-resolved plots that the values are not underestimated.

According to reviewer's suggestions, we have revised the term in main text from '*efficiency*' exclusively into '*parametric ratio*', and highlighted the feasibility of above

-unity ratio, the method for extracting parametric ratio in the revised version of manuscript and SI. While we keep the title as ‘highly efficient’, since the general parametric emission intensity is still higher than it is normally observed in planar microcavities.

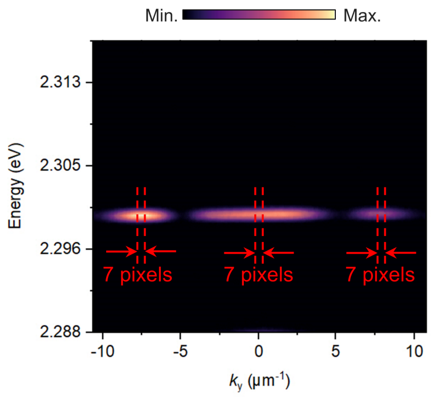

Fig. R16. Angle-resolved PL spectra showing areas used for integration and evaluation of parametric ratio. The angle resolved emission collected from the objective (100x, NA=0.9) was mapped to 166 pixels (angular direction) on the Fourier plane in the spectrometer. The emission centered around (red dashed lines) the respective states with 7 pixels were integrated in the angular direction for evaluation.

Revision 2:

Main text, page 4, revised as ‘In the energy-degenerate interbranch parametric scattering process, components near $k_y = 0$ would serve as the most effective source to create parametric polariton pairs at opposite in-plane wavevectors, due to the highly satisfied phase matching condition[18-21]. Therefore, we extracted the spectra from central region spectra of the respective states (Fig. S9), considering that the source emission is broadened due to spatial confinement[7].’

Main text, page 5, added ‘The above-unity signal-to-source ratio suggests depletion of the source state in the stimulated regime and indicates highly efficient nonlinear parametric process, a characteristic that has likewise been reported in pure-photonic[22,23] OPOs. The depletion of source state has also been observed in polariton OPOs in the stimulated regime[24,25].In our theoretical study, an above-unity ratio has also been observed as presented in the following discussion (Fig. 3a).’

Main text, page 6, Fig. 2b caption, added: 'A comparison of parametric emission ratio and linewidth of our work with reported literature values (based on given data[10,11,23] or estimated from color-scale maximum[13-16]).'

SI, page 9, added Fig. S9 on Schematic for extraction of spectrum in Fig. 2a, and evaluation of parametric ratio.

Comment 3:

In the introduction, the authors cite the work from J. Wu et al. (10.1021/acs.nanolett.1c00283), stating that the reported case exhibits suboptimal parametric emission efficiency and that pump powers "several times" above the condensation threshold are required to observe parametric scattering. However, upon reviewing the referenced paper, it is clear that parametric scattering is already visible at pump powers 2–3 times above the condensation threshold. This would more accurately be described as "a few times," rather than "several," and does not support the implication of particularly poor efficiency.

Response 3:

We thank the reviewer for pointing out the relative comparison of second threshold compared to the first threshold at condensation should be more appropriately described as 'few time'. We have revised the description in manuscript.

Revision 3:

Main text, page 3 revised from 'several' to 'a few times'.

Comment 4:

The authors used the same sample in one of their recent publications to observe polariton condensation under CW excitation. Why a pulsed excitation scheme was chosen for the present experiments? Could the authors show how the excitation regime (CW vs. pulsed) influences the key observed properties—such as parametric efficiency, linewidth, and coherence? Are these parameters intrinsic to the system or strongly dependent on the excitation conditions?

Response 4:

We thank the reviewer for recognizing our recent work on CW pumped polariton condensation. Here in this study, we utilized $CsPbBr_3$ microplatelets with much larger thickness (1-3 μm) to induce multiple LPBs and support the configuration of energy degenerate interbranch parametric scattering. The fine energy modes induced by surface disorder could also be observed, whereas the features of condensation (macroscopic population towards ground state, nonlinear intensity growth, linewidth narrowing, *etc.*) were not observed in CW pumped case. Such difference is because thicker samples support multiple LPB modes, and relaxation into multiple branches competes mutually, thereby making it difficult for relaxation and bosonic final-state stimulation to favor a specific polariton mode. Therefore, to achieve polariton condensation and to initiate the nonlinear interbranch parametric scattering, femtosecond pulsed excitation is required in this case.

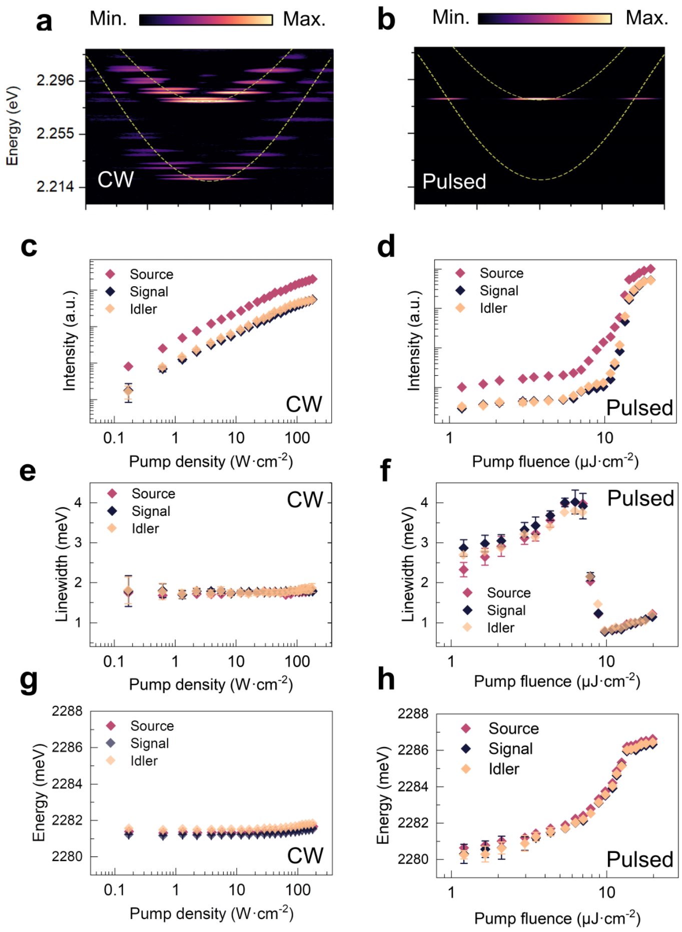

Fig. R17. The emission properties of a typical microcavity under CW and pulsed excitation condition. (a) Angle-resolved PL spectra under CW excitation. (b) Angle-resolved PL spectra showing condensation and nonlinear parametric emission, under pulsed excitation. (c, d) Pump density dependent evolution of source, signal and idler

emission intensity for CW (c) and pulsed (d) excitation, respectively. (e, f) Pump density dependent evolution of linewidth of source, signal and idler emission for the CW (e) and pulsed (f) excitation, respectively. (g, h) Pump density dependent evolution of blueshift of source, signal and idler emission peaks for the CW (g) and pulsed (h) excitation, respectively.

As shown in Fig. R17 for the comparison for an identical microcavity under CW (a, c-e) and pulsed excitation (b, f-h). The population under CW excitation (a) distributes over a large energy range, while in pulsed pumped case (b) at high pump fluence, the emission from source/signal/idler dominates the spectrum. Under CW excitation condition, the intensity of signal/idler emission was sublinear with pump density (c), which is identical to that of pulsed excitation below condensation threshold (f). While for pulsed excitation, the intensity of signal/idler emission exhibits few transitions, related to source condensation and nonlinear parametric scattering. Furthermore, the linewidth of signal/idler emission for CW excitation (d) is almost constant throughout the measured pump density ranges. While for pulsed excitation, the linewidth (g) and blueshift (h) exhibit evident changes. These results suggest that although the observed feature of coherent parametric emission is endowed by disorder, pulsed excitation is required to induce the nonlinear process.

Comment 5:

Regarding the surface-smooth sample shown in Fig. S9, it is unclear why the discretization of the TE1 and TM1 modes is not visible. **Given that this sample is used as a key comparison point**, the absence of clear mode discretization raises questions about the confinement quality. As mentioned earlier, to convincingly support their claims about the role of disorder, the authors should identify a surface-smooth sample with good phase matching, similar lateral dimensions, and comparable detuning.

Response 5:

We thank the reviewer for raising the respective concerns related to Fig. S9. The comment on the need of a surface-smooth (weak disorder) sample to do the comparison is included in Response 1. In previous Fig. S9, the presentation of the dispersion below threshold is not well presented to show cavity confinement quality, due to the mix of polarization, and that the condensation occurs on a LPB close to exciton which

diminishes the mode quantization feature. We now present polarization split results for a thick confined cavity (L_y =3.7 μm; L_x = 12.6 μm) and fit the LPB dispersion to show strong coupling and mode quantization features, as shown in Fig. R18. The yellow/green/white dashed lines represent the coupled-oscillator model fits to the three LPBs. In Fig. 18c, we list the respective experimental and calculated parameters for the TM modes. The as-measured mode spacings are comparable with calculated values $\Delta E \approx \frac{3\hbar^2\pi^2}{2m^* L_y^2}$ for LPB2 and LPB3. For LPB1, however, the effective mass is large, so the mode spacing is further reduced to 3.98 meV, close to its linewidth of 3.0 meV (broadened when approaching exciton resonance). Therefore, the quantization of the LPB1 can no longer be resolved.

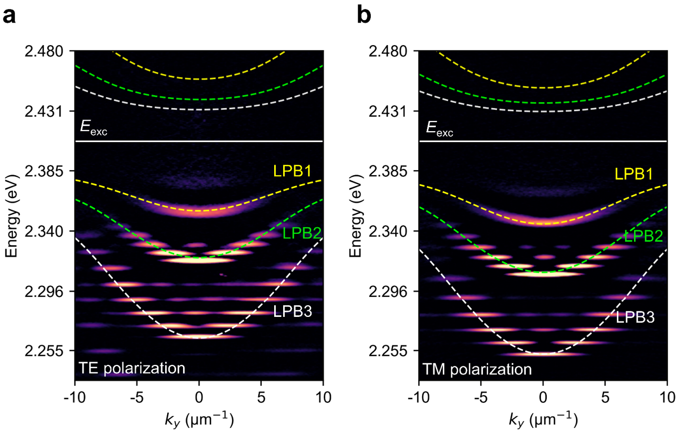

c

TM polarization	LPB1	LPB2	LPB3
Rabi splitting (meV)	119	120	127
Effective mass (*m_ex 10^{-5})	2.031	1.404	1.160
$\Delta E_{1,2}$ (meV, experiment)	/	6.0	7.2
$\Delta E_{1,2}$ (meV, calculation)	3.98	5.75	6.96

Fig. R18. Exciton-photon coupling in a strongly confined microcavity (L_y =3.7 μm; L_x = 12.6 μm) by a CW 405 nm laser. (a) Angle-resolved PL spectra for TE polarization. The dashed lines: disperison fitting for LPBs based on coupled oscillator model. (b) Angle-resolved PL spectra for TM polarization with dashed lines fitting the respective LPBs. (c) Fitted Rabi splitting, fitted LPB effective mass, measured energy spacing and calculated energy spacing for LPB1, LPB2 and LPB3 lowest two energy levels shown in (b), respectively.

Revision 5:

Main text, page 3, added 'The micron-scale thickness of MPs enables multiple sets of lower polariton branches (LPBs) which further split into transverse electric (TE) and transverse magnetic (TM) branches due to material birefringence[28]. The coupled

oscillator model fitting in both polarizations are depicted in Fig. S7, showing strong coupling characteristics.'

SI, page 7, added Fig. S18 as Fig. S7 and discussed the respective evaluation of energy levels associated with confinement. Discussed the TM branch quantization features and the reason of missing quantization features for some LPB which are close to exciton.

Comment 6:

How are the overall intensities of the signal, idler, and source evaluated? Part of the condensate signal may propagate outside the excitation region and thus be excluded from the intensity measurement. If so, this could lead to an underestimation of the total condensate power and therefore to high efficiency. Additionally, the authors should provide an estimate of the experimental uncertainty associated with the efficiency ratio evaluation.

Response 6:

We thank the reviewer for the constructive suggestions on a clearer description on experimental measurement and a more accurate analysis of experimentally measured and extracted parametric 'ratio'. We included the approach we estimate the 'ratio' in response to comment 1.3. Next, we would like to explain the experimental setup. The microcavities were excited by a non-resonant pulsed (400 nm, 1kHz, 100 fs) laser with a beam spot size of ~ 50 μm (therefore samples are uniformly excited) and collected in a Fourier setup as sketched in Fig. S8. To enable spatial selection of the desired area, an adjustable rectangular spatial aperture was positioned at the confocal plane of the 1st and 2nd Fourier lens. When collecting the emission, we spatially selected the whole sample area therefore all the respective emissions from the sample were projected onto the Fourier plane, eliminating the possible propagation out of excitation/collection region and underestimation of condensate polariton intensity.

We also appreciate the reviewer's suggestion on estimation of experimental uncertainty in ratio evaluation. The uncertainty in experiments could be excluded here as discussed above. For data processing, we obtain the value from accumulated spectrum projected on 7-pixels in angular direction centering the emission, instead of from a single pixel spectrum, as we discussed in Response 2. In addition, such extraction would not affect

the relative intensity between signal and idler, thus ensuring negligible impact on the model in Fig. 3. Therefore, we expect that errors could be excluded here.

Revision 6:

Main text, page 4, added 'In the energy-degenerate interbranch parametric scattering process, components near k_y = 0 would serve as the most effective source to create parametric polariton pairs at opposite in-plane wavevectors, due to the highly satisfied phase matching condition[18-21]. Therefore, we extracted the spectra from central region spectra of the respective states (Fig. S9), considering that the source emission is broadened due to spatial confinement[7].'

Main text, page 5, added 'The above-unity signal-to-source ratio suggests depletion of the source state in the stimulated regime and indicates highly efficient nonlinear parametric process, a characteristic that has likewise been reported in pure-photonic[22,23] OPOs. The depletion of source state has also been observed in polariton OPOs in the stimulated regime[24,25].In our theoretical study, an above-unity ratio has also been observed as presented in the following discussion (Fig. 3a).'

SI, page 9, added Fig. S9 on Schematic for extraction of spectrum in Fig. 2a, and evaluation of parametric ratio.

Comment 7:

The authors emphasize that the presented configuration enables the generation of balanced signal and idler beams. However, in Fig. 2a, the idler appears to be approximately three times weaker than the signal. The authors should clarify the origin of the imbalance.

Response 7:

We thank the reviewer for suggestion on adding the explanation of intensity imbalance in the discussion. The unbalanced intensity of signal and idler is induced by *asymmetry in both population and depletion pathways* in the driven-dissipative system, which are further magnified in presence of surface disorder that breaks inversion symmetry. Such imbalance existing even in the nonlinear regime has been theoretically discussed (*Phys. Rev. B* **93**, 064203 (2016)) and experimentally observed in polariton nonlinear emission modulated by trap or disorder (*Phys. Rev. Lett.* **104**, 126403 (2010), *Nat. Commun.* **13**,

3080 (2022)). The emission intensity reflects averaged gain and loss at signal/idler in the driven dissipative non-equilibrium system. Exciton relaxation and scattering from the source populate the signal and idler states, whereas radiative recombination and relaxation into LPB2 lower energy states act as loss channels. Due to broken inversion symmetry, the energy levels and scattering pathways become intrinsically asymmetric, which distort these linear processes and bias the subsequent stimulated scattering and population in nonlinear regime, resulting in the imbalanced emission as we observed. Furthermore, a nonlinear and driven–dissipative behavior of the parametric oscillator could be reflected in the evolution of the energy mismatch in the nonlinear regime. Above the nonlinear parametric emission threshold (Fig. R19), the mismatch tends to saturate, while the linewidth continues to broaden, revealing that nonlinear interactions gradually drive the populations towards a dynamic equilibrium and pin the mismatch to an almost constant value. In addition, in a theoretically simulated nonlinear parametric oscillator as we show in Fig. S23, the emission intensity is unbalanced as well, supporting that the imbalance is theoretically applicable.

According to your suggestion, we have emphasized the mechanism for the imbalanced signal and idler emission in the revised version of manuscript.

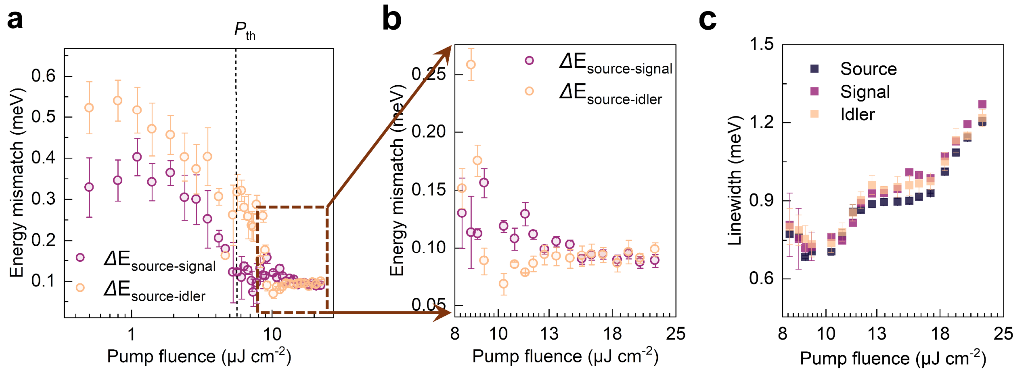

Fig. R19. (a) Evolution of source to signal, and source to idler energy mismatch with pump fluence from 0.3 to 27.5 μJ·cm^{-2}. The dashed line indicates condensation threshold. (b) Zoom-in view of energy mismatch in dashed circled area from 8.0 to 25.0 μJ·cm^{-2}. (c) Corresponding linewidth broadening for source, signal, and idler, with pump fluence from 8.0 to 25.0 μJ·cm^{-2}. The source to signal, and source to idler mismatch tend to saturate, accompanied by continuous broadening in linewidth.

Revision 7:

Main text, page 5, added: 'An imbalance in the intensity of signal and idler emission could also be observed, which is expected in these microcavities dressed with disorder and confinement[3,26,27], considering the asymmetry in both population and depletion pathways in the driven-dissipative system.'

Comment 8:

For the non-confined microcavities below threshold dispersion should be shown.

Response 8:

We thank the reviewer for the suggestion of adding below threshold dispersion for weakly confined microcavities. We made the respective revision as discussed in Response 1.2.

Revision 8:

Main text, page 6, revised as In contrast, for the weakly confined microcavity, when it was pumped at similar level (1.5 P_{th}), parametric emission is rather weak (Fig. S15).

SI, page 15, added Fig. S15 showing dispersions for the sample in main text Fig. 2f and included near threshold image to show the emergence of parametric emission from the LPB dispersion.

Response to Reviews (NCOMMS-25-52756-B)

Response to Reviewer 1

Overall comment:

The authors have thoroughly addressed all points raised during the first round of revisions. The revised manuscript and improved Supplementary Information will be valuable to the community, and I therefore recommend publication.

Response:

We sincerely thank the reviewer for their time and careful review of our manuscript, and we appreciate the reviewer's support for the publication of our work.

Comment 1:

One minor point remains regarding terminology. The manuscript uses the term "disorder" in a way that is ambiguous, since disorder can refer to multiple mechanisms (morphological, crystallographic, compositional, or energetic). In the present context, the effect being discussed is specifically due to surface or interface roughness. I recommend replacing "disorder" with something more specific, like roughness. This will make it more clear for the reader that the disorder being discussed here is morphological.

Response 1:

We thank the reviewer for pointing out the potential ambiguity of the term "disorder" for a general readership.

We utilized the terminology to be more consistent with other polariton literatures (*Nat. Commun.* **12**, 4839 (2021), *Phys. Rev. Lett.* **96**, 066405 (2006), *Phys. Rev. B* **73**, 241308(R) (2006)), where '*disorder*' is often used as a general term to describe the variation of an effective potential energy, i.e., static spatial fluctuations of the photonic/polaritonic energy that break translational symmetry and lead to localization and energy level quantization. The terminology '*roughness*' describes the morphological origin that might not fully convey the potential energy variations experienced by polaritons and potential configurations used in our mathematical modeling framework. Therefore, we consider it more appropriate to retain the term "disorder" when referring to the effective photonic energy landscape in our microcavities.

To avoid ambiguity for a general readership, we revised the manuscript to define the terminology explicitly at first use, stating that throughout the manuscript "disorder" refers only to morphology-induced surface/interface roughness and the corresponding roughness-induced disorder potential energy landscape.

Revision 1:

Main text, page 2, revised as 'Dispersion characteristics enabled by surface disorder (refer to morphological roughness, in this context) and geometric confinement allow enhanced parametric interactions.'

Response to Reviewer 2

Overall comment:

First, I appreciate the authors' impressive efforts, including additional experiments, which enabled them to address the concerns raised by the reviewers in the previous stage. In particular, in the refactored text, the authors address previously missing critical points: explicitly define terms, identify the dominant parametric scattering mechanism of the observed coherent emission, and distinguish the roles of confinement and roughness in achieving high emission efficiency. The authors' claims and conclusions are now well supported by the data and analysis presented in the manuscript. In its current form, the work constitutes a valuable contribution to nonlinear polaritonics. I therefore strongly support its publication in Nature Communications.

Response:

We sincerely thank the reviewer for carefully reviewing our manuscript and strongly support publication of our work.

Comment 1:

After carefully reading the updated text, I have two minor suggestions to further improve the paper's clarity: The authors define a specific threshold value (4 μm) for confinement, which is likely structure-specific. To be more general and material-platform-independent, one could define a strongly confined system as one capable of supporting a parametric ratio greater than 1.

Response 1:

We thank the reviewer for suggesting a definition of 'strongly confined' with more physically related quantities, rather than limited to material-platform related aspects. In the counted parametric ratios in Fig. 3, a parametric ratio greater than 1 appears when confinement reaches down to ~4.5 μm. Following the suggestion, we therefore revise the threshold value for confinement from 4 μm to 4.5 μm and added respective descriptions for the value when first define it in main text.

Revision 1:

Main text, page 3, revised as 'Since the high-efficiency parametric emission (with a parametric ratio of larger than 1 as shown in later discussions) emerges when L_y decreases to 4.5 µm as shown in later discussions, we define the regime where L_y is 'smaller than' 4.5 µm as 'strongly confined' and L_y is 'larger than' 4.5 µm as 'weakly confined', respectively'.

Comment 2:

It is worth specifying the sizes of the pump spot and the spatially filtered detection region, since both may affect the observations. Accordingly, a long focal distance lens may be missing in the excitation channel in Fig.S6.

Response 2:

We thank the reviewer for pointing out the need to specify the sizes of the pump spots. As mentioned by the reviewer, a long focal distance lens was placed in the excitation path, to enable an enlarged laser spot (~50 µm) for (a) spatially uniform exciton reservoir generation in non-resonant excitation and (b) focused spot size in Fourier space.

We added the long focal distance lens in Fig. S6 and respective discussions on pump spot size and spatially filtered region by the aperture.

Revision 2:

SI, page 6, Fig. S6, revised as:

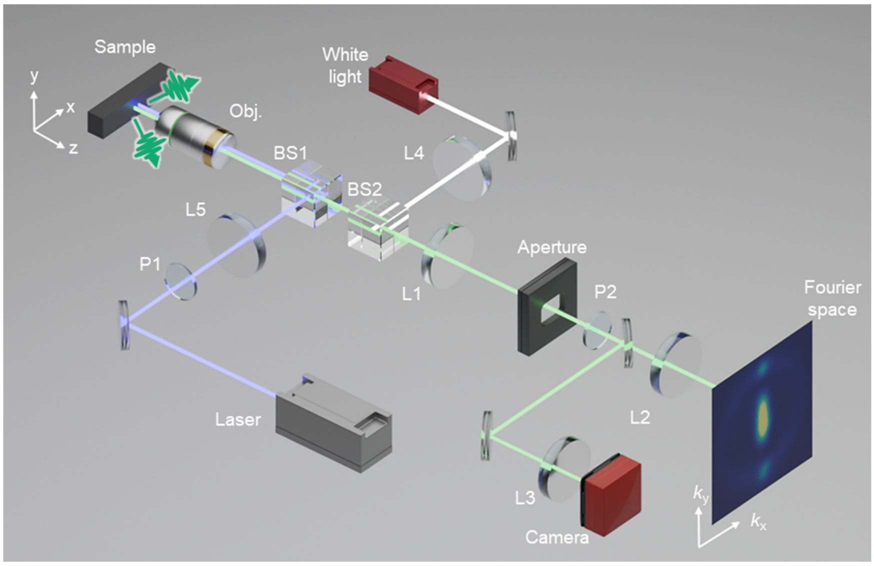

Fig. R1. $CsPbBr_3$ microplatelet (gray cube in upper left corner) and respective signal and idler polariton (green arrows near the sample, and bright high-*k* spots in Fourier space) are depicted. (Obj: objective, L: lens, P: polarizer, BS: beam splitter) L5 denote the long focal distance lens utilized for enlarging the spot size to ~50 µm. An adjustable rectangular aperture was used to spatially select the emission from the entire MP microcavity except its edges, with cavity dimensions ranging from ~2 µm to ~15 µm, as shown in Fig. 3.

Main text, Methods, revised as 'Pump laser spot size was enlarged by a long focal distance lens in the excitation path, as shown in Fig. S6. Emission from the sample was captured with a 100× objective (NA = 0.9) and spatially filtered with an adjustable rectangular aperture at the focal plane and directed to a liquid nitrogen-cooled charge-coupled device (CCD) spectrometer'.

Response to Reviewer 3

I co-reviewed this manuscript with one of the reviewers who provided the listed reports. This is part of the Nature Communications initiative to facilitate training in peer review and to provide appropriate recognition for Early Career Researchers who co-review manuscripts.

Response:

We thank the reviewer for co-reviewing our manuscript. The comments are responded with point-to-point responses and revisions in manuscript.

Response to Reviewer 4

Overall comment:

The authors have carefully addressed the concerns raised in my previous report and have substantially strengthened the manuscript through additional experiments and a clearer discussion. In particular, the role of disorder is now supported by more appropriate reference samples with comparable confinement, detuning, and phase-matching conditions, and the comparison between strongly confined and weakly confined microcavities is significantly improved. The clarification regarding the definition of the parametric ratio and the distinction from pump efficiency also resolves the main conceptual ambiguity……Apart from this point, the main scientific issues raised in the previous review have been addressed, and the manuscript is close to being suitable for publication.

Response:

We thank the reviewer for carefully reviewing our revised manuscript and appreciate the reviewer’s strong support of the results and discussions in responses to the major issues raised in the first round of review.

Comment 1:

In addition, I note that further measurements requested by other referees (such as resonant excitation experiments) have been successfully implemented and add important information. At this stage, I believe that only one additional clarification would further consolidate the physical picture. Since the manuscript now explicitly discusses TE and TM mode quantization and presents their respective dispersions, as requested in the previous review, it would be highly valuable to demonstrate whether parametric scattering under resonant excitation can be achieved by pumping a discretized localized mode rather than the ground state where condensation occurs.

Specifically, with reference to Fig. R18, it would be important to clarify whether parametric scattering can be realized within the same polariton branch by resonantly exciting one of the higher-energy localized states at zero in-plane momentum. As phase matching for these higher-energy states is satisfied in both LPB2 and LPB3, as well as in both TE and TM polarizations, demonstrating parametric scattering under these conditions would provide a stringent test of the proposed mechanism.

Response 1:

We thank the reviewer for proposing the experiments to support the mechanism of matched energy levels, making use of the discretized higher energy states in the same LPB with intrinsically matched energy positions to satisfy phase matching condition (*Phys. Rev. B* **87**, 155302 (2013)), a prerequisite for parametric interactions.

In Fig. R2 we show the schematic of resonant pumping on such higher energy levels. Pulsed laser was filtered to match the energy and tuned to normal incidence to resonantly pump central 'd' state as indicated by the blue circle in Fig. R2a. Below threshold as shown in Fig. R2b, only reflected laser is observed and polaritonic emission was absent. Above threshold, central 'd' state was resonantly injected and masked by the strong reflected laser as shown in the central saturated part. Two additional high k emissions were observed, indicating the occurrence of such parametric scattering as schematically shown in Fig. R2a.

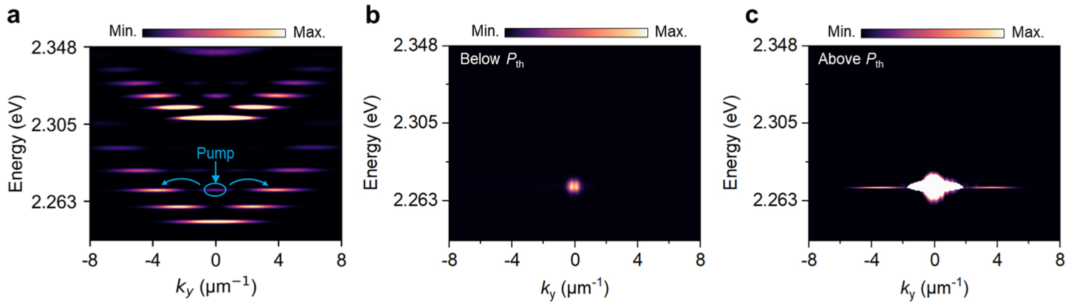

Fig. R2. Schematic for resonant pump induced parametric scattering on higher energy levels. (a) Angle-resolved PL spectra in the linear regime. Blue circle highlights position for resonant pump laser, while the arrows indicate parametric scattering on the energy degenerate states on the same LPB. (b) Angle-resolved spectra below polariton emission threshold, where only reflected laser signal is observed. (c) angle-resolved spectra above polariton emission and parametric scattering threshold.

In Fig. R3 we summarized the resonant pump results on LPB2 and LPB3 for both TE and TM polarizations, as suggested by the reviewer. Fig. R3 (a) and (d) show the linear regime PL spectra as discussed in Fig. S7, with 'd' orbital states highlighted by dashed green or white rectangles utilized for resonant pump induced parametric emission . As shown in Fig. R3b, in TE polarization, resonant pump on LPB2 results in emergence of two high-k states, demonstrating typical parametric scattering. The source emission

being resonantly injected was obscured by strong reflected laser which was co-polarized with the TE mode polaritons.

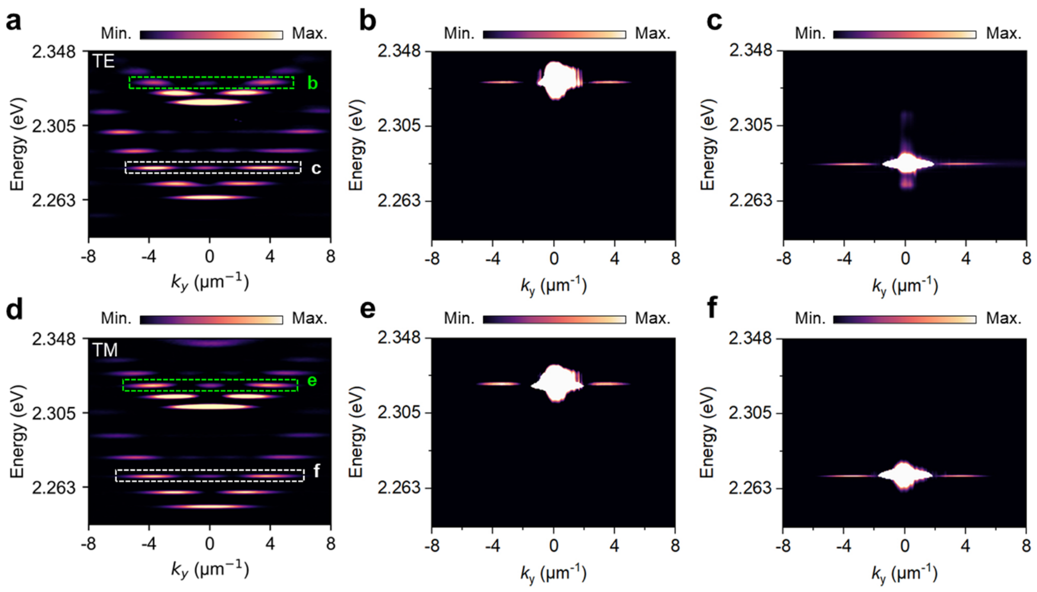

Fig. R3. Resonant pump induced parametric scattering on higher energy levels. (a) Angle-resolved PL spectra in the linear regime for TE polarization, as the one shown in Fig. S7a. Green and white dashed rectangle highlight the 'd' orbital states LPB2 and LPB3, respectively, for energy degenerate parametric process. (b) Resonant pump induced parametric scattering in TE polarization on LPB2. (c) Resonant pump induced parametric scattering in TE polarization on LPB3. (d) Angle-resolved PL spectra in the linear regime for TM polarization, as the one shown in Fig. S7b. (e) Resonant pump induced parametric scattering in TM polarization on LPB2. (f) Resonant pump induced parametric scattering in TM polarization on LPB3.

The same occurs for LPB3 in TE polarization and LPB2 and LPB3 in TM polarizations, respectively, indicating that the matched energy levels enable polariton parametric interactions. We have added discussion on parametric emission within the same polariton branch according to the reviewer's suggestion.

Revision 1:

SI, page 20, Fig. S19 caption, added 'Such phase matching condition in confined microcavities has also been investigated by resonantly induced parametric scattering at higher energy levels within the same LPB, as reported in previous literature[15].
